# Superior anti-DLBCL efficacy of novel organic arsenical Z2-A-Z2 through ROS-mediated apoptosis and critical NF-κB/IκBα signaling pathway inhibition

**DOI:** 10.1186/s13046-026-03724-4

**Published:** 2026-05-18

**Authors:** Wenjiao Wei, Yanni Ma, Yujiao Liu, Xian Zhang, Shuangnian Xu, Qingfeng Li, Dongdong Zhang

**Affiliations:** 1 Department of Oncology, Postgraduate Union training base of Xiangyang No.1 People’s Hospital, Hubei University of Medicine, Xiangyang, Hubei 441000 China; 2 Center of Hematology, Key Laboratory of Tumor Immunotherapy of Chongqing, Southwest Hospital, Third Military Medical University (Army Medical University), Chongqing, 400038 China; 3 State Key Laboratory of Separation Membranes and Membrane Processes, School of Chemistry, Tiangong University, Tianjin, 300387 China; 4 Department of Hematology, Postgraduate Union Training Base of Xiangyang No.1 People’s Hospital, School of Medicine, Wuhan University of Science and Technology, Xiangyang, 441000 China; 5 Department of Oncology, Xiangyang Central Hospital, Hubei University of Arts and Science, Xiangyang, Hubei 441000 China

**Keywords:** DLBCL, Organic arsenical, Z2-A-Z2, NF-κB, Mitochondrial ROS

## Abstract

**Objective:**

Diffuse large B-cell lymphoma (DLBCL) often resists therapy due to aberrant activation of NF-κB signaling pathway. We synthesized and evaluated Z2-A-Z2, a novel organic arsenical, hypothesized to target this survival pathway, aiming to assess its efficacy and mechanism against DLBCL.

**Materials and methods:**

The novel compound Z2-A-Z2 was synthesized and verified by ^1^H CMR, ^13^C CMR, and high-resolution mass spectrometry. Its anti-proliferative activity was assessed in GCB- and ABC-subtype DLBCL cell lines and normal peripheral blood mononuclear cells (PBMCs) using CCK-8 assays. Mechanisms were elucidated through flow cytometry (apoptosis, ROS, ΔΨm), Western blot (apoptotic and NF-κB pathway proteins), and enzyme activity assays. The causal role of ROS was confirmed by N-Acetylcysteine rescue (NAC) experiments, and the criticality of NF-κB inhibition was validated using *p65* overexpression and pharmacological inhibition. Efficacy and safety were ultimately verified in SU-DHL-6 and U2932 xenograft model.

**Results:**

Z2-A-Z2 demonstrated potent and selective anti-proliferative activity against DLBCL cell lines, achieving an average IC₅₀ of approximately 1.1 µM. This efficacy was 3- to 6-fold superior to arsenic trioxide (ATO), while exhibiting minimal cytotoxicity toward normal PBMCs and B cells. Mechanistically, Z2-A-Z2 induced profound mitochondrial-mediated apoptosis and G2/M cell cycle arrest through a unique dual action of simultaneously triggering catastrophic oxidative stress and critically suppressing the pro-survival NF-κB/IκBα signaling axis. Further investigation revealed a bidirectional regulation where ROS acts upstream to suppress p65 activity, while p65 in turn modulates drug-induced ROS generation. In vivo studies further validated these findings, showing that Z2-A-Z2 resulted in a robust suppression of tumor growth in a xenograft model, accompanied by diminished NF-κB/IκBα activity and increased apoptosis, alongside an excellent safety profile.

**Conclusion:**

Z2-A-Z2 is a promising organic arsenical with superior efficacy and safety over ATO. Its unique dual-action strategy of simultaneously inducing oxidative stress and critically inhibiting the NF-κB/IκBα signaling axis, positioning it as a strong clinical candidate for effectively treating DLBCL.

**Supplementary Information:**

The online version contains supplementary material available at 10.1186/s13046-026-03724-4.

## Introduction

Diffuse Large B-cell Lymphoma (DLBCL) is the most common subtype of non-Hodgkin lymphoma (NHL), comprising up to 40% of all cases [1]. While the standard first-line R-CHOP chemoimmunotherapy regimen is curative for approximately 60% of patients, a significant portion experiences relapsed or refractory (R/R) disease, leading to poor clinical outcome [2]. Although novel treatments like chimeric antigen receptor T-cell therapy have shown promise, their broader application is hindered by significant toxicities, high costs, and complex logistics [3, 4]. The inherent biological heterogeneity of DLBCL and high-risk genetic features, further underscores the urgent need for novel therapeutic strategies [5] .

The aggressive phenotype of DLBCL, particularly the activated B-cell-like (ABC) subtype, is frequently driven by constitutive activation of the Nuclear Factor κB (NF-κB) signaling pathway [6]. This pathway is a central regulator of tumorigenesis, promoting cell proliferation, inflammation, and survival while suppressing apoptosis [7]. In DLBCL, persistent NF-κB activity, often mediated by its key subunit p65 (RelA), is strongly associated with chemotherapy resistance, disease progression, and inferior survival [8]. Consequently, targeting p65 and inhibiting the NF-κB pathway represents a highly rational therapeutic approach to overcome drug resistance and induce apoptosis in DLBCL cells.

A parallel and complementary strategy for cancer therapy involves exploiting the intrinsic metabolic vulnerabilities of tumor cells, specifically their redox balance [9]. Malignant cells often exhibit elevated levels of reactive oxygen species (ROS) due to their high metabolic rate [10]. While moderate ROS levels can promote proliferation, excessive oxidative stress triggers catastrophic damage to DNA, proteins, and lipids, ultimately activating the mitochondrial pathway of apoptosis [11, 12]. Many effective anticancer agents, including the inorganic arsenical arsenic trioxide (ATO), function by inducing overwhelming ROS accumulation [13]. In DLBCL, leveraging oxidative stress to trigger apoptosis has been validated as a key anti-lymphoma mechanism [14] .

Although ATO is a cornerstone in the treatment of acute promyelocytic leukemia [15], its broader oncological application is severely hampered by a narrow therapeutic index, significant cardiotoxicity, and the emergence of drug resistance [16, 17]. To circumvent these liabilities, organic arsenicals have been developed as a superior chemical class [18]. Building upon our previous work with the organic arsenical A-Z2, which displayed enhanced anti-leukemic activity and a favorable safety profile compared to ATO [19], we have rationally designed and synthesized a novel derivative, Z2-A-Z2. This study investigates the hypothesis that Z2-A-Z2 exerts potent anti-DLBCL activity through a dual mechanism: the targeted inhibition of the pro-survival NF-κB pathway via p65, coupled with the simultaneous induction of lethal mitochondrial oxidative stress.

## Materials and methods

### Reagents

Arsenic trioxide (As₂O₃; #A1010) was purchased from Sigma-Aldrich. Doxorubicin (#E2516) and JSH-23 (#S7351) were obtained from Selleck Chemicals. N-Acetylcysteine (NAC; #S0077), penicillin-streptomycin solution (#C0222), and cell cycle detection kits (#C1052) were from Beyotime Biotechnology. Dimethyl sulfoxide (DMSO; #D8371-50) was from Solarbio. Cell Counting Kit-8 (CCK-8; #PMK0854), DAPI reagent (#PMK0064), JC-1 dye (#PMK0996), and H₂DCFH-DA fluorescent probe (#PMK0859) were from Biopmk.

### Antibodies

Primary antibodies used for Western blot analysis were as follows: PARP (#9542S), Caspase-3 (#9662), Caspase-9 (#9508S), BCL-2 (#4223) and MCL-1 (#4572) from Cell Signaling Technology (CST); BCL-2 (#60178-1-Ig), GAPDH (#60004-1-Ig), β-Tubulin (#10094-1-AP), p62 (#18420-1-AP), LC3 (#14600-1-AP), ATG5 (#10181-2-AP), IκBα (#66418-1-Ig), p50 (#14220-1-AP), p65 (#10745-1-AP), Ki67(#66555-6-Ig), Cleaved Caspase-3(#68773-1-Ig), HMGB1(#66525-1-Ig), KIM-1(#30948-1-AP) and NGAL(#26991-1-AP) from Proteintech. Beclin 1 (#A21191) and GABARAPL1 (#A23781) from ABclonal. IKKα (#S0995) and IKKβ (#S0333) were from Selleck. Pho-p65 (#YM8442) was from Immunoway.

### Synthesis of arsenicals Z2-A-Z2

The synthesis of the organic arsenical compound Z2-A-Z2 was performed in the following three steps:

Step a: Synthesis of 4-(1,3,2-Dithiarsinan-2-yl)aniline A solution of p-arsanilic acid (4 g, 18.4 mmol) in 70% ammonium thioglycolate (10 mL) was stirred at 50 °C for 2 h. Subsequently, 1,3-propanedithiol (2 mL) was added dropwise to the mixture, which was then stirred for an additional 2 h. The resulting turbid solution was extracted with dichloromethane (DCM). The combined organic extracts were concentrated under reduced pressure, and the residue was purified by silica gel column chromatography to yield 4-(1,3,2-dithiarsinan-2-yl)aniline.

Step b: Preparation of Azelaoyl Chloride Azelaic acid (0.94 g, 5 mmol) was dissolved in dry DCM (20 mL) under a nitrogen atmosphere. Oxalyl chloride and a catalytic amount of N, N-dimethylformamide (DMF) were then added, and the reaction mixture was stirred for 40 min. Following the reaction, the solvent was removed under reduced pressure to yield a light yellow solid.

Step c: Coupling Reaction to Form Z2-A-Z2 In an ice bath and under a nitrogen atmosphere, the light yellow solid from step b (4.5 mmol) was dissolved in DCM (10 mL). A separate solution of 4-(1,3,2-dithiarsinan-2-yl)aniline (1.23 g, 4.5 mmol), prepared in step a, in DCM (15 mL) was added, followed by the addition of pyridine (500 µL). The resulting mixture was stirred, and the final product was purified by silica gel column chromatography to afford Z2-A-Z2.

### Cell lines and culture conditions

The human GCB-subtype DLBCL cell lines SU-DHL-4 and SU-DHL-6 were purchased from Wuhan Sevier Biotechnology. The human ABC-DLBCL cell lines HBL-1 and U2932 were purchased from Hunan Fenghui Biotechnology and Guangzhou Ubigene Biosciences, respectively. These lymphoma cell lines and peripheral blood mononuclear cells (PBMCs) were cultured in RPMI-1640 medium (Gibco, #C11875500BT) supplemented with 10% or 20% fetal bovine serum (FBS) (Gibco, #10099-141), respectively. HEK293T cells (a kind gift from Prof. Xu, Southwest Hospital) were maintained in DMEM medium (Gibco, #C11885500BT) with 10% FBS (ExCell Bio, #FSP500). The HK-2 cell line (provided by Hubei University of Medicine) was cultured in DMEM/F12 medium (Gibco, #C11330500BT) with 10% FBS. All culture media were supplemented with 1% penicillin-streptomycin, and all cell lines were maintained at 37 °C in a humidified atmosphere containing 5% CO₂.

### Cell viability assay

Cells were seeded into 96-well plates at a density of 2 × 10⁴ cells/well. After overnight incubation, cells were treated with serial dilutions of Z2-A-Z2 (0–4 µM) or ATO (0–8 µM) for 24 h. Subsequently, 10 µL of CCK-8 reagent was added to each well, and the plates were incubated at 37 °C for 2–4 h. Absorbance was measured at 450 nm using a Varioskan Flash microplate reader (Thermo Fisher Scientific). The half-maximal inhibitory concentration (IC₅₀) values were calculated using GraphPad Prism 8.0 software.

### PBMC isolation and fluorescence-activated cell sorting

Human peripheral blood samples were collected into EDTA-anticoagulated tubes. PBMCs were isolated via density gradient centrifugation using Ficoll-Paque™ PLUS (Cytiva, #17-1440-03) according to the manufacturer’s instructions. Briefly, whole blood was diluted 1:1 with PBS, carefully layered over an equal volume of Ficoll, and centrifuged at 400 × g for 30 min at room temperature with the brake disengaged. The mononuclear cell interface was harvested, washed twice with PBS (300 × g, 10 min), and resuspended in chilled sorting buffer (PBS supplemented with 0.5% BSA and 2 mM EDTA) at a density of at 1 × 10⁷ cells/mL.

For immunophenotyping, cells were labeled with CD3-FITC (BioLegend, #300306), CD19-PE (BioLegend, #302208), and CD14-PerCP (BioLegend, #325631) for 30 min at 4 °C in the dark. Following incubation, cells were washed and resuspended in sorting buffer containing DAPI (1 µg/mL) for dead-cell exclusion. Cell sorting was performed using a FACS Aria™ III flow cytometer (BD Biosciences). The gating hierarchy was defined as follows: primary lymphocyte/monocyte populations were identified by FSC/SSC profiles; singlets were isolated by FSC-H vs. FSC-W gating; and viable cells were identified as DAPI⁻. Specific subsets, including CD3⁺ T cells, CD19⁺ B cells, and CD14⁺ monocytes, were isolated and collected into tubes pre-filled with RPMI 1640 medium supplemented with 20% FBS. Post-sort purity was consistently verified to be > 95%.

### Analysis of apoptosis

Apoptosis was quantified by flow cytometry using either an Annexin V-FITC/PI kit (Vazyme, #A211-02) or Annexin V-APC (Elabscience, #E-CK-A117) and DAPI staining. Cells were seeded in 12-well plates (2 × 10⁵ cells/well), cultured overnight, and then treated with 0.1% DMSO (control), 1.0 µM Z2-A-Z2, 5 mM NAC, or 2 µM JSH-23 for 24 h. Cells were harvested, washed with PBS, and stained according to the manufacturer’s protocol. Samples were analyzed on a BD FACS Canto II flow cytometer (BD Biosciences), and data were processed using FlowJo software.

### Measurement of mitochondrial membrane potential (ΔΨm)

Cells were seeded and treated as described for the apoptosis assay. After treatment, cells were harvested and stained with 200 µL of JC-1 working solution (1:250 dilution) for 30 min at 37 °C in the dark. The shift in fluorescence, indicating changes in ΔΨm, was analyzed by flow cytometry using FITC and PE channels.

### Cell cycle analysis

Cells were seeded in 6-well plates (4 × 10⁵ cells/well) and treated with 0.1% DMSO or 1.0 µM Z2-A-Z2 for 24 h. Harvested cells were fixed in 70% ethanol at -20 °C overnight. The fixed cells were then washed and stained using a Cell Cycle Detection Kit (Beyotime, #C1052). DNA content was analyzed by flow cytometry, and cell cycle distribution was quantified using appropriate software.

### Measurement of cellular and mitochondrial ROS

For total cellular ROS, treated cells (4 × 10⁵ cells/well) were incubated with 200 µL of H₂DCFH-DA probe (1:1000 dilution) for 30 min at 37 °C. For mitochondrial superoxide, treated cells (2 × 10⁵ cells/well) were stained with MitoSOX™ Red probe. For lipid peroxidation, cells were stained with 5 µM BODIPY-C11 (Abclonal, #RM02821). In all assays, fluorescence was quantified using the appropriate channels on a flow cytometer. For imaging, cells stained with MitoSOX™ Red, Hoechst 33,342, and MitoTracker Green were fixed, mounted onto slides, and imaged using a Zeiss LSM880 confocal microscope.

### Transmission electron microscopy (TEM)

Treated cells were fixed in 2.5% glutaraldehyde, post-fixed in 1% osmium tetroxide, and dehydrated through a graded ethanol series. Samples were embedded in epoxy resin, from which ultrathin Sects.  (60–80 nm) were prepared. Sections were stained with uranyl acetate and lead citrate and examined under a transmission electron microscope to assess ultrastructural changes, particularly in mitochondria and nuclear morphology.

### Quantification of ATP, T-AOC, and T-SOD

Cells were seeded in 6-well plates, treated for 24 h, and then harvested. For ATP measurement, cell lysates were analyzed using an ATP Chemiluminescence Assay Kit (Elabscience, #E-BC-F002). For total antioxidant capacity (T-AOC) and total superoxide dismutase (T-SOD) activity, cell supernatants were prepared by ultrasonication and centrifugation. T-AOC and T-SOD levels were measured using FRAP and WST-1 based kits, respectively (Elabscience, #E-BC-K225-M, #E-BC-K020-M), according to the manufacturer’s protocols. All results were normalized to total protein concentration as determined by a BCA assay (Beyotime, #P0010).

### Molecular docking

To elucidate the potential binding mode of Z2-A-Z2 with the p65 subunit of NF-κB, molecular docking simulations were performed. The three-dimensional crystal structure of human p65 was obtained from the Protein Data Bank (PDB ID: 6QHL). The protein was prepared for docking by removing all water molecules and non-essential ligands; polar hydrogen atoms and appropriate charges were subsequently added using Pymol. The 3D structure of the Z2-A-Z2 ligand was generated and energy-minimized using the UFF force field to obtain its most stable conformation. Docking was carried out using AutoDock Vina, with the search grid centered on the putative binding pocket of p65. The resulting docked conformations were ranked based on their binding affinity scores. The lowest energy binding pose was selected for detailed analysis of intermolecular interactions, including hydrogen bonds and hydrophobic contacts, which were visualized using PyMOL.

### Bioinformatic analysis of public datasets

The differential expression of key NF-κB pathway genes was investigated using data from The Cancer Genome Atlas (TCGA) and the Genotype-Tissue Expression (GTEx) project. Transcriptomic data for NFKBIA (encoding IκBα), NFKB1 (encoding p50), and RELA (encoding p65) from DLBCL tumor samples (*n* = 47) and normal control tissues (*n* = 337) were analyzed and visualized using the Gene Expression Profiling Interactive Analysis 2 (GEPIA2) online tool. Gene expression levels, presented as log₂ (TPM + 1), were compared between the two groups, and statistical significance was determined using the platform’s standard analysis parameters.

### Western blot analysis

Approximately 2 × 10⁶ treated cells were lysed in SDS-containing buffer. Protein concentrations were determined by BCA assay. Equal amounts of protein (15–30 µg) per sample were separated by SDS-PAGE and transferred to a PVDF membrane. The membrane was blocked with 5% non-fat milk for 1 h, incubated with primary antibodies overnight at 4 °C, followed by incubation with HRP-conjugated secondary antibodies for 1 h at room temperature. Protein bands were visualized with an ECL reagent, and densitometry analysis was performed using ImageJ software.

### Quantitative RT-PCR (qRT-PCR)

Total RNA was extracted from treated cells using an RNAeasy™ Kit (Beyotime, #C0026). cDNA was synthesized using the PrimeScript RT Reagent Kit (Biopmk, #PMK2008). qPCR was performed on a CFX96 Real-Time PCR System (Bio-Rad) using 2× SYBR Green qPCR Mix (Biopmk, #PMK2007). Gene expression was normalized to *GAPDH* or *ACTIN*, and relative expression was calculated using the 2⁻ΔΔCt method. The sequences of the primers used are as follows (5’-3’): IκBα-F: ACCTGGTGTCACTCCTGTTGA, IκBα-R: CTGCTGCTGTATCCGGGTG; p65-F: GTGGGGACTACGACCTGAATG, p65-R: GGGGCACGATTGTCAAAGATG; p50-F: GGTGCGGCTCATGTTTACAG, p50-R: GATGGCGTCTGATACCACGG.

### Lentiviral production and p65 stable overexpression

Lentiviral particles were generated by co-transfecting HEK293T cells with a GFP-tagged p65 overexpression vector (MiaoLing, #P42070) alongside the packaging plasmids pVSVG (Addgene, #8454) and pPAX2 (Addgene, #12260). At 48 h post-transfection, the viral supernatants were harvested, filtered, and subsequently employed to transduce SU-DHL-4 and SU-DHL-6 cells. To establish stable cell lines, GFP-positive populations were isolated via fluorescence-activated cell sorting (FACS). The efficiency of p65 overexpression in the sorted cells was further validated by Western blot analysis.

### Transient siRNA transfection

DLBCL cells were seeded into 6-well plates at a density of 5–10 × 10⁵/well in 2 mL of antibiotic-free medium. Transient knockdown of p65 was performed using Lipofectamine 3000 (Thermo Fisher Scientific, #L3000075) according to an optimized spinoculation protocol. Briefly, 5 µL of Lipofectamine 3000 and 2 µL of 100 µM p65 siRNA were each diluted in 125 µL of Opti-MEM™ I Reduced Serum Medium (Gibco, #31985062). After a 5-min incubation, the diluted siRNA and Lipofectamine were combined and incubated for an additional 20 min to allow complex formation. The resulting 250 µL mixture was added dropwise to each well, followed by immediate centrifugation at 1,000 × g for 2 h at room temperature to enhance transfection efficiency.

Following a 12 h incubation, the transfection medium was replaced with fresh complete medium containing antibiotics. Cells were treated with Z2-A-Z2 at 48 h post-transfection and subsequently harvested at 72 h for flow cytometric and Western blot analyses. The p65-targeting siRNA sequences were as follows: si-p65-1, sense 5′-CACCAUACAUCAAUGAUGAGUUTT-3′ and antisense 5′-AACUCAUCAUGAUGUAUGGUGTT-3′; si-p65-2, sense 5′-CCUGAGGCUAUAAACUCGCCUATT-3′ and antisense 5′-UAGGGCAGUUAUAGCCCUCAGGTT-3′; and si-p65-3, sense 5′-GCAGCUAUCAGUCAGCGCAUTT-3′ and antisense 5′-AUGCGCUGACUGAUAGCUGCTT-3′.

### Immunofluorescence

For immunofluorescence analysis, cells were adhered to coverslips, fixed with 4% paraformaldehyde for 15 min at room temperature, and permeabilized with 0.3% Triton X-100 in PBS for 10 min. Following a 1 h blocking step with 3% bovine serum albumin (BSA) to minimize non-specific binding, the cells were incubated overnight at 4 °C with a primary antibody targeting p65 (1:50 dilution). After three washes with PBS, cells were incubated with an Alexa Fluor 488-conjugated secondary antibody (1:500 dilution) for 2 h at room temperature in the dark. Nuclei were counterstained with DAPI (1 µg/mL) for 10 min. Finally, the coverslips were mounted using an anti-fade mounting medium, and fluorescence images were captured using a Zeiss LSM 880 confocal microscope equipped with appropriate filter sets.

### mRNA sequencing and GO enrichment analysis

RNA sequencing was performed by a commercial service provider. Total RNA was extracted from SU-DHL-4 cells treated with DMSO or Z2-A-Z2. Differentially expressed genes were identified using thresholds of |log2FC| > 1 and adjusted p-value < 0.05. Gene Ontology (GO) enrichment analysis was subsequently performed, with terms considered significant at *p* < 0.05.

### In vivo xenograft model

All animal procedures were approved by the Institutional Animal Care and Use Committee (IACUC). To establish GCB- and ABC-derived DLBCL xenograft models, five-week-old male BALB/cA-nu mice (Huafukang, Beijing, China) were subcutaneously inoculated with 1 × 10^7^ SU-DHL-6 or U2932 cells, respectively.When tumors became palpable (day 7), mice were randomized into two groups (*n* = 6/group): vehicle control and Z2-A-Z2 treatment. Mice in the treatment group received daily intraperitoneal injections of Z2-A-Z2 (3.5 mg/kg) for 21 days. Tumor volume and body weight were monitored every three days. At the end of the experiment, mice were euthanized, and tumors and major organs were harvested, weighed, and fixed in 4% paraformaldehyde for subsequent histological analysis.

### Flow cytometric characterization of murine B cell populations

Single-cell suspensions were prepared from the peripheral blood (PB), bone marrow (BM), and spleen. To eliminate erythrocytic contamination, samples were treated with ACK lysing buffer (ThermoFish/Cat#A1049291), followed by two washes in PBS supplemented with 2% FBS. For immunophenotyping, cells were resuspended in chilled staining buffer and labeled with PE-conjugated anti-mouse B220 (clone RA3-6B2; BioLegend, #103208) and APC-conjugated anti-mouse IgM (clone RMM-1; BioLegend, #406509) for 30 min at 4 °C, protected from light. After the final wash, the cells were resuspended in staining buffer and subjected to multi-color flow cytometric analysis using a BD LSRFortessa™. Data were analyzed using FlowJo v10 software.

### Histology and immunohistochemistry

Tumor tissues harvested from the cell-derived xenograft (CDX) model were fixed in 4% paraformaldehyde, processed, and embedded in paraffin blocks. The blocks were sectioned into 4-µm thick slices for all subsequent histological analyses.

For morphological evaluation, tissue sections were deparaffinized, rehydrated, and stained with Hematoxylin and Eosin (H&E) according to standard protocols. For immunohistochemistry (IHC), deparaffinized sections underwent heat-induced epitope retrieval in a citrate buffer (pH 6.0). Endogenous peroxidase activity was quenched with a 3% hydrogen peroxide solution. After blocking non-specific binding, the sections were incubated overnight at 4 °C with the following primary antibodies: IκBα, p50, p65, Ki-67, Cleaved Caspase-3, HMGB1, KIM-1, and NGAL. Following primary antibody incubation, sections were treated with a horseradish peroxidase-conjugated secondary antibody. The signal was visualized using a diaminobenzidine substrate kit, which produces a brown precipitate. Finally, the sections were counterstained with hematoxylin, dehydrated, and mounted. Stained slides were imaged using a light microscope for analysis.

### Statistical analysis

All quantitative data are presented as the mean ± standard deviation (SD) from at least three independent experiments. Statistical analyses were performed using GraphPad Prism 8.0. Unpaired Student’s t-tests were used for comparisons between two groups, while one-way ANOVA followed by an appropriate post-hoc test was used for multiple group comparisons. A P-value < 0.05 was considered statistically significant (*p* < 0.05, *p < 0.01, **p < 0.001, ***p < 0.0001).

## Results

### Synthesis and verification of Z2-A-Z2

**Fig. 1:**
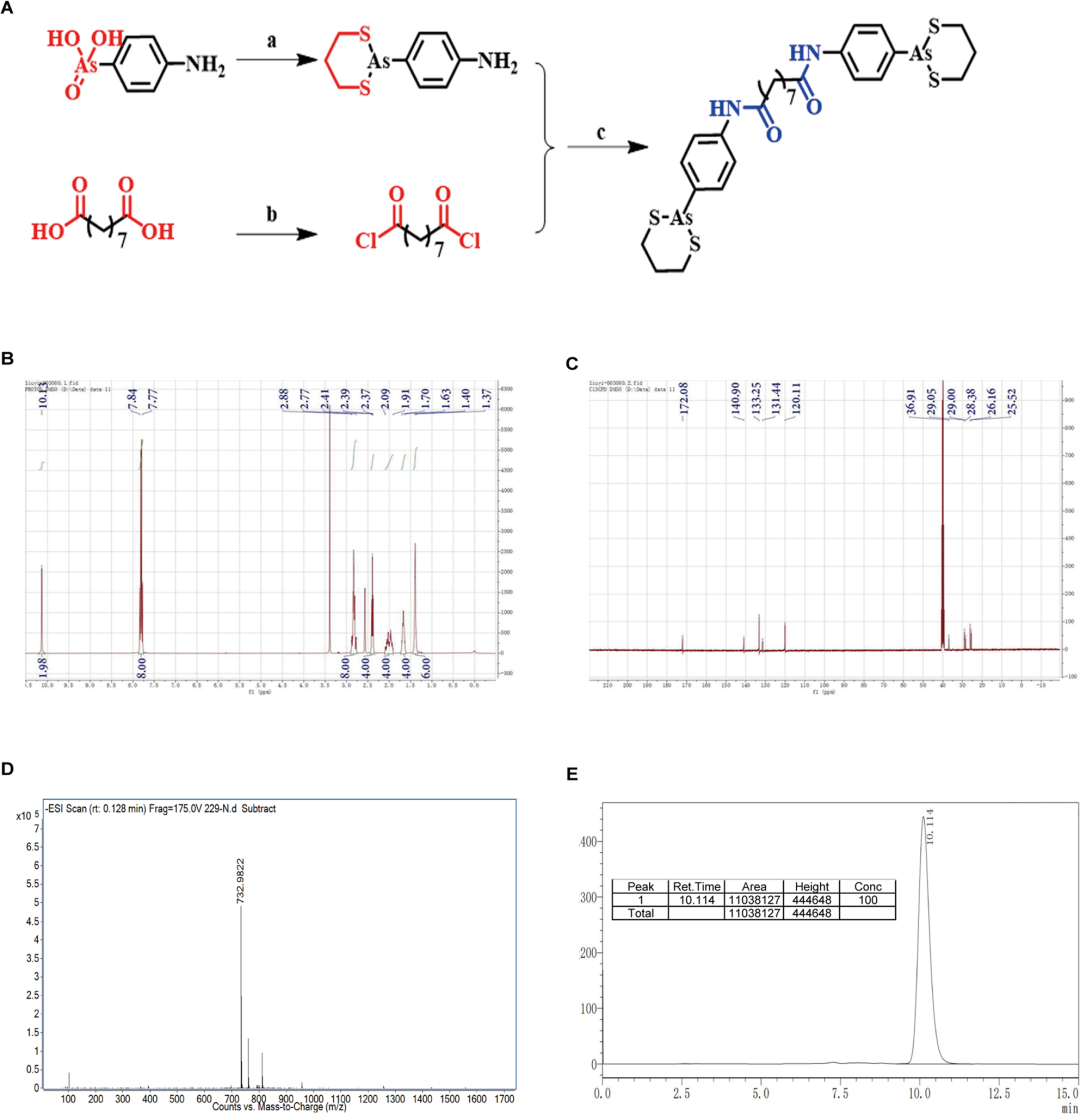
Synthesis and structure of the novel organic arsenical Z2-A-Z2. **A** Reaction scheme for the synthesis of Z2-A-Z2. **B**,** C** ¹H and ¹³C NMR spectra confirming the chemical structure of Z2-A-Z2. **D** Mass spectrum of Z2-A-Z2. **E** Purity of Z2 -A-Z2 determined by high-performance liquid chromatography (HPLC)

The target compound Z2-A-Z2 was synthesized by conjugating two equivalents of 4-(1,3,2-dithiarsinan-2-yl)aniline (from step a) with the azelaic acid derivative (from step b), forming a novel, symmetrical sulfur/arsenic-containing compound (Fig. 1A). The chemical structure of Z2-A-Z2 was fully characterized and verified using ¹H and ¹³C NMR spectroscopy (Fig. 1B-C). The m/c peak 732.9822 in the mass spectrum corresponded to deprotonated molecular ion [M-H]- (Fig. 1D). The purity of Z2-A-Z2 was determined to be 100.0% by high-performance liquid chromatography (Fig. 1E).

### Z2-A-Z2 potently and selectively inhibits DLBCL cell proliferation

**Fig. 2:**
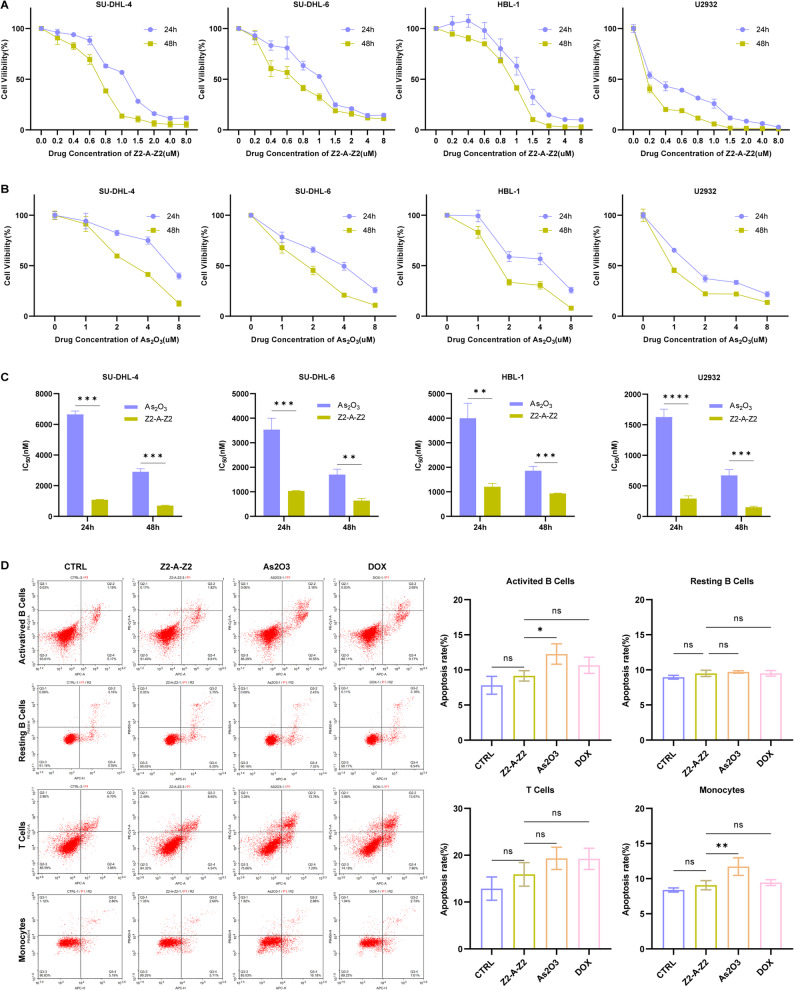
Z2-A-Z2 exerts potent and selective anti-proliferative activity against DLBCL cells. **A**, **B** Dose- and time-dependent growth inhibition of GCB-subtype (SU-DHL-4, SU-DHL-6) and ABC-subtype (U2932, HBL-1) DLBCL cell lines. Cells were treated with the indicated concentrations of Z2-A-Z2 (**A**) or As₂O₃ (**B**) for 24, and 48 h, and cell viability was quantified by CCK-8 assay. **C** Comparison of IC₅₀ values for Z2-A-Z2 and As₂O₃ across the indicated DLBCL cell lines after 24 h of treatment, highlighting the superior potency of the organic arsenical. **D**, **E** Evaluation of the safety profile of Z2-A-Z2. Viability of primary human B cells, T cells, and monocytes isolated from healthy donors was assessed following 24-h exposure to 1 µM Z2-A-Z2 or clinical comparator drugs. Data are presented as mean ± SD from three independent experiments (*n* = 3)

We first evaluated the anti-proliferative activity of Z2-A-Z2 against a panel of DLBCL cell lines. Z2-A-Z2 significantly suppressed the viability of both GCB- (SU-DHL-4, SU-DHL-6) and ABC-subtype (HBL-1, U2932) cells in a dose- and time-dependent manner (Fig. 2A). For comparison, the conventional chemotherapeutic agent arsenic trioxide (As₂O₃) also inhibited cell growth under the same conditions (Fig. 2B). However, Z2-A-Z2 demonstrated markedly superior potency, with 24-hour IC₅₀ values ranging from 293 to 1209 nM, representing a 3- to 6-fold greater efficacy than As₂O₃ (IC₅₀ values: 1620 to 6647 nM) (Fig. 2C). Crucially, Z2-A-Z2 exhibited a superior safety profile, characterized by markedly lower cytotoxicity toward healthy peripheral blood mononuclear cells (PBMCs), primary B cells, and renal cells compared to both As₂O₃ and doxorubicin (Fig. 2D, S1A).

### Z2-A-Z2 induces mitochondrial-mediated apoptosis and G2/M cell cycle arrest

**Fig. 3:**
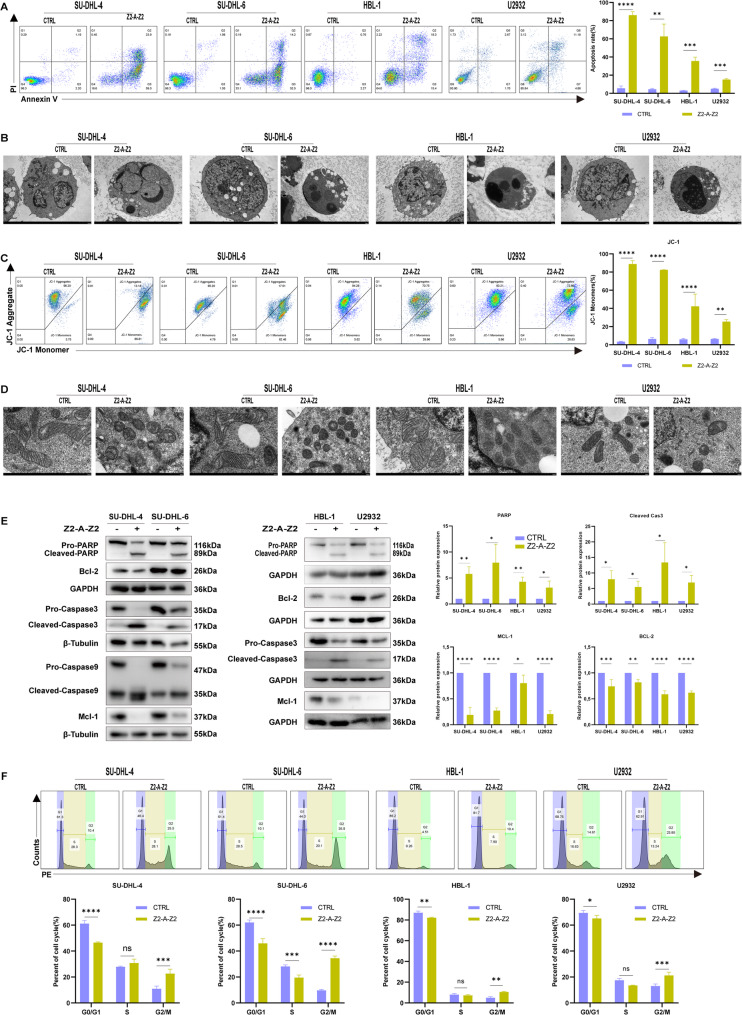
Z2-A-Z2 triggers mitochondrial-mediated apoptosis and G2/M phase arrest in DLBCL cells. **A** Quantification of apoptosis in SU-DHL-4, SU-DHL-6, HBL-1 (treated with 1 µM Z2-A-Z2) and U2932 cells (treated with 0.3 µM Z2-A-Z2) following 24-h treatment, as assessed by Annexin V-FITC/PI dual staining and flow cytometry. **B** Representative transmission electron microscopy (TEM) images displaying hallmark features of apoptosis, including chromatin condensation and nuclear fragmentation. **C** Assessment of mitochondrial membrane potential (ΔΨm) using JC-1 staining; the decrease in the red/green fluorescence intensity ratio indicates mitochondrial depolarization. **D** High-magnification TEM images revealing mitochondrial ultrastructural aberrations, such as cristae remodeling and swelling, post-treatment. **E** Western blot analysis of key apoptotic regulatory proteins, including Bcl-2 family members and cleaved caspases, confirming the activation of the intrinsic apoptotic pathway. **F** Flow cytometric analysis of cell cycle distribution using PI staining, demonstrating a significant accumulation of cells in the G2/M phase. Data are presented as mean ± SD from three independent experiments (*n* = 3)

To elucidate the mechanisms underpinning the anti-proliferative effects of Z2-A-Z2, we first conducted an apoptosis assessment. Flow cytometric analysis via Annexin V/PI dual staining revealed that 24-h treatment with Z2-A-Z2 (1 µM for SU-DHL-4/6 and HBL-1; 0.3 µM for U2932) induced a robust increase in both early and late apoptotic cell populations (Fig. 3A). This finding was further corroborated by transmission electron microscopy (TEM), which revealed hallmark apoptotic morphological changes, including distinct chromatin condensation and nuclear marginalization within treated cells (Fig. 3B).

Given the pivotal role of mitochondrial dysfunction in initiating intrinsic apoptosis, we next investigated the mitochondrial membrane potential (ΔΨm). Z2-A-Z2 treatment induced a significant loss of ΔΨm, evident as a marked decrease in JC-1 red fluorescence (Fig. 3C), unequivocally indicating mitochondrial depolarization. Complementary TEM imaging provided ultrastructural evidence of mitochondrial damage, characterized by blurred and disordered cristae (Fig. 3D). To confirm the subsequent activation of the downstream apoptotic cascade, Western blot analysis was performed. This revealed that Z2-A-Z2 treatment led to a significant downregulation of anti-apoptotic proteins MCL-1 and BCL-2, concurrently with a marked increase in the cleaved forms of caspase-9, caspase-3, and poly(ADP-ribose) polymerase (PARP) (Fig. 3E). Beyond its apoptosis-inducing capabilities, cell cycle analysis further demonstrated that Z2-A-Z2 remarkably caused a significant cell cycle arrest in the G2/M phase (Fig. 3F), a phenomenon associated with the modulation of key cell cycle regulators (Fig. S1B).

These comprehensive data unequivocally establish that Z2-A-Z2 exerts its potent anti-DLBCL effects by robustly triggering the mitochondrial-mediated intrinsic apoptotic pathway and simultaneously inducing G2/M cell cycle arrest.

### Z2-A-Z2 disrupts mitochondrial redox homeostasis by inducing oxidative stress

**Fig. 4:**
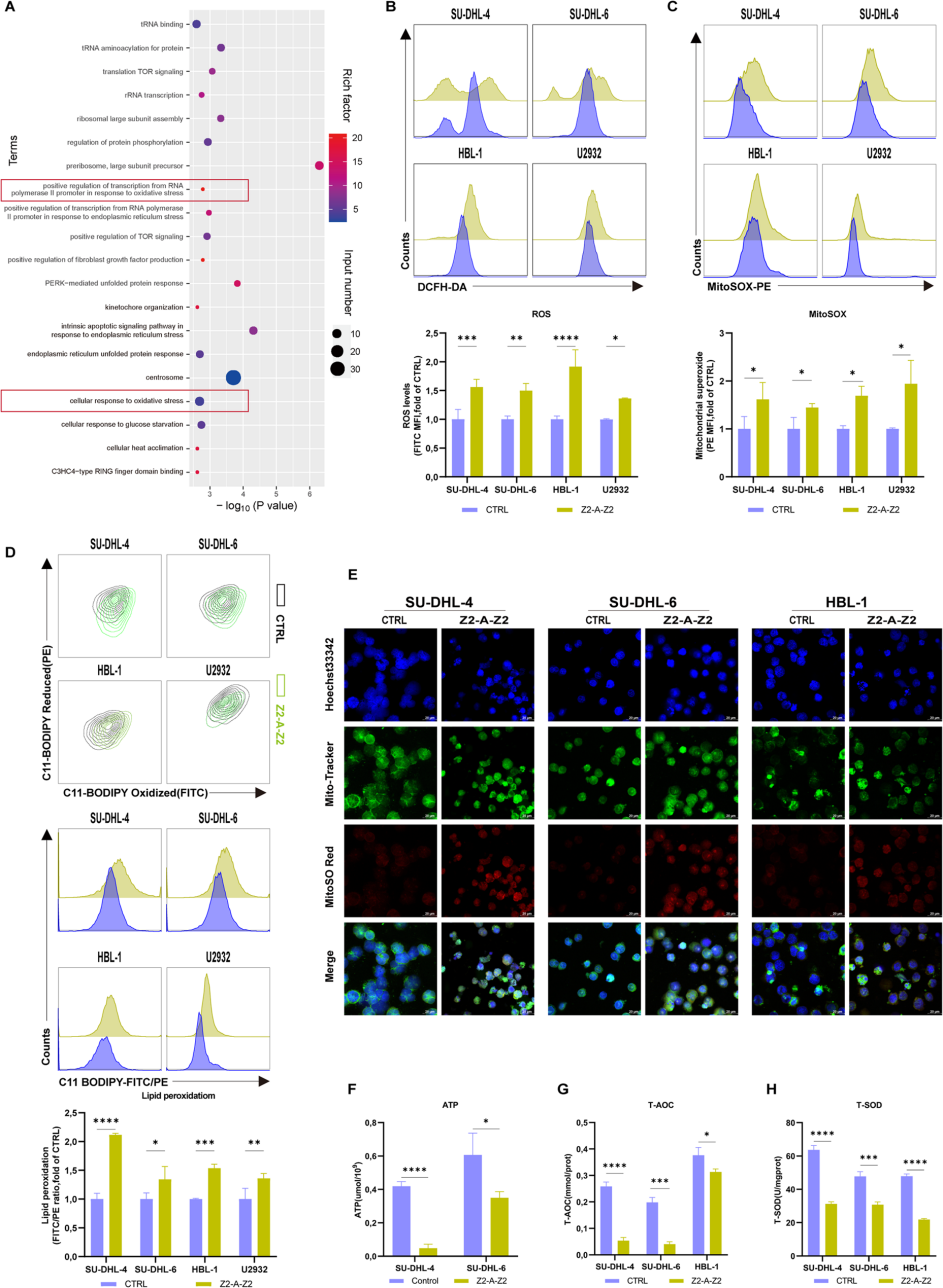
Z2-A-Z2 disrupts redox homeostasis and induces oxidative stress in DLBCL cells. **A** RNA-seq enrichment analysis of SU-DHL-4 cells treated with 1 µM Z2-A-Z2 or 0.1% DMSO (control) for 24 h, presented as a bubble plot. The x-axis indicates the -log_10_ (P value), the y-axis shows selected GO terms, bubble size represents gene count, and color scale denotes the enrichment ratio. **B** Intracellular ROS levels. **C** Lipid peroxidation extent. **D**, **E** Mitochondrial superoxide levels quantified by flow cytometry (**D**) and visualized by confocal microscopy (**E**). **F** Cellular ATP content. **G** Total antioxidant capacity (T-AOC). **H** Total superoxide dismutase (T-SOD) activity. Data are presented as mean ± SD (*n* = 3)

Given the evidence of mitochondrial damage and the significant enrichment of differentially expressed genes in the ‘cellular response to oxidative stress’ via GO analysis (Fig. 4A, S2A), we hypothesized that Z2-A-Z2 fundamentally disrupts cellular redox homeostasis. Our investigations confirmed this, demonstrating a substantial increase in total intracellular reactive oxygen species (ROS), as quantified by the DCFH-DA probe (Fig. 4B). This acute oxidative burden was further substantiated by elevated lipid peroxidation, a key indicator of oxidative damage (Fig. 4D). Delving deeper into the cellular source of ROS, we specifically targeted mitochondrial superoxide using MitoSOX Red staining, which revealed a significant and pronounced overproduction of superoxide within the mitochondria of treated cells (Fig. 4C, E). This catastrophic mitochondrial oxidative stress was mechanistically linked to a critical depletion of cellular ATP levels, signifying profoundly impaired mitochondrial bioenergetics (Fig. 4F). Furthermore, the compound’s effect extended to the cellular antioxidant defense, leading to a significant reduction in both total antioxidant capacity (T-AOC) and total superoxide dismutase (T-SOD) activity, indicating an overwhelmed intrinsic protective system (Fig. 4G-H). In parallel, Z2-A-Z2 treatment initiated certain autophagic events, evidenced by the upregulation of ATG5 and GABARAPL1. However, this process represented an abortive autophagic response with impaired flux, as characterized by the significant accumulation of p62 and the concomitant downregulation of Beclin-1 (Fig. S2B). Notably, the LC3B-II/I ratio exhibited cell line-specific variations, reflecting heterogeneous autophagic processing across different DLBCL contexts. Furthermore, pharmacological blockade of autophagy failed to rescue Z2-A-Z2-induced cytotoxicity (Fig. S2C), suggesting that while autophagic markers are modulated upon treatment, autophagy remains dispensable and is not the primary driver of the anti-DLBCL activity of this organic arsenical.

Collectively, these findings unequivocally establish that Z2-A-Z2 triggers severe and widespread oxidative stress, primarily localized to the mitochondria, which subsequently compromises cellular energy status and induces compensatory autophagic responses.

### Z2-A-Z2 exerts its anti-tumor effects by directly targeting the NF-κB/p65 signaling axis

**Fig. 5:**
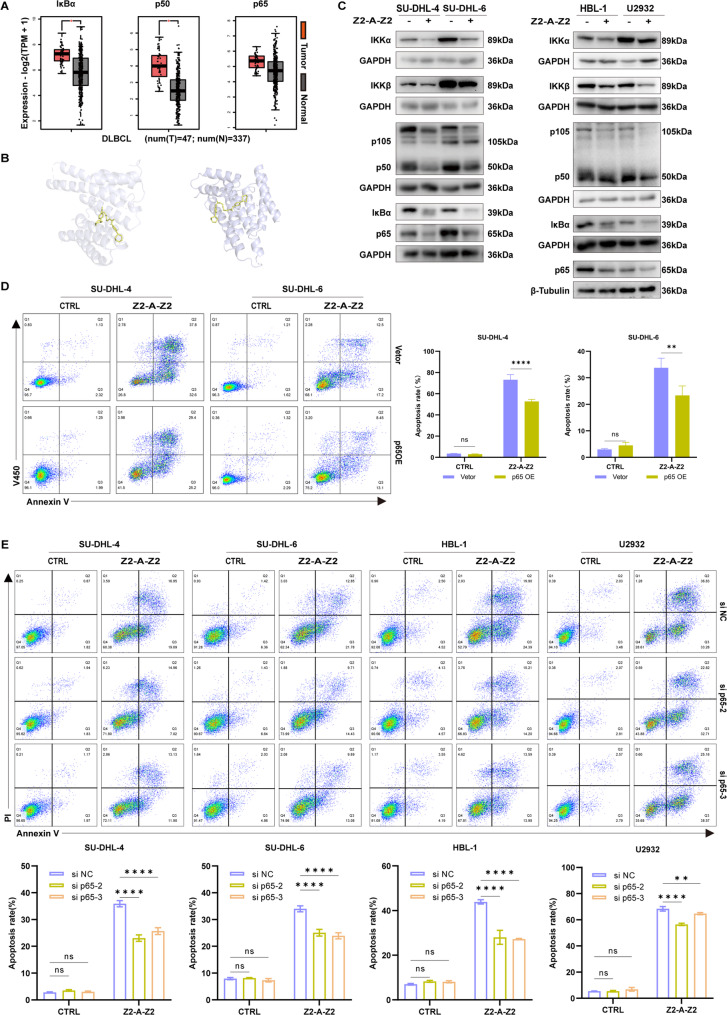
Z2-A-Z2 induces apoptosis by directly targeting and suppressing the NF-κB/p65 signaling axis. **A** Bioinformatic analysis of NF-κB pathway components in DLBCL patient tissues (*n* = 48) compared with normal lymphoid tissues (*n* = 337) based on TCGA and GTEx datasets, revealing consistent oncogenic upregulation. **B** In silico molecular docking simulations illustrating the high-affinity binding of Z2-A-Z2 to the activation loop of the p65 subunit. **C** Western blot analysis demonstrating a downregulation of key NF-κB signaling proteins, including IKKα/β, IκBα, p50, and p65, in SU-DHL-6 and U2932 cells following Z2-A-Z2 treatment. **D** Rescue of the apoptotic phenotype by p65 overexpression. DLBCL cells were transfected with either an empty vector or a p65-overexpression (OE) plasmid prior to Z2-A-Z2 treatment, with apoptosis quantified by flow cytometry. **E** Impact of p65 depletion on Z2-A-Z2-mediated cytotoxicity. Cells were transfected with non-targeting control siRNA (si-NC) or p65-specific siRNA (si-p65) to assess the functional requirement of p65 in drug efficacy. Data are presented as mean ± SD from three independent experiments (*n* = 3)

GO enrichment analysis revealed that differentially expressed genes were predominantly clustered in biological processes associated with the “positive regulation of NF-κB transcription factor activity” and “IκB kinase/NF-κB signaling” (Fig. S2A). Cross-referencing TCGA and GTEx databases corroborated that core components of the NF-κB pathway (IκBα, p50, and p65) were significantly overexpressed in DLBCL patient tissues relative to normal controls (Fig. 5A). Consistent with the known pharmacological profile of arsenicals, target prediction and molecular docking simulations indicated that Z2-A-Z2 directly binds to the activation loop of the p65 subunit (Fig. 5B). Consequently, Z2-A-Z2 treatment induced a significant downregulation of key pathway members, including IKKα, IKKβ, IκBα, p50, and p65, at both transcript and protein levels (Fig. 5C, S3A–B).

To establish the functional requirement of NF-κB inhibition in Z2-A-Z2-mediated cytotoxicity, we genetically and pharmacologically modulated p65 activity. Overexpression of p65 (p65 OE) was confirmed via Western blot (Fig. S3C), which subsequently promoted cell proliferation (Fig. S3D) and upregulated the anti-apoptotic effectors *BCL2* and *XIAP* (Fig. S3E). Crucially, p65 overexpression markedly attenuated Z2-A-Z2-induced apoptosis (Fig. 5D), suggesting that persistent NF-κB activation confers a survival advantage against the compound. Conversely, siRNA-mediated knockdown of p65 reduced the sensitivity of cells to Z2-A-Z2(Fig. 5E), while co-treatment with the p65 nuclear translocation inhibitor JSH-23 synergistically enhanced its anti-proliferative and pro-apoptotic efficacy (FigS3F–H). Collectively, these findings underscore that direct suppression of the NF-κB/p65 signaling axis is a fundamental mechanism by which Z2-A-Z2 triggers apoptosis in DLBCL.

### Reciprocal regulation between ROS and NF-κB/p65 mediates Z2-A-Z2-induced apoptosis

**Fig. 6:**
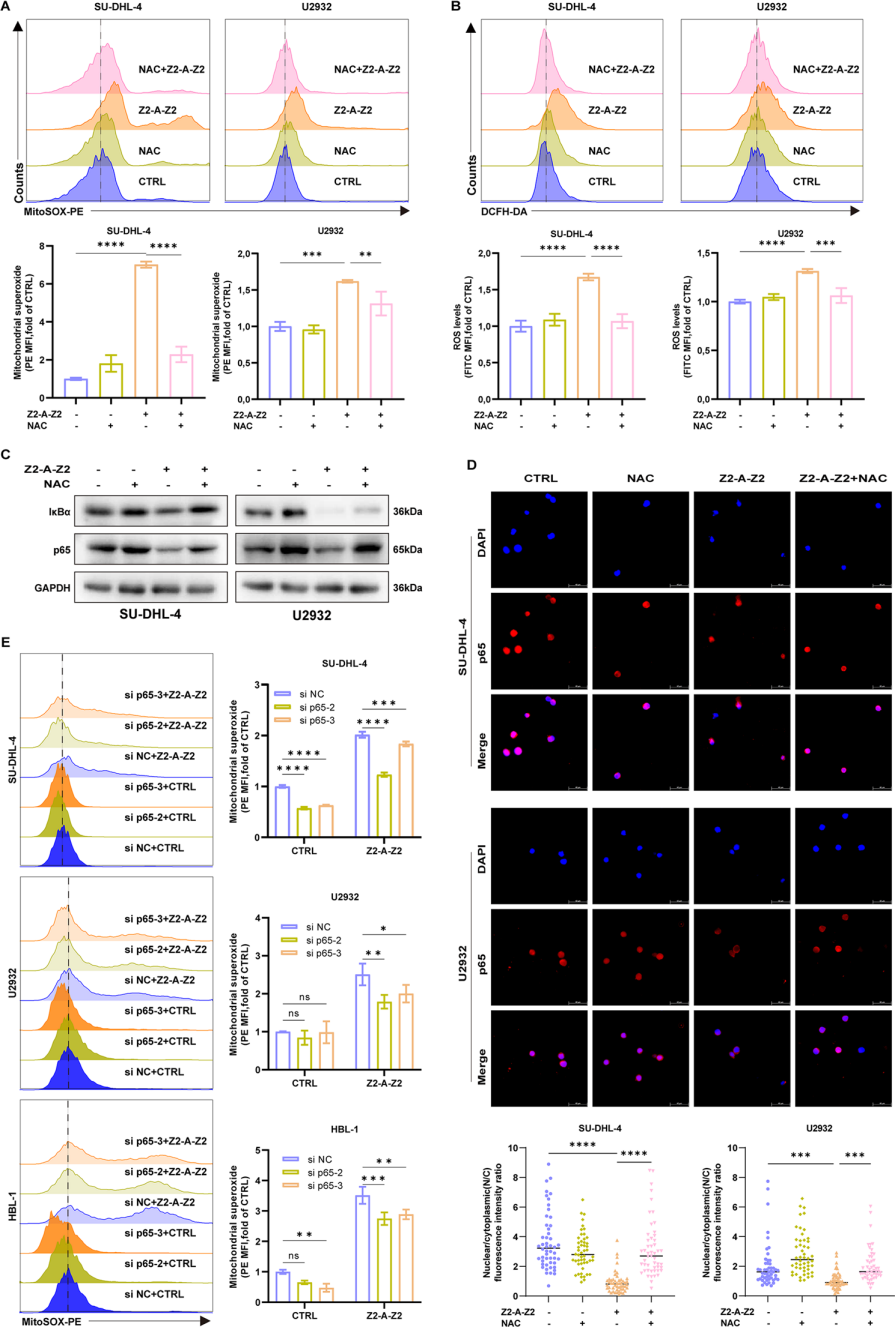
Reciprocal crosstalk between ROS accumulation and NF-κB signaling dictates Z2-A-Z2-induced apoptosis. **A–D** Functional validation of ROS as an upstream regulator of p65. DLBCL cells were pretreated with or without the ROS scavenger N-acetylcysteine (NAC, 5 mM) for 1 h, followed by Z2-A-Z2 exposure for 24 h. **A**, **B** Flow cytometric quantification of mitochondrial superoxide (MitoSOX) (**A**) and total intracellular ROS (DCFH-DA) (**B**), demonstrating the antioxidant efficacy of NAC. **C** Western blot analysis of p65 protein expression showed that ROS scavenging rescues Z2-A-Z2-induced p65 suppression. **D** Representative immunofluorescence images illustrating the inhibitory effect of Z2-A-Z2 on p65 nuclear translocation and its subsequent restoration by NAC pretreatment. **E** Evaluation of p65 as a downstream modulator of oxidative stress. Mitochondrial superoxide levels were quantified in cells transfected with non-targeting control siRNA (si-NC) or p65-specific siRNA (si-p65) following Z2-A-Z2 treatment, highlighting the requirement of p65 for drug-induced ROS propagation. Data are presented as mean ± SD from three independent experiments (*n* = 3)

To confirm that ROS generation was the primary driver of Z2-A-Z2-induced cytotoxicity, rescue experiments were performed using the ROS scavenger N-acetylcysteine (NAC). Pretreatment with NAC for 1 h effectively reversed the Z2-A-Z2-induced elevation of both mitochondrial superoxide and total intracellular ROS levels (Fig. 6A–B, S4A–B). Consequently, NAC pretreatment significantly mitigated the loss of mitochondrial membrane potential and completely abrogated the induction of apoptosis (Fig. S4C–D), establishing ROS as a central mediator of cell death.

To further elucidate the interplay between oxidative stress and NF-κB/p65 signaling, we assessed the impact of ROS inhibition on p65 activity. NAC pretreatment effectively reversed the Z2-A-Z2-induced downregulation of p65 protein expression (Fig. 6C). Moreover, NAC restored p65 nuclear translocation (Fig. 6D) and rescued the inhibitory effect of Z2-A-Z2 on p65 phosphorylation (Fig. S4E), indicating that ROS act upstream to suppress p65 signaling. Conversely, we investigated whether p65 contributes to Z2-A-Z2-induced oxidative stress. siRNA-mediated p65 knockdown not only reduced basal ROS levels but also significantly attenuated Z2-A-Z2-triggered ROS production (Fig. 6E), suggesting that p65 is requisite for drug-induced oxidative stress. Furthermore, activating NF-κB with lipopolysaccharide (LPS) exacerbated Z2-A-Z2-induced ROS accumulation (Fig. S4F), confirming that NF-κB activation potentiates the pro-oxidant effects of the compound. Collectively, these findings reveal a complex bidirectional regulatory axis: while ROS suppress p65 activity, p65 concurrently facilitates ROS generation, creating a feedback loop that enhances Z2-A-Z2-induced oxidative damage.

### Z2-A-Z2 suppresses DLBCL tumor growth in vivo

**Fig. 7:**
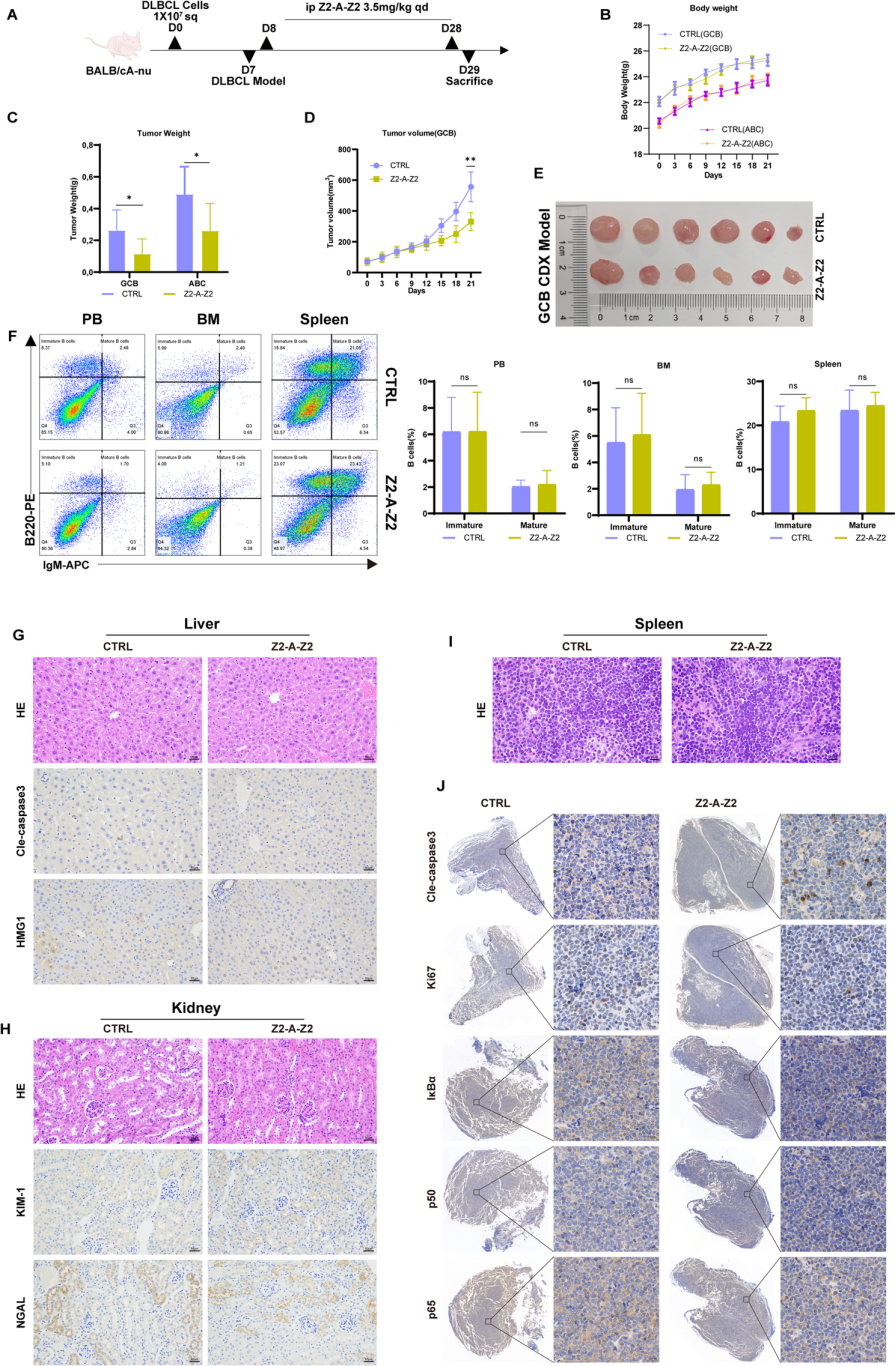
Z2-A-Z2 suppresses DLBCL tumor growth in vivo without inducing systemic toxicity. **A** Schematic representation of the experimental workflow for the DLBCL xenograft mouse model and the Z2-A-Z2 treatment regimen. **B** Longitudinal monitoring of mouse body weights throughout the 21-day treatment period, indicating favorable tolerability. **C–E** Assessment of anti-tumor efficacy at the experimental endpoint. Quantitative analysis of tumor weights (**C**) and volumes (**D**), complemented by representative photographs of resected tumors (**E**), demonstrating robust growth inhibition in the Z2-A-Z2-treated group. **F** Immunophenotypic analysis of the B-cell compartment in the ABC-subtype DLBCL xenograft model. Representative flow cytometry plots and corresponding quantification of B220⁺ B cells among viable lymphocytes in the peripheral blood, bone marrow, and spleen are shown, revealing no significant lymphoid depletion. Data are expressed as mean ± SEM (*n* = 6 per group). **G–I** Safety evaluation via histological analysis in the GCB-subtype DLBCL xenograft model. Representative H&E-stained sections of the liver, kidney, and spleen show no overt histopathological lesions or structural damage. **J** Mechanistic characterization of tumor tissues via immunohistochemistry (IHC) in the ABC-subtype DLBCL xenograft model. Representative images of Ki-67, cleaved caspase-3, and key NF-κB pathway components (p65, p50, and IκBα) are presented, confirming reduced proliferation, increased apoptosis, and suppression of NF-κB signaling in vivo. ata in C, F are presented as mean ± SEM (*n* = 6)

Finally, we rigorously evaluated the in vivo therapeutic efficacy of Z2-A-Z2 using SU-DHL-6 and U2932 cell-derived xenograft (CDX) models established in BALB/cA-nu mice. The experimental schematic is outlined in Fig. 7A. Daily intraperitoneal administration of Z2-A-Z2 (3.5 mg/kg) for 21 days resulted in a profound suppression of tumor progression. Throughout the treatment period, Z2-A-Z2 exhibited excellent tolerability, as evidenced by the absence of significant body weight fluctuations compared to the vehicle group (Fig. 7B). By the experimental endpoint, Z2-A-Z2 treatment exerted a profound anti-tumor effect, evidenced by a 2.5-fold reduction in tumor volume compared to the vehicle group (*P*<0.01); this potent efficacy was further corroborated by a commensurate decrease in tumor weight, highlighting the systemic activity of the compound against ABC-DLBCL xenografts (Fig. 7C–E, S5A-B).

To further assess systemic toxicity in the ABC-subtype CDX model, we analyzed the immune compartment and biochemical parameters. No significant alterations were observed in the percentages of B220⁺ B cells within the peripheral blood, bone marrow, or spleen of Z2-A-Z2-treated mice (Fig. 7F). Furthermore, serum biochemical analysis, including routine hematological counts and hepatic/renal functional assays, showed no significant deviations from the control group (Fig. S5C-E). Consistently, in the GCB-subtype CDX model, histological examination (H&E staining) of major organs, such as the liver, spleen, and kidneys, revealed no overt pathological lesions or treatment-related toxicities (Fig. 7G–I, S5F).

Mechanistic validation was performed via immunohistochemical (IHC) analysis of excised tumor tissues. Z2-A-Z2 treatment led to a significant attenuation of cellular proliferation (indicated by decreased Ki67 staining) and a substantial induction of apoptosis (evidenced by increased cleaved caspase-3 levels). Critically, the expression of key NF-κB pathway components, including IκBα, p50, and p65, was markedly diminished in the Z2-A-Z2-treated group (Fig. 7J, S5G). In summary, these compelling in vivo findings unequivocally demonstrate that Z2-A-Z2 effectively suppresses DLBCL growth by antagonizing the NF-κB signaling axis while maintaining a superior safety profile.

## Discussion

The present study identifies Z2-A-Z2, a novel organic arsenic compound, as a potent anti-lymphoma agent that effectively suppresses DLBCL growth. Our findings elucidate a dual mechanism of action involving the concurrent inhibition of the pro-survival NF-κB signaling pathway and the induction of lethal mitochondrial oxidative stress, demonstrating significant therapeutic potential against this aggressive malignancy.

**Fig. 8:**
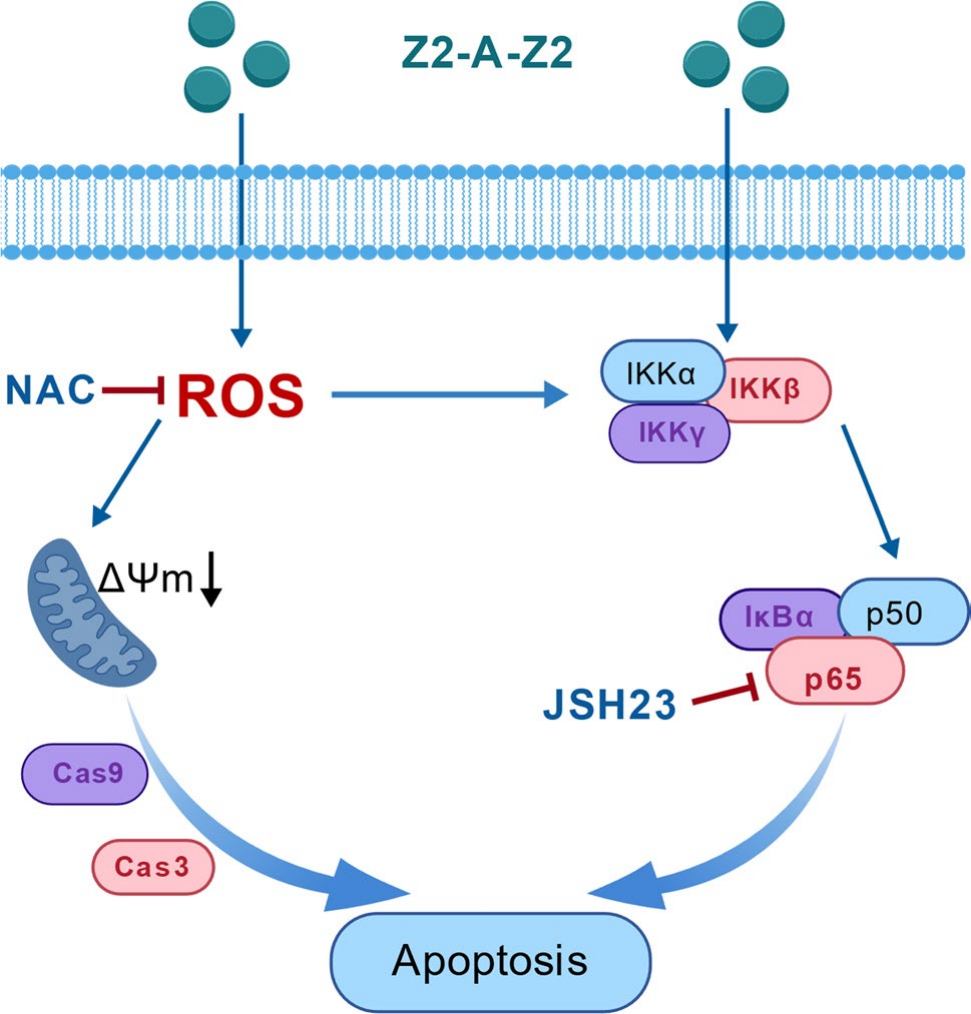
Schematic model illustrating the dual mechanism of Z2-A-Z2-induced apoptosis. Z2-A-Z2 simultaneously induces mitochondrial ROS (mtROS) production and inhibits the NF-κB signaling pathway, leading to the activation of mitochondrial-dependent apoptosis. (Figure created with BioRender.com)

The constitutive activation of the NF-κB pathway is a well-established driver of tumorigenesis and chemoresistance in DLBCL, particularly the ABC subtype, making it a prime therapeutic target [20]. Our results demonstrate that Z2-A-Z2 potently downregulates key components of this pathway, including the central subunit p65. The functional significance of this inhibition was unequivocally confirmed through genetic manipulation; overexpression of p65 markedly rescued DLBCL cells from Z2-A-Z2-induced apoptosis, whereas a p65-specific inhibitor, JSH23, synergized with Z2-A-Z2 to enhance its anti-proliferative effects (Fig. 8). This provides compelling evidence that targeting the NF-κB/p65 axis is a critical component of Z2-A-Z2’s anti-tumor activity.

A central question arising from our findings is how Z2-A-Z2 simultaneously induces massive ROS accumulation while inhibiting the NF-κB pathway, given the complex and often contradictory interplay between these two systems. The relationship between ROS and NF-κB is highly context-dependent, where ROS can act as both an upstream activator and a downstream target of NF-κB signaling [21]. While ROS can, under certain conditions, promote NF-κB activation by inhibiting phosphatases or through alternative kinase pathways, it can also exert a potent inhibitory effect [22, 23]. A key mechanism for this inhibition is the direct oxidation of critical cysteine residues within the NF-κB cascade components [24]. Significantly, the IκB kinase β (IKKβ) subunit contains a highly reactive cysteine residue (Cys-179) in its activation loop, and its oxidation leads to the inactivation of the kinase, thereby blocking the canonical NF-κB pathway [25]. Notably, arsenite, the inorganic precursor of our compound, has been specifically shown to inhibit IKKβ activity precisely through the oxidation of this Cys-179 residue [26]. Therefore, we propose a sophisticated mechanistic model centered on a self-amplifying redox-signaling loop.

In this model, Z2-A-Z2 initially triggers catastrophic oxidative stress, which acts upstream to suppress the NF-κB/p65 axis—potentially through the redox-sensitive thiol modification of IKKβ at Cys-179, consistent with its inorganic arsenical counterparts. Crucially, our data reveal that this regulation is bidirectional: while ROS-mediated inhibition of IKKβ/p65 blunts pro-survival signaling, the residual or initial p65 activity paradoxically facilitates further ROS production, as evidenced by the attenuation of oxidative stress upon p65 knockdown and its exacerbation by LPS. This reciprocal interplay suggests that Z2-A-Z2 exploits a vicious cycle where drug-induced ROS and NF-κB suppression mutually reinforce one another, ultimately driving mitochondrial dysfunction and irrevocable apoptosis in DLBCL cells.

In recent years, arsenic analogues have gained attention in oncology, with arsenic trioxide (ATO) being a notable success in acute promyelocytic leukemia (APL) [27]. However, the broader use of ATO is constrained by significant systemic toxicities [17]. Our data clearly position Z2-A-Z2 as a superior alternative. It exhibited 3- to 6-fold greater potency than ATO against DLBCL cell lines while demonstrating markedly lower cytotoxicity toward healthy PBMCs, B cells and renal cells. This improved therapeutic window was further validated in vivo, where Z2-A-Z2 achieved significant tumor suppression without inducing weight loss or observable histopathological damage in major organs. This enhanced safety and efficacy profile likely stems from structural optimizations unique to this organic arsenical.

While our data establish Z2-A-Z2 as a promising dual-mechanism agent, several limitations merit consideration. First, while Z2-A-Z2 demonstrated excellent efficacy and short-term safety, comprehensive pharmacokinetic studies are required to assess its absorption, distribution, metabolism, and excretion, which are essential for determining its clinical feasibility. Second, a more thorough investigation of potential long-term toxicity is necessary for clinical translation. Finally, while our proposed mechanism of IKKβ oxidation is strongly supported by the literature, direct experimental confirmation using site-directed mutagenesis of Cys-179 would be required to definitively validate this specific molecular interaction.

## Conclusion

In conclusion, our study identifies the novel organic arsenical Z2-A-Z2 as a highly effective agent against DLBCL. We reveal a potent dual therapeutic strategy wherein the compound directly inhibits the NF-κB survival pathway, while simultaneously inducing overwhelming mitochondrial ROS. This synergistic attack on two critical nodes of cancer cell biology provides a strong rationale for the continued development of Z2-A-Z2 as a promising clinical candidate for the treatment of DLBCL.

## Supplementary Information

Supplementary Material 1.

Supplementary Material 2.

Supplementary Material 3.

## Data Availability

No datasets were generated or analysed during the current study.
