# Supplementary figures and images for: Superior anti-DLBCL efficacy of novel organic arsenical Z2-A-Z2 through ROS-mediated apoptosis and critical NF-κB/IκBα signaling pathway inhibition
Source: J Exp Clin Cancer Res. 2026 May 18;45:155. doi: 10.1186/s13046-026-03724-4 (PMC13352683; doi:10.1186/s13046-026-03724-4)

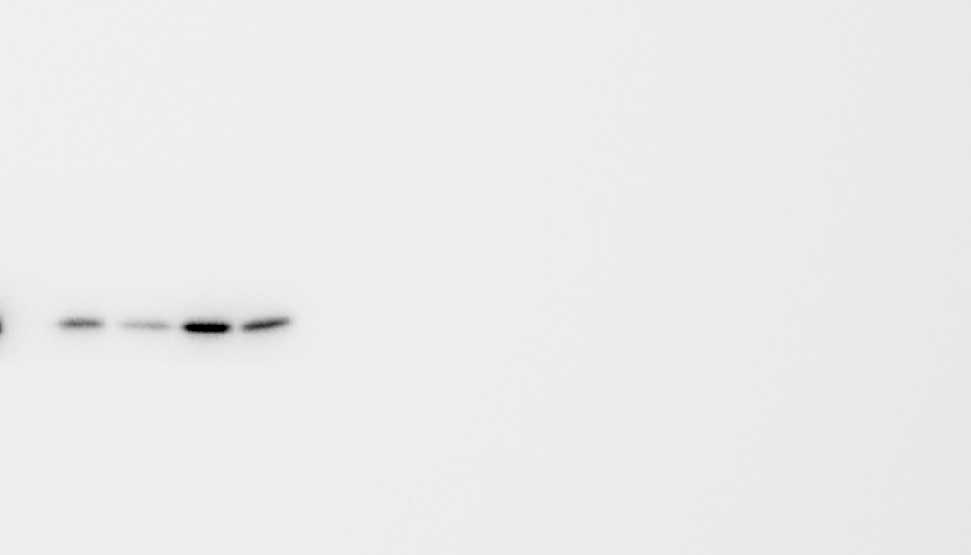

Supplement: Supplementary file 1 — Supplementary Material 1. [file 13046_2026_3724_MOESM1_ESM.zip › WB tiff/ABC-BCL2.jpg]

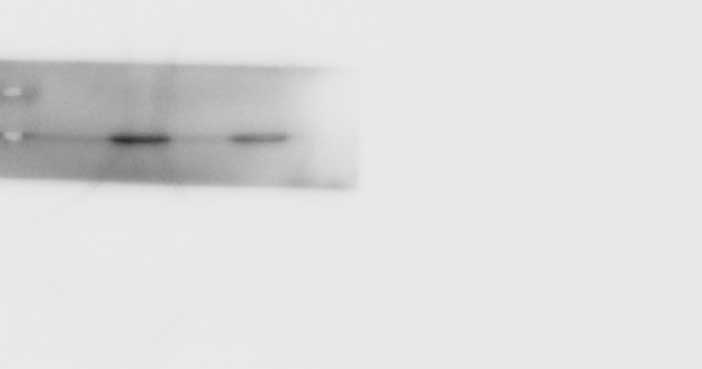

Supplement: Supplementary file 1 — Supplementary Material 1. [file 13046_2026_3724_MOESM1_ESM.zip › WB tiff/ABC-C-C3.jpg]

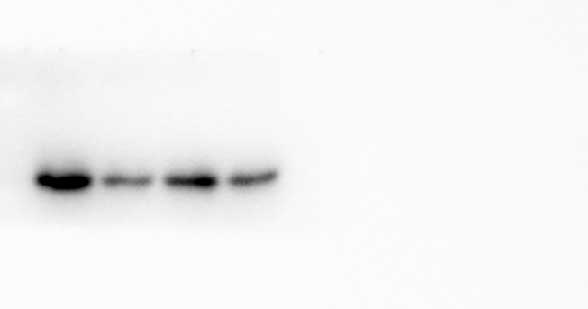

Supplement: Supplementary file 1 — Supplementary Material 1. [file 13046_2026_3724_MOESM1_ESM.zip › WB tiff/ABC-C3.jpg]

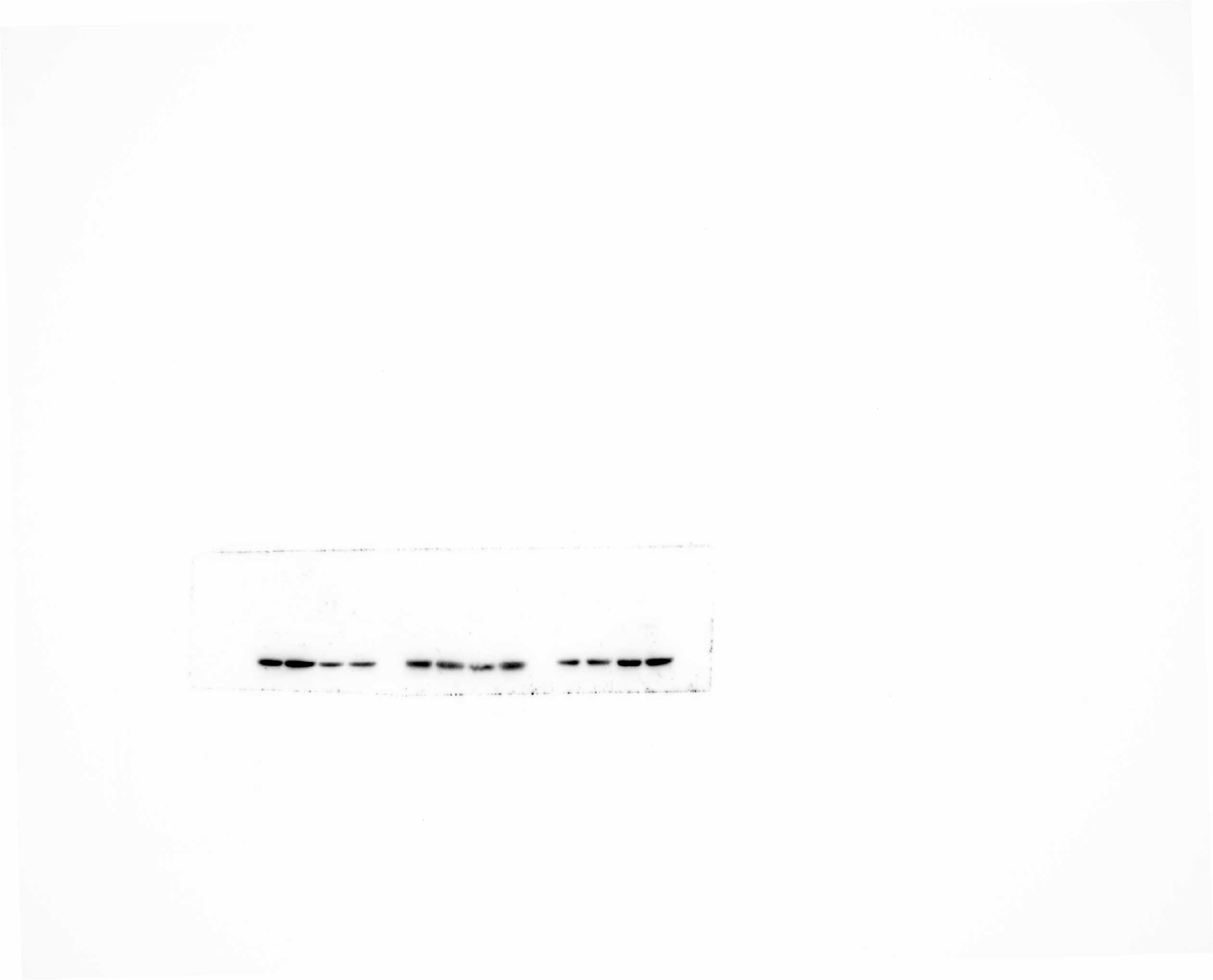

Supplement: Supplementary file 1 — Supplementary Material 1. [file 13046_2026_3724_MOESM1_ESM.zip › WB tiff/ABC-GAPDH-BCL2.jpg]

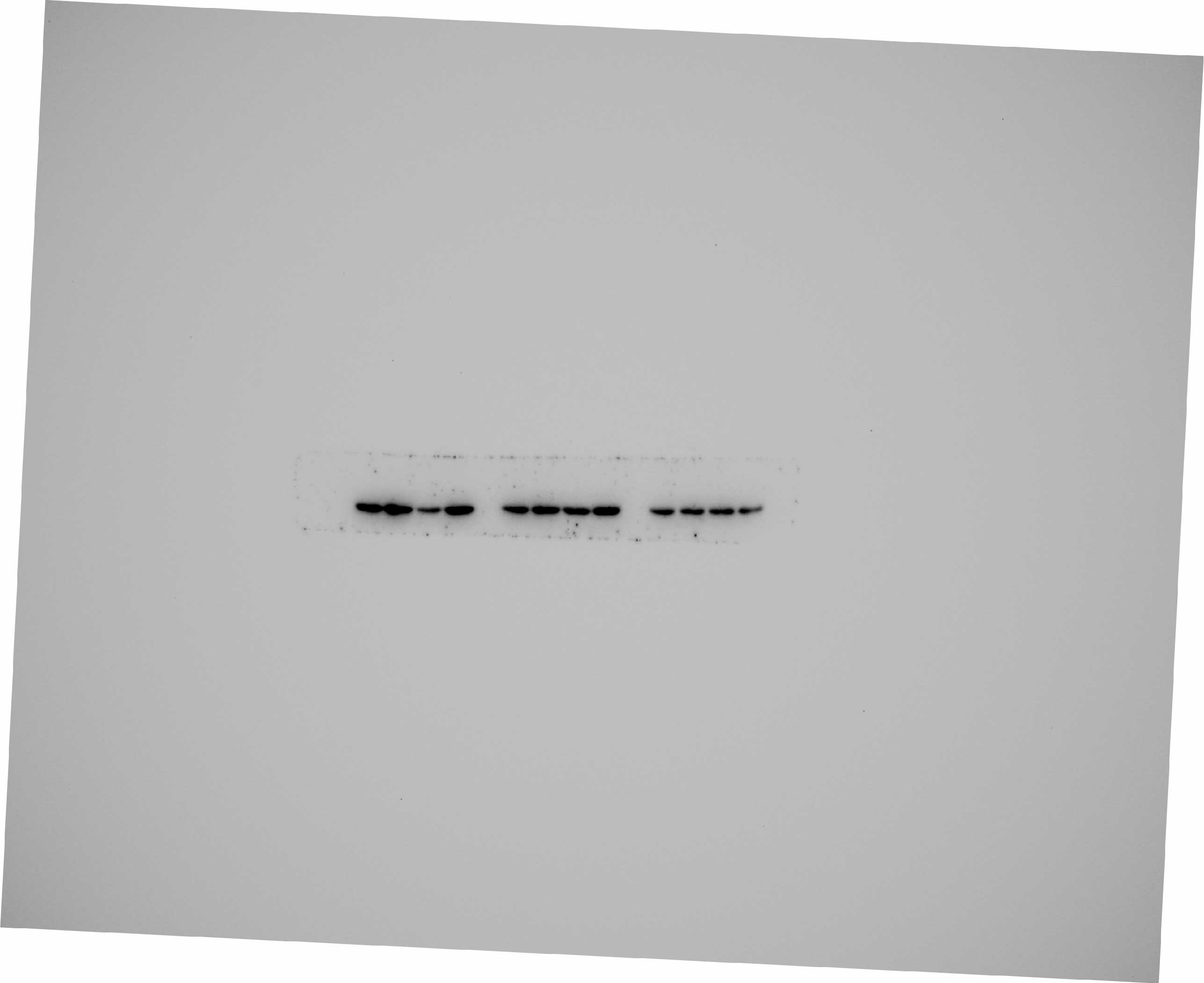

Supplement: Supplementary file 1 — Supplementary Material 1. [file 13046_2026_3724_MOESM1_ESM.zip › WB tiff/ABC-GAPDH-C3.jpg]

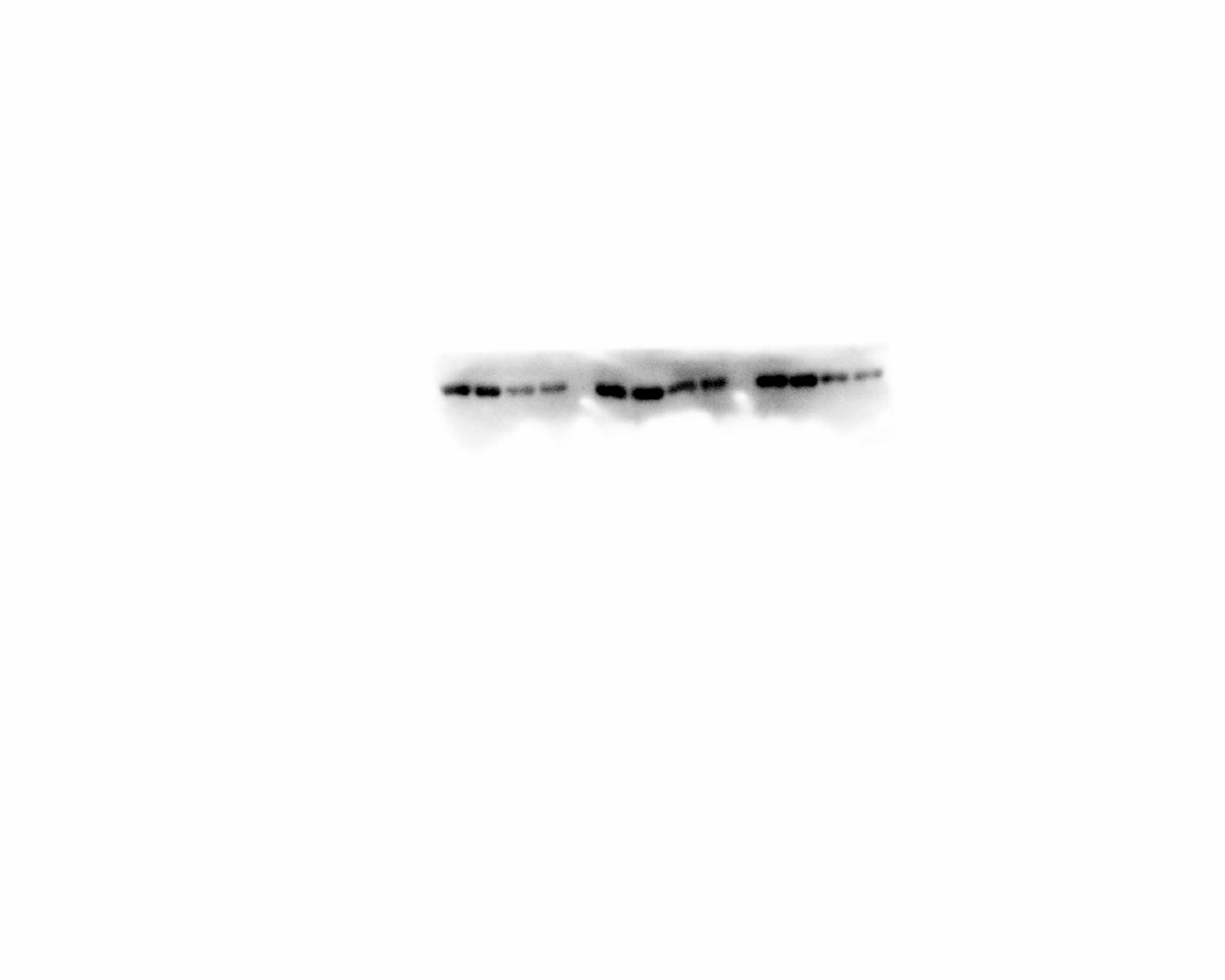

Supplement: Supplementary file 1 — Supplementary Material 1. [file 13046_2026_3724_MOESM1_ESM.zip › WB tiff/ABC-GAPDH-MCL1.jpg]

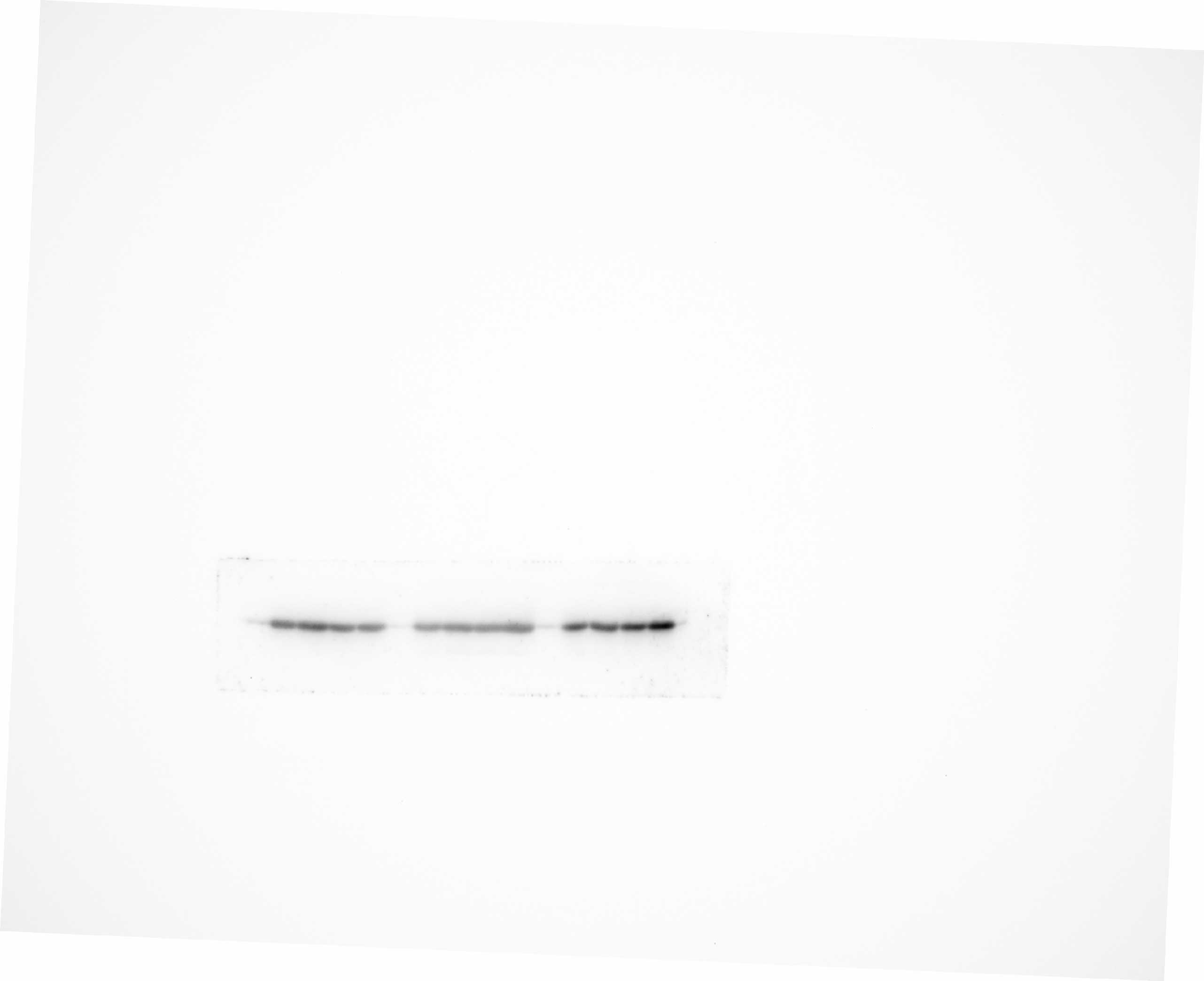

Supplement: Supplementary file 1 — Supplementary Material 1. [file 13046_2026_3724_MOESM1_ESM.zip › WB tiff/ABC-GAPDH-PARP.jpg]

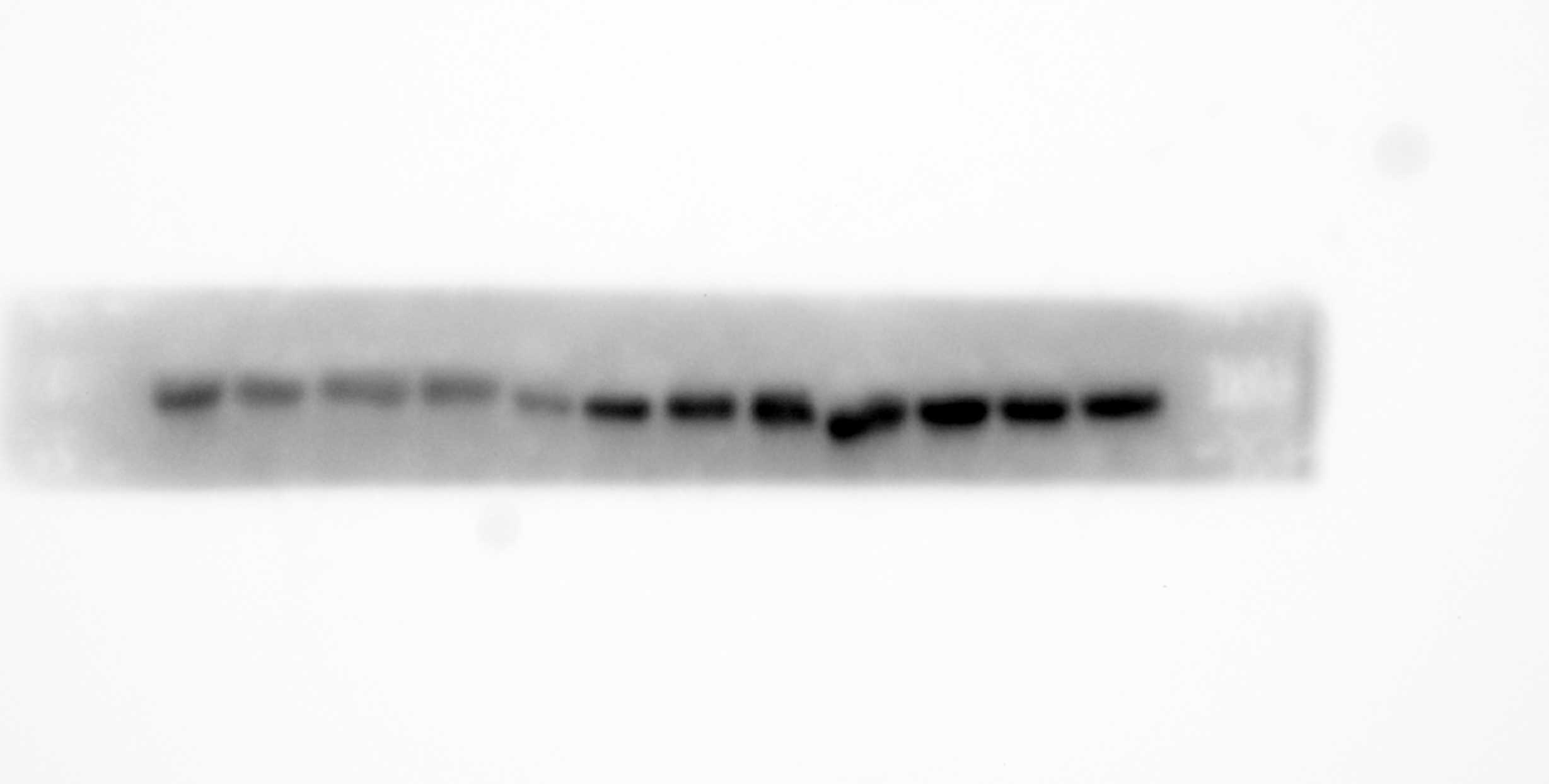

Supplement: Supplementary file 1 — Supplementary Material 1. [file 13046_2026_3724_MOESM1_ESM.zip › WB tiff/ABC-IKBA-GA.jpg]

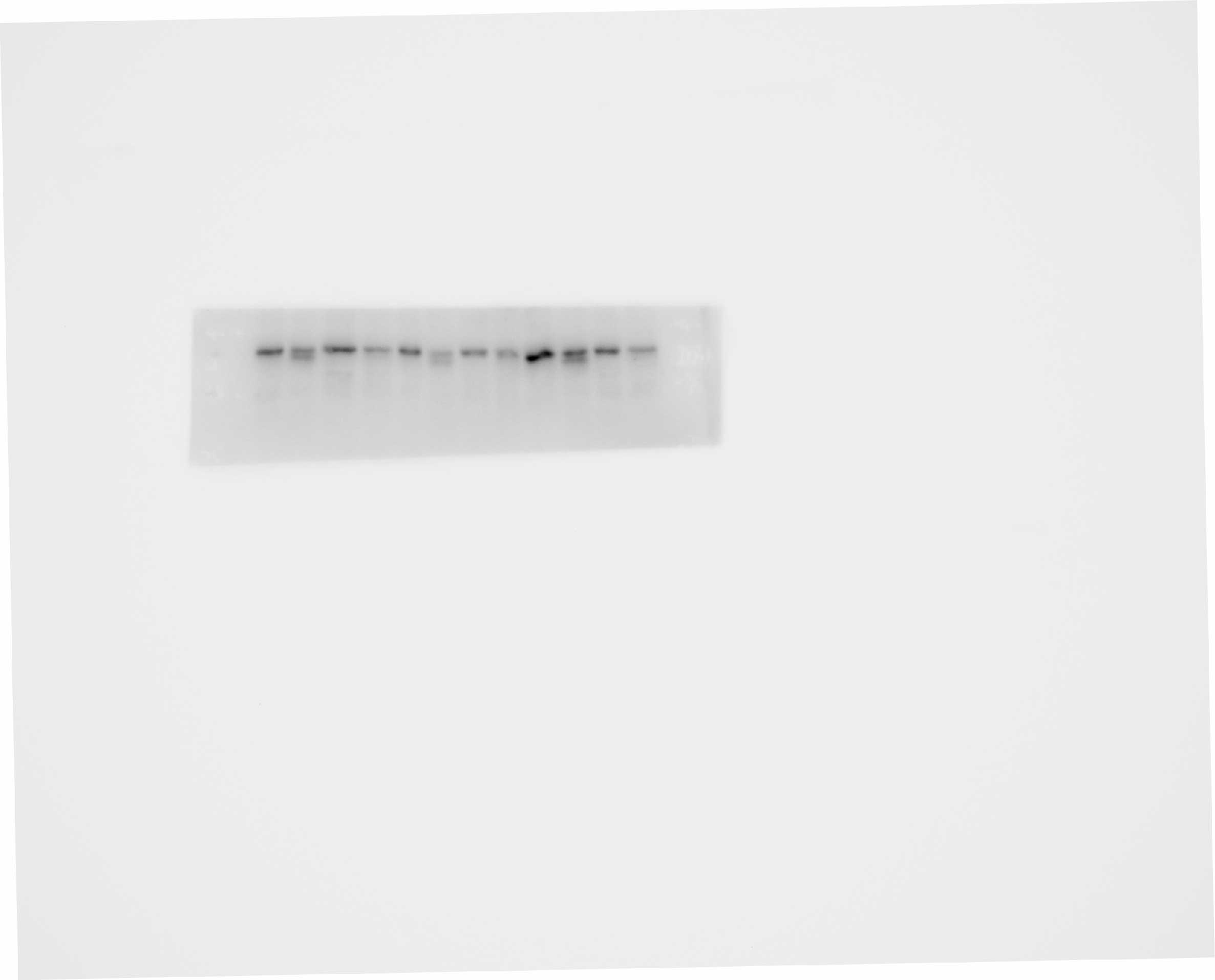

Supplement: Supplementary file 1 — Supplementary Material 1. [file 13046_2026_3724_MOESM1_ESM.zip › WB tiff/ABC-IKBa.jpg]

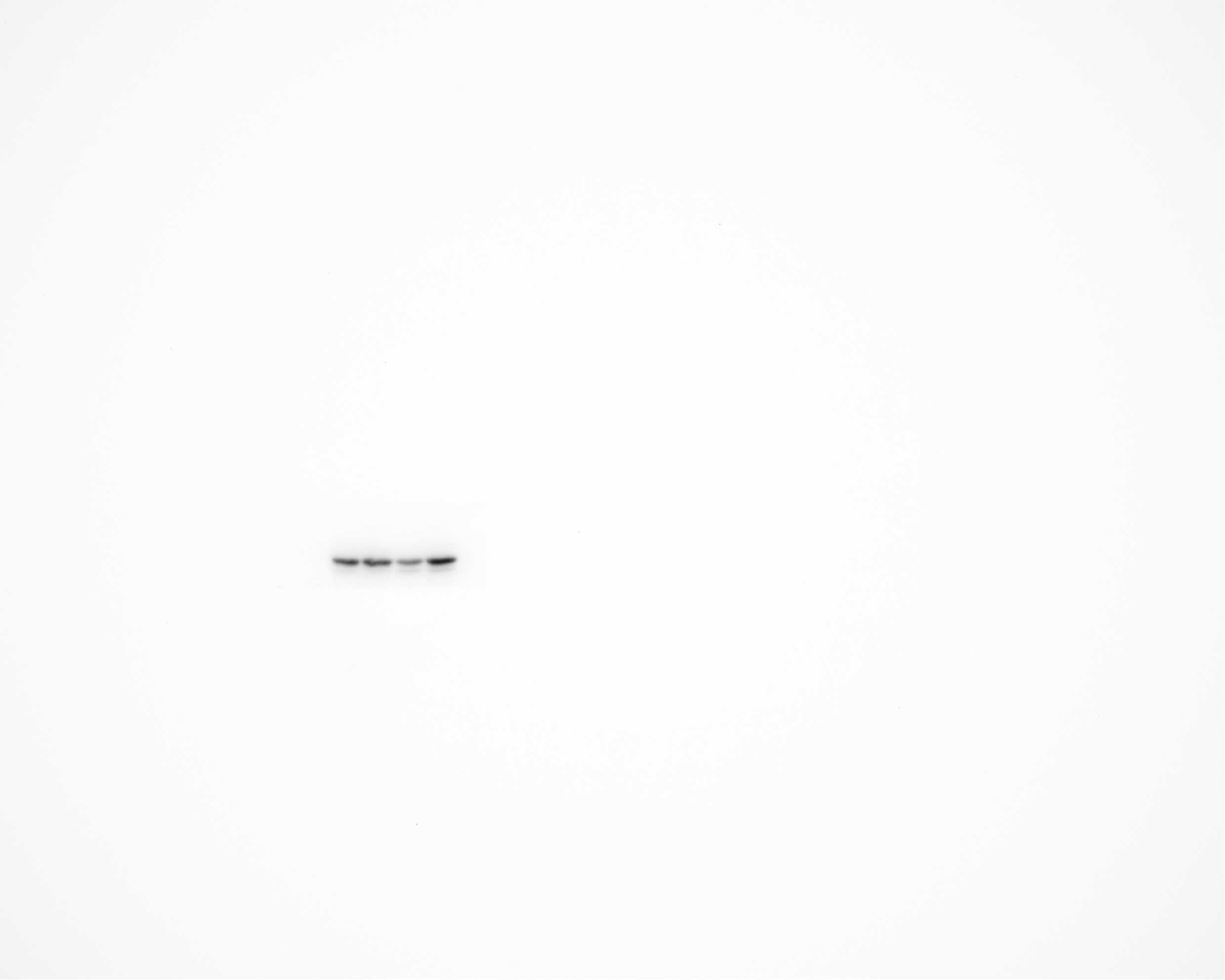

Supplement: Supplementary file 1 — Supplementary Material 1. [file 13046_2026_3724_MOESM1_ESM.zip › WB tiff/ABC-IKKa-GA.jpg]

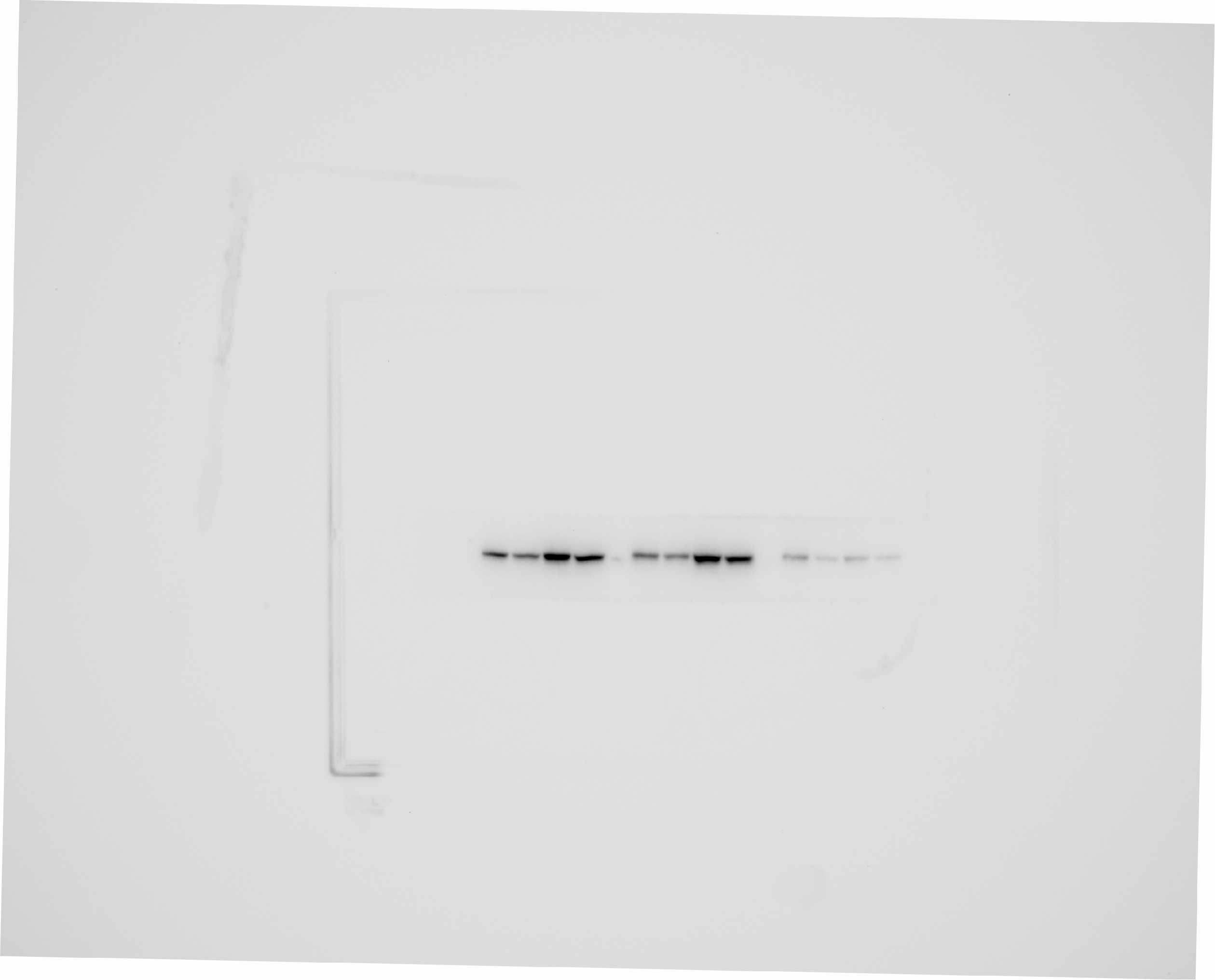

Supplement: Supplementary file 1 — Supplementary Material 1. [file 13046_2026_3724_MOESM1_ESM.zip › WB tiff/ABC-IKKa.jpg]

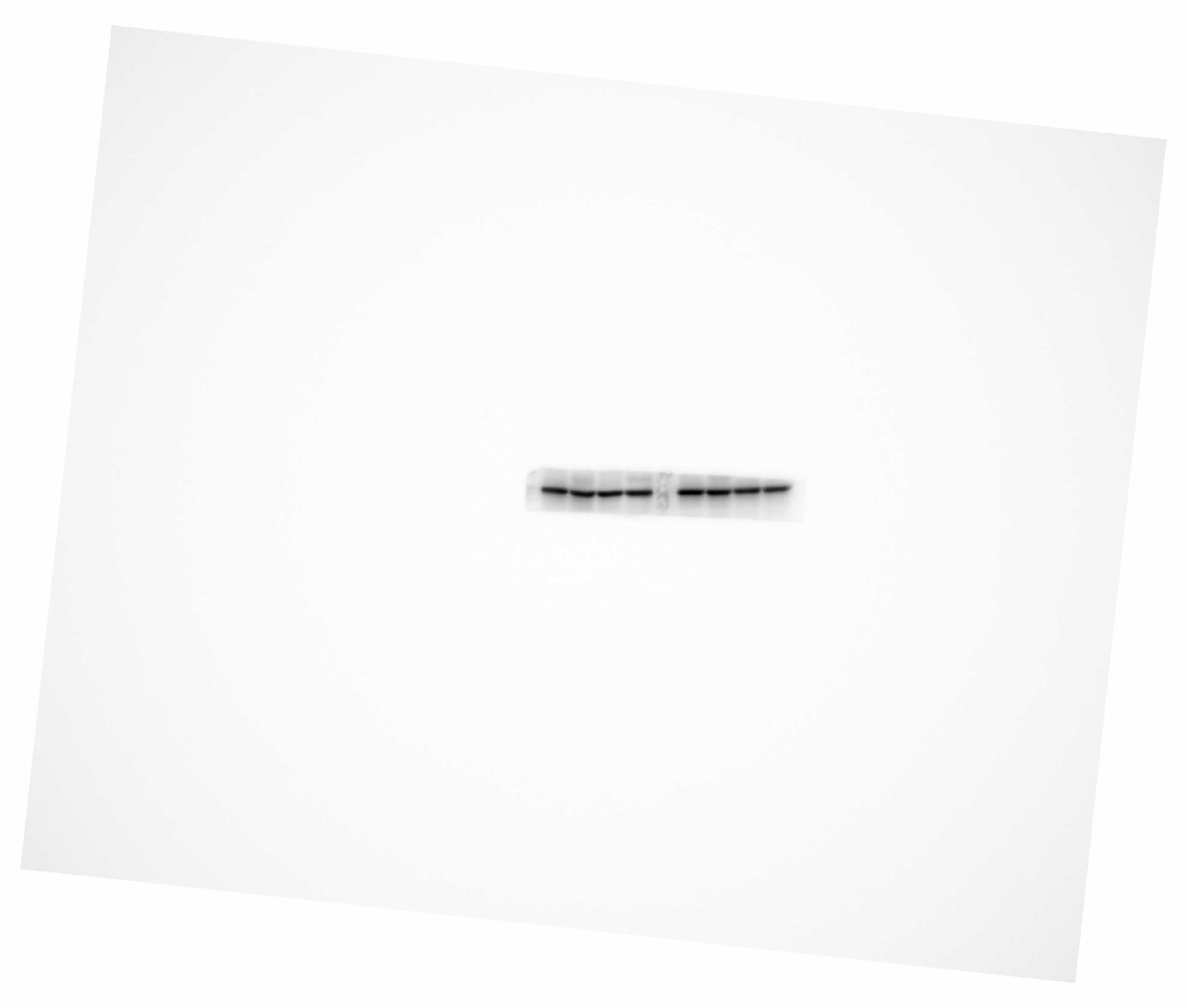

Supplement: Supplementary file 1 — Supplementary Material 1. [file 13046_2026_3724_MOESM1_ESM.zip › WB tiff/ABC-IKKb--Tubulin.jpg]

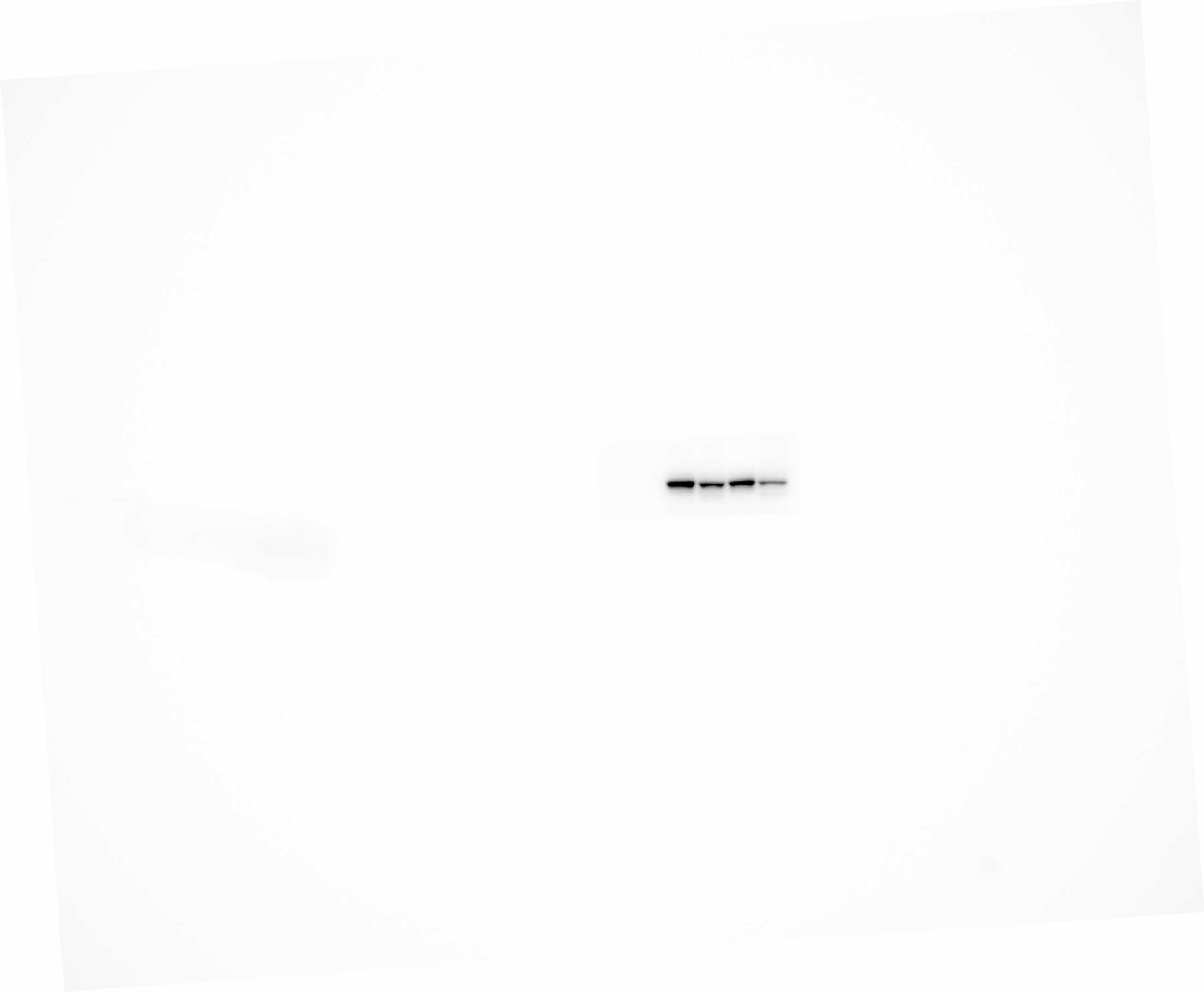

Supplement: Supplementary file 1 — Supplementary Material 1. [file 13046_2026_3724_MOESM1_ESM.zip › WB tiff/ABC-IKKb.jpg]

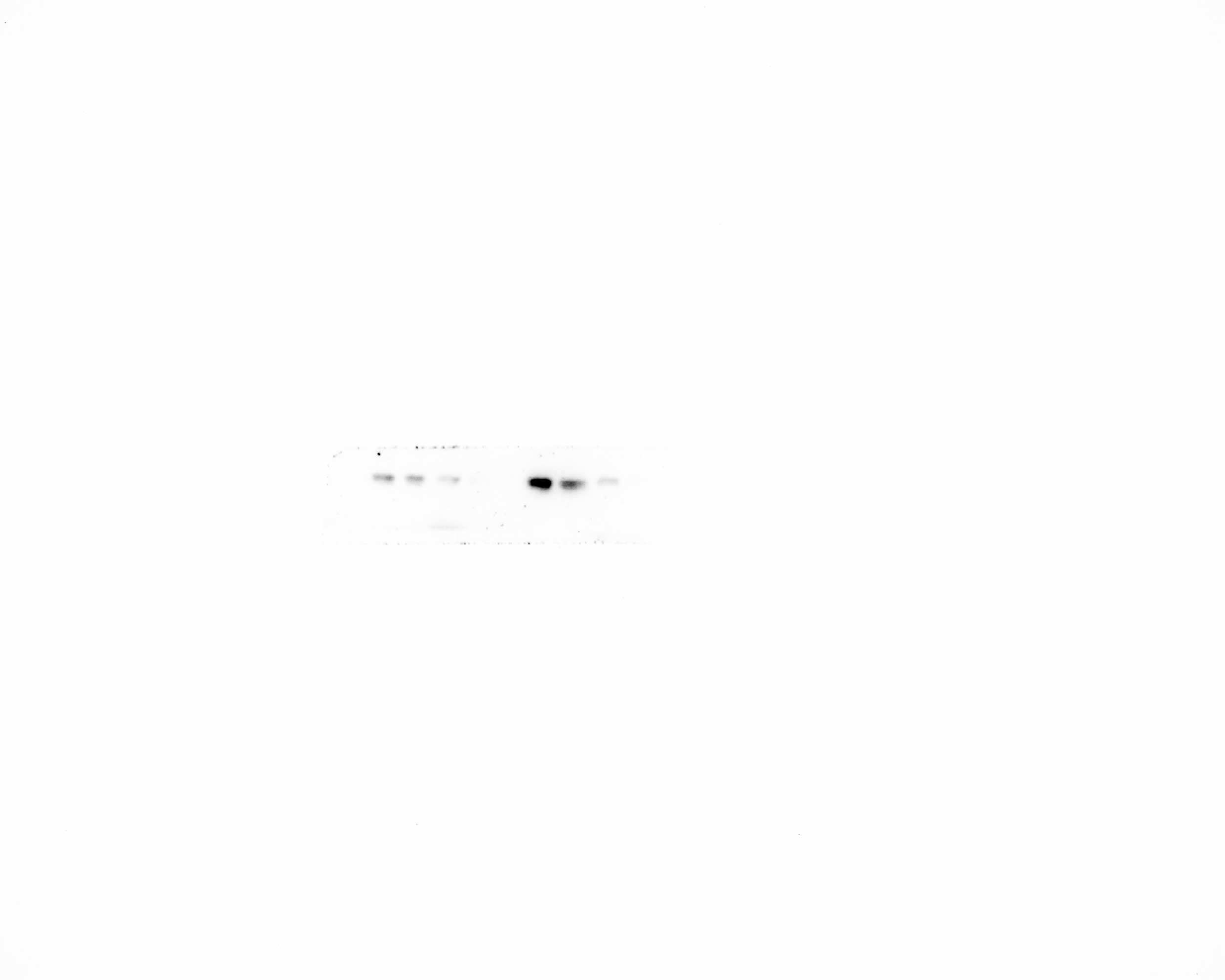

Supplement: Supplementary file 1 — Supplementary Material 1. [file 13046_2026_3724_MOESM1_ESM.zip › WB tiff/ABC-MCL-1.jpg]

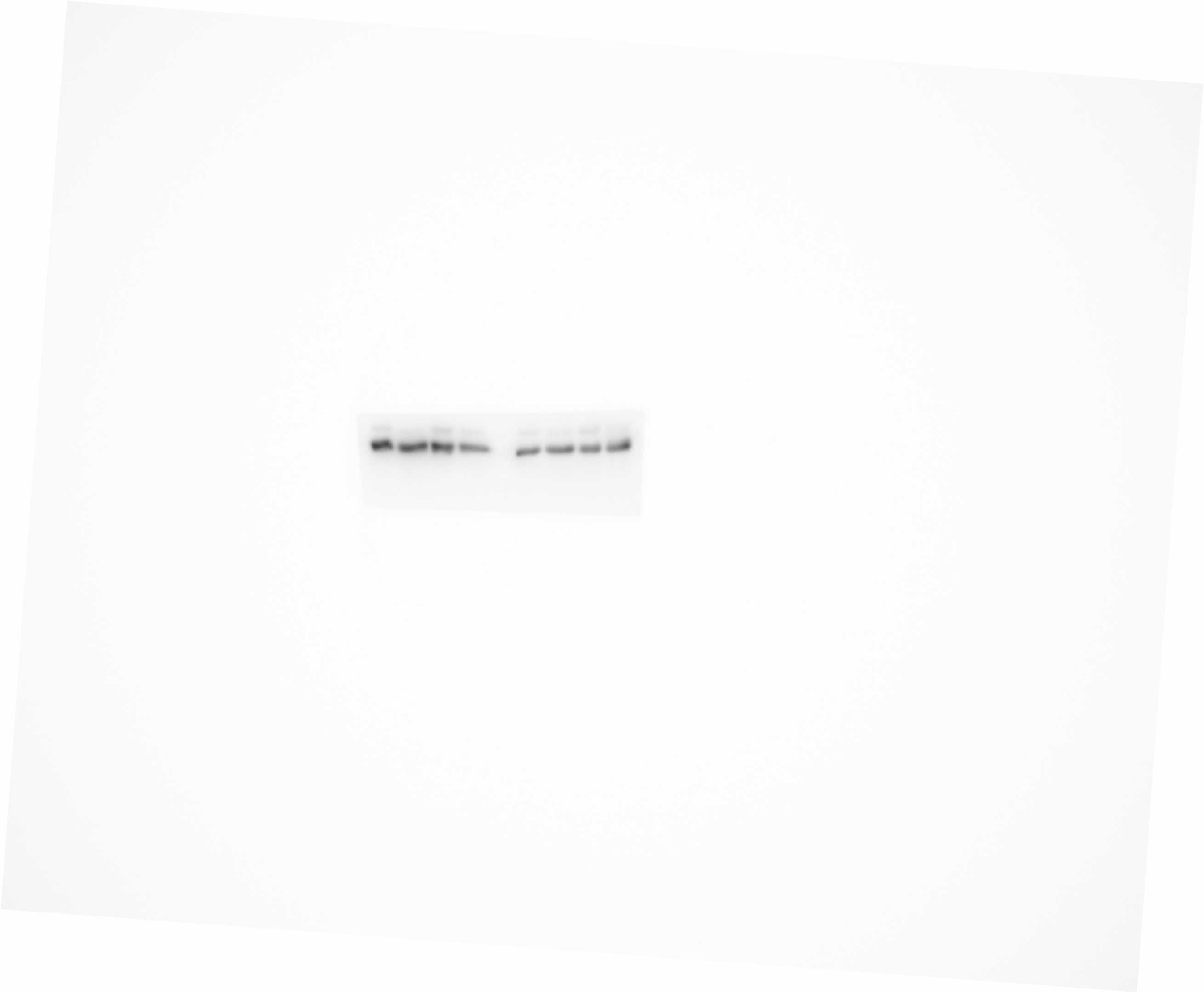

Supplement: Supplementary file 1 — Supplementary Material 1. [file 13046_2026_3724_MOESM1_ESM.zip › WB tiff/ABC-P50-GA.jpg]

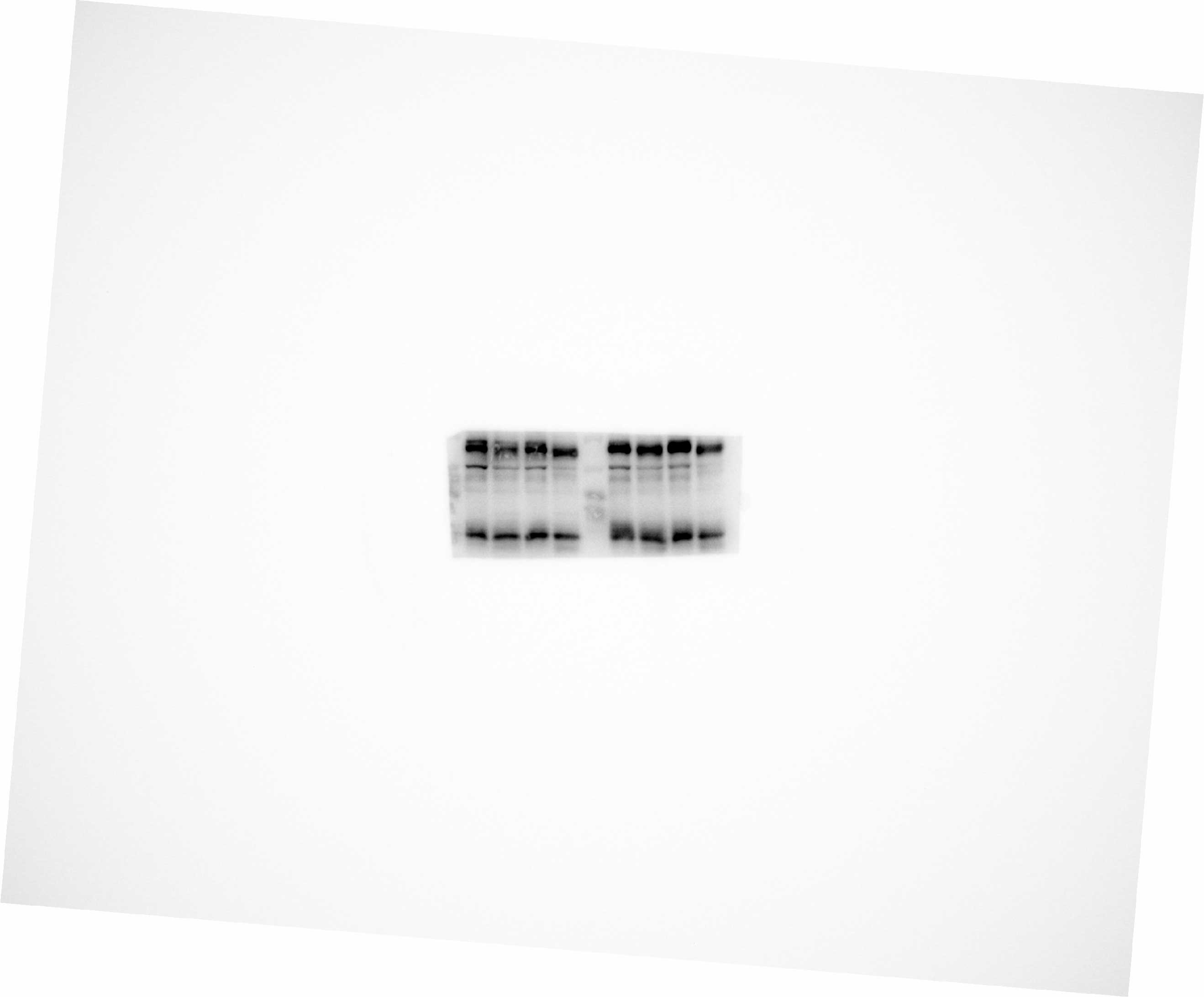

Supplement: Supplementary file 1 — Supplementary Material 1. [file 13046_2026_3724_MOESM1_ESM.zip › WB tiff/ABC-P50.jpg]

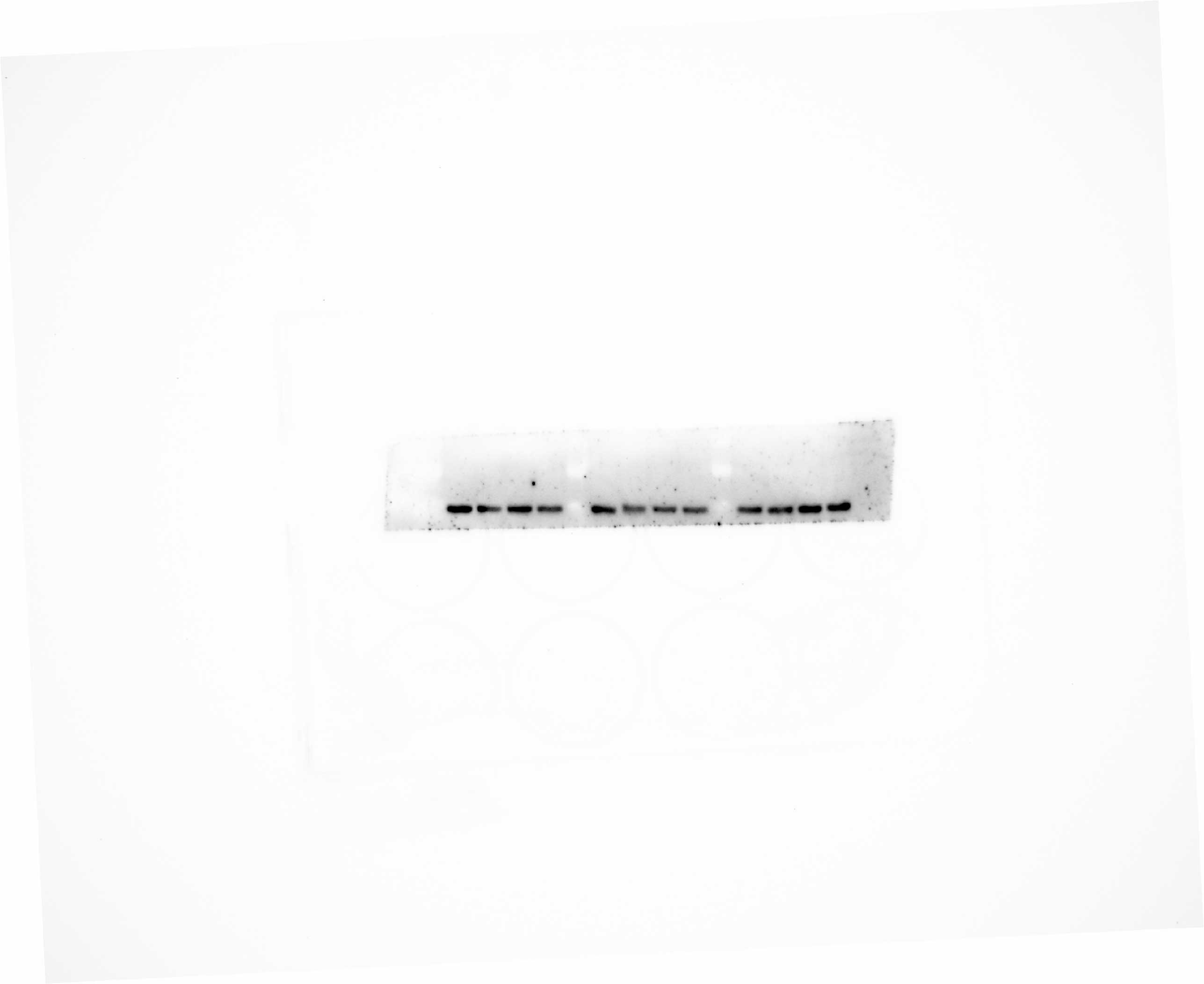

Supplement: Supplementary file 1 — Supplementary Material 1. [file 13046_2026_3724_MOESM1_ESM.zip › WB tiff/ABC-P65-Tubulin.jpg]

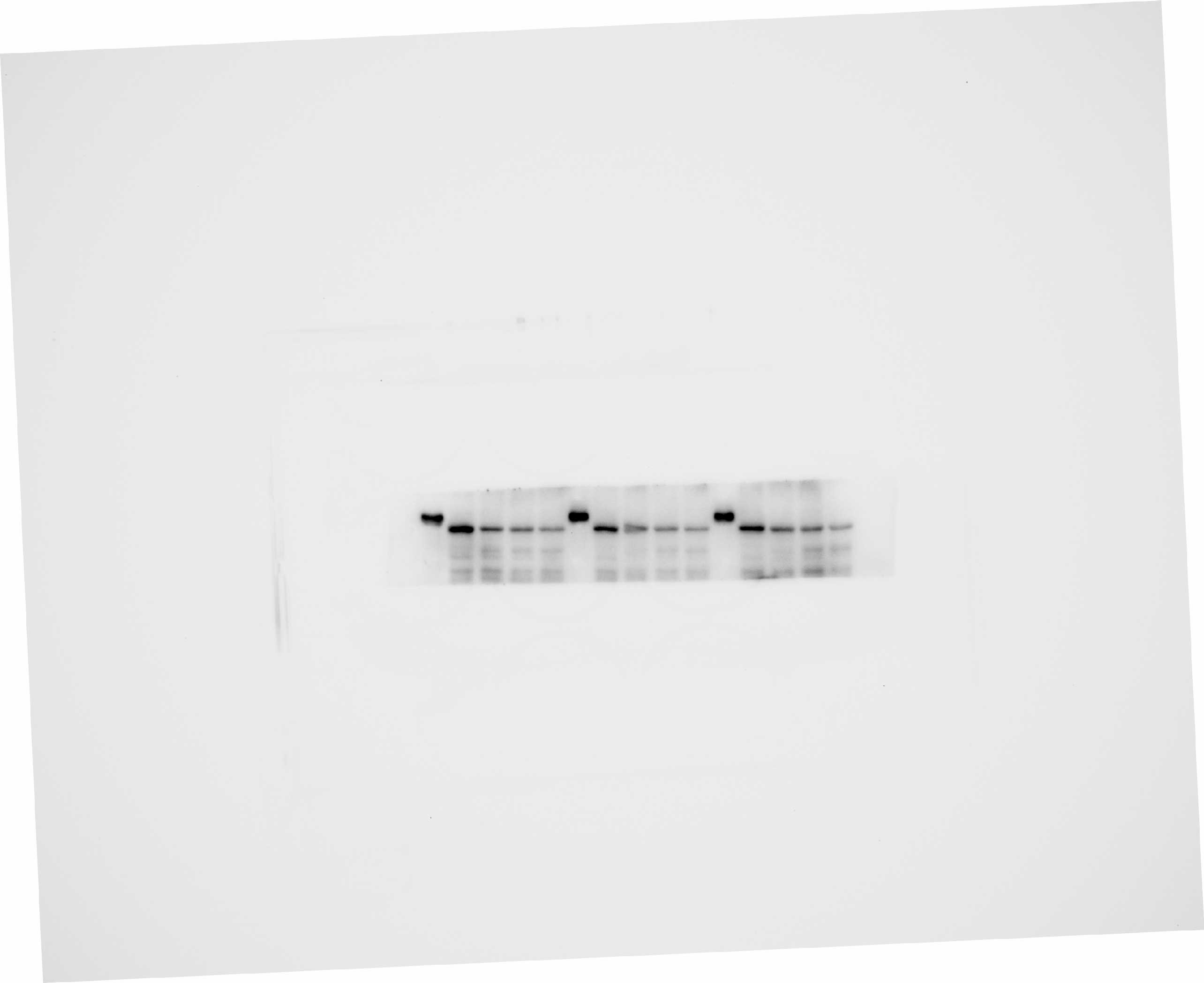

Supplement: Supplementary file 1 — Supplementary Material 1. [file 13046_2026_3724_MOESM1_ESM.zip › WB tiff/ABC-p65.jpg]

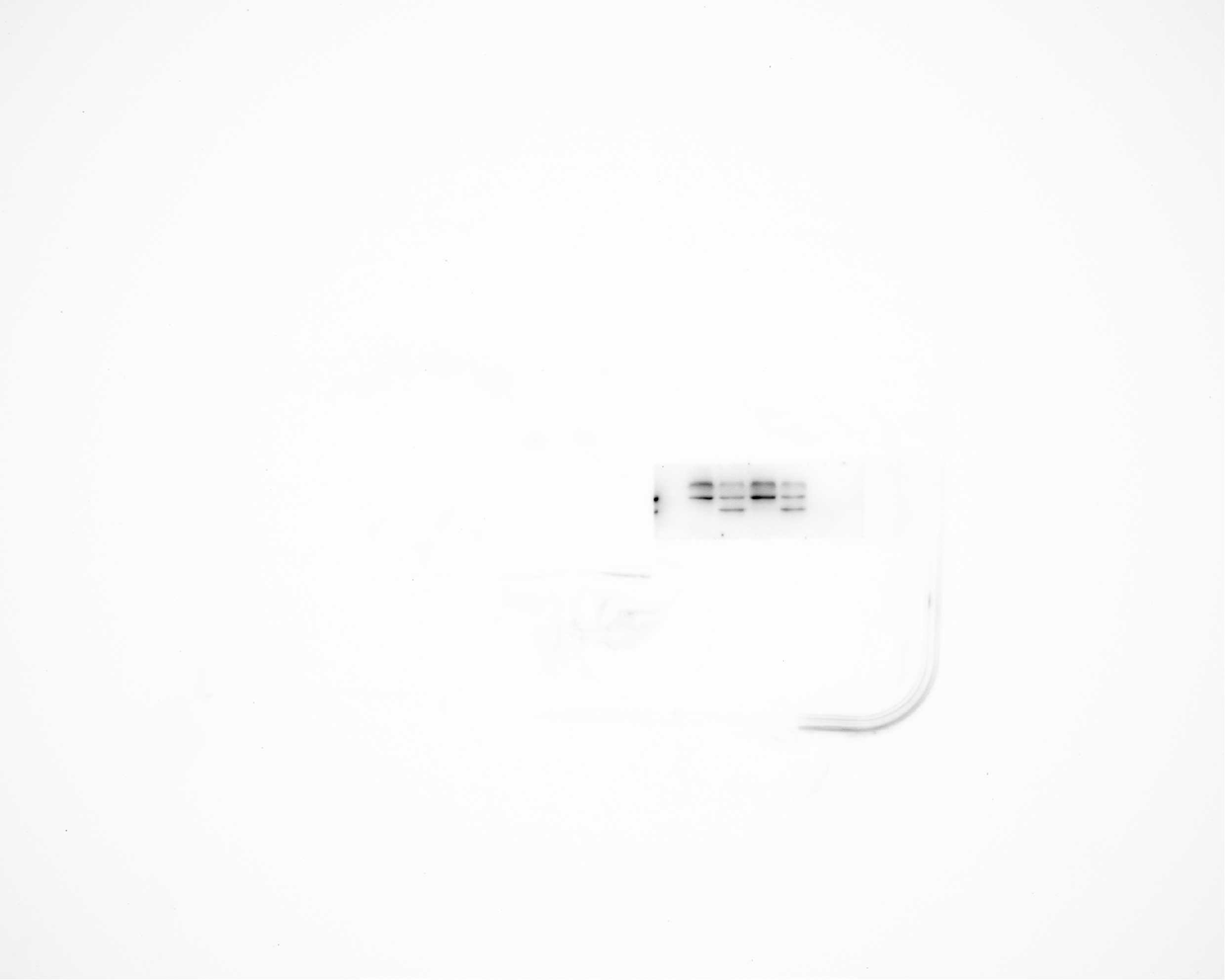

Supplement: Supplementary file 1 — Supplementary Material 1. [file 13046_2026_3724_MOESM1_ESM.zip › WB tiff/ABC-Parp.jpg]

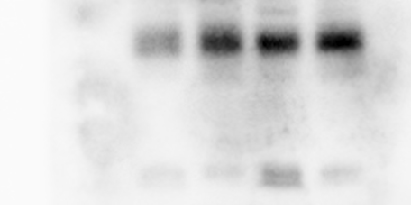

Supplement: Supplementary file 1 — Supplementary Material 1. [file 13046_2026_3724_MOESM1_ESM.zip › WB tiff/ATG-5 SUDHL4 SUDHL6.jpg]

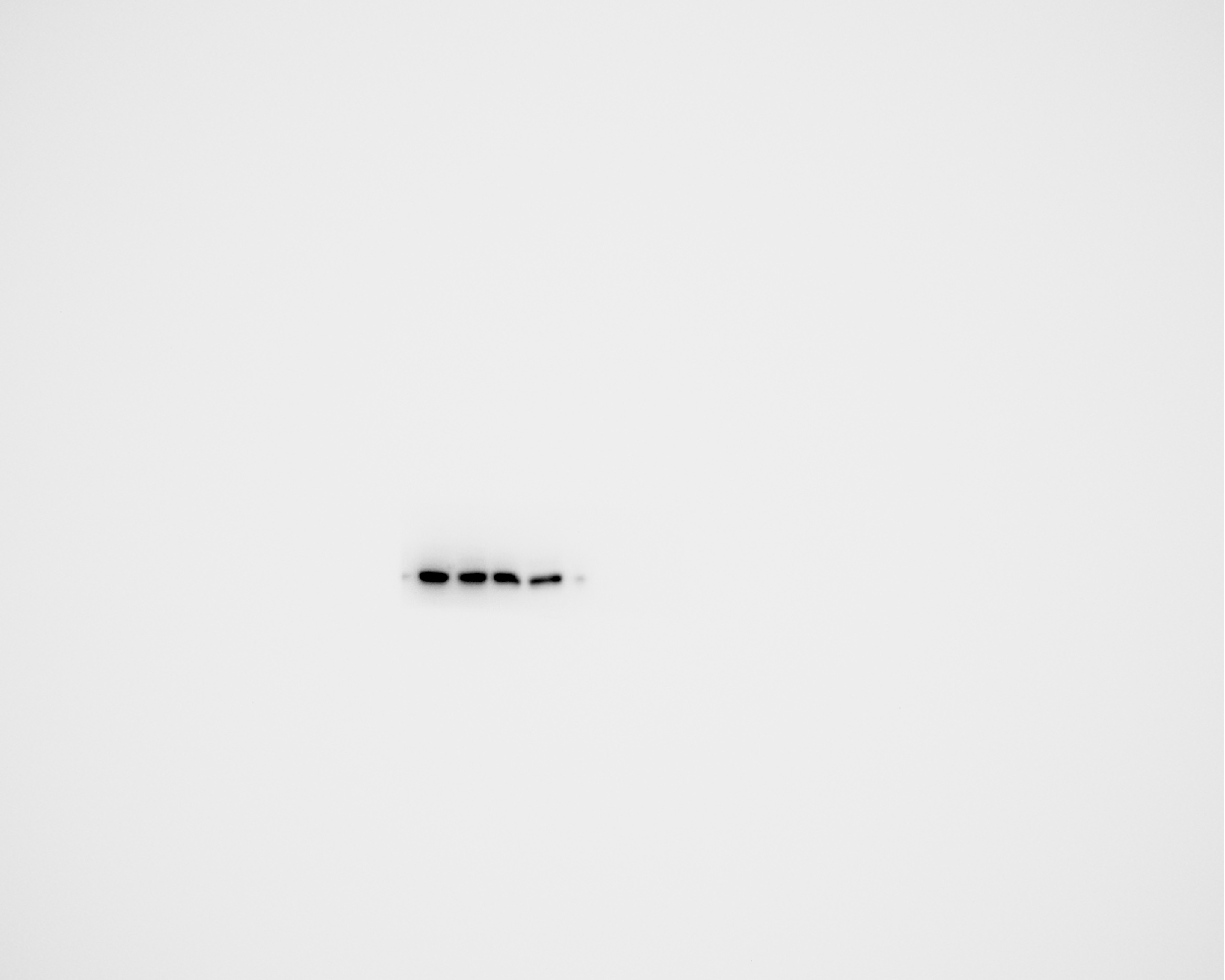

Supplement: Supplementary file 1 — Supplementary Material 1. [file 13046_2026_3724_MOESM1_ESM.zip › WB tiff/B-Tubulin-Cas3 SUDHL4 SUDHL6.jpg]

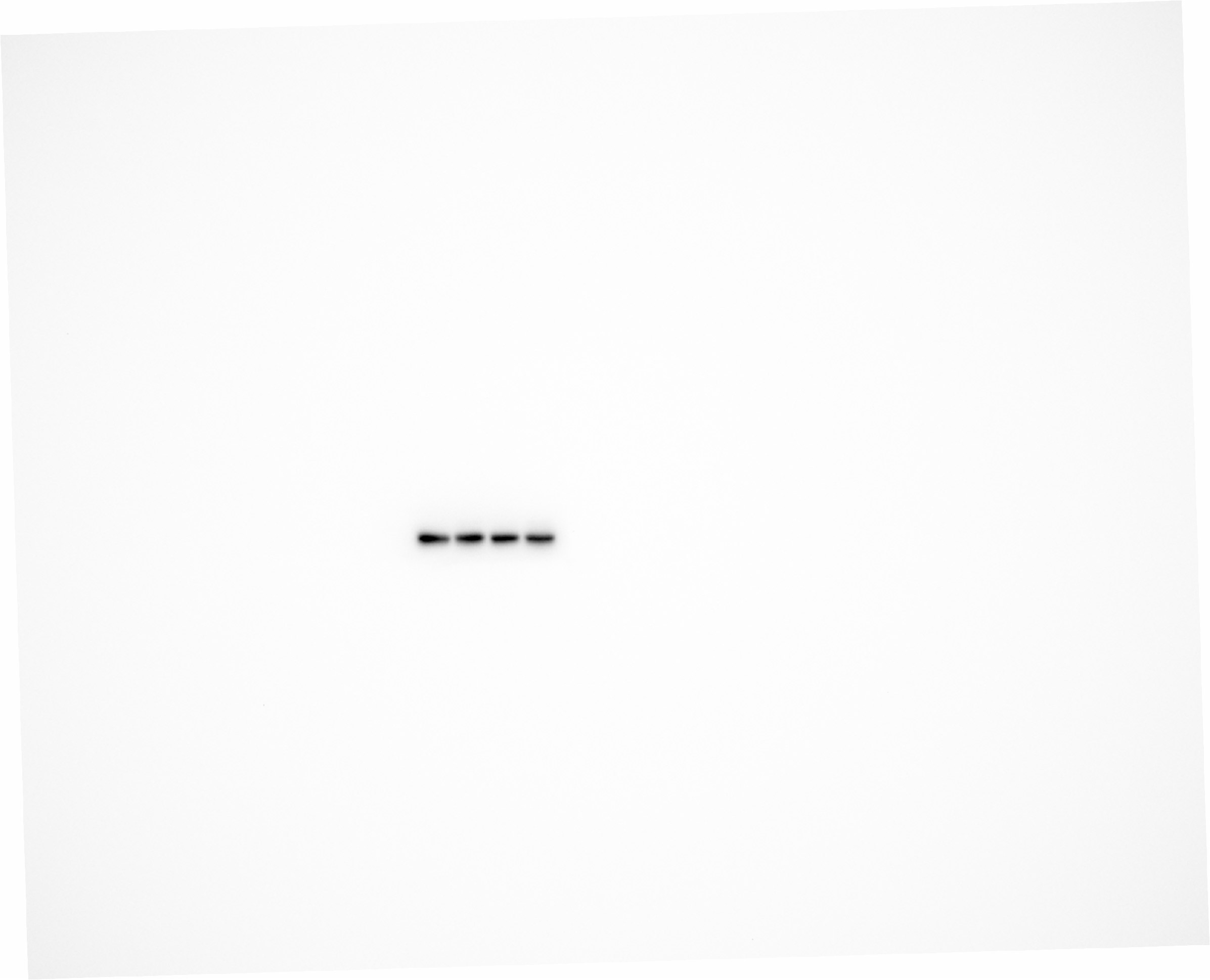

Supplement: Supplementary file 1 — Supplementary Material 1. [file 13046_2026_3724_MOESM1_ESM.zip › WB tiff/B-Tubulin-cas9-MCL1 SUDHL4 SUDHL6.jpg]

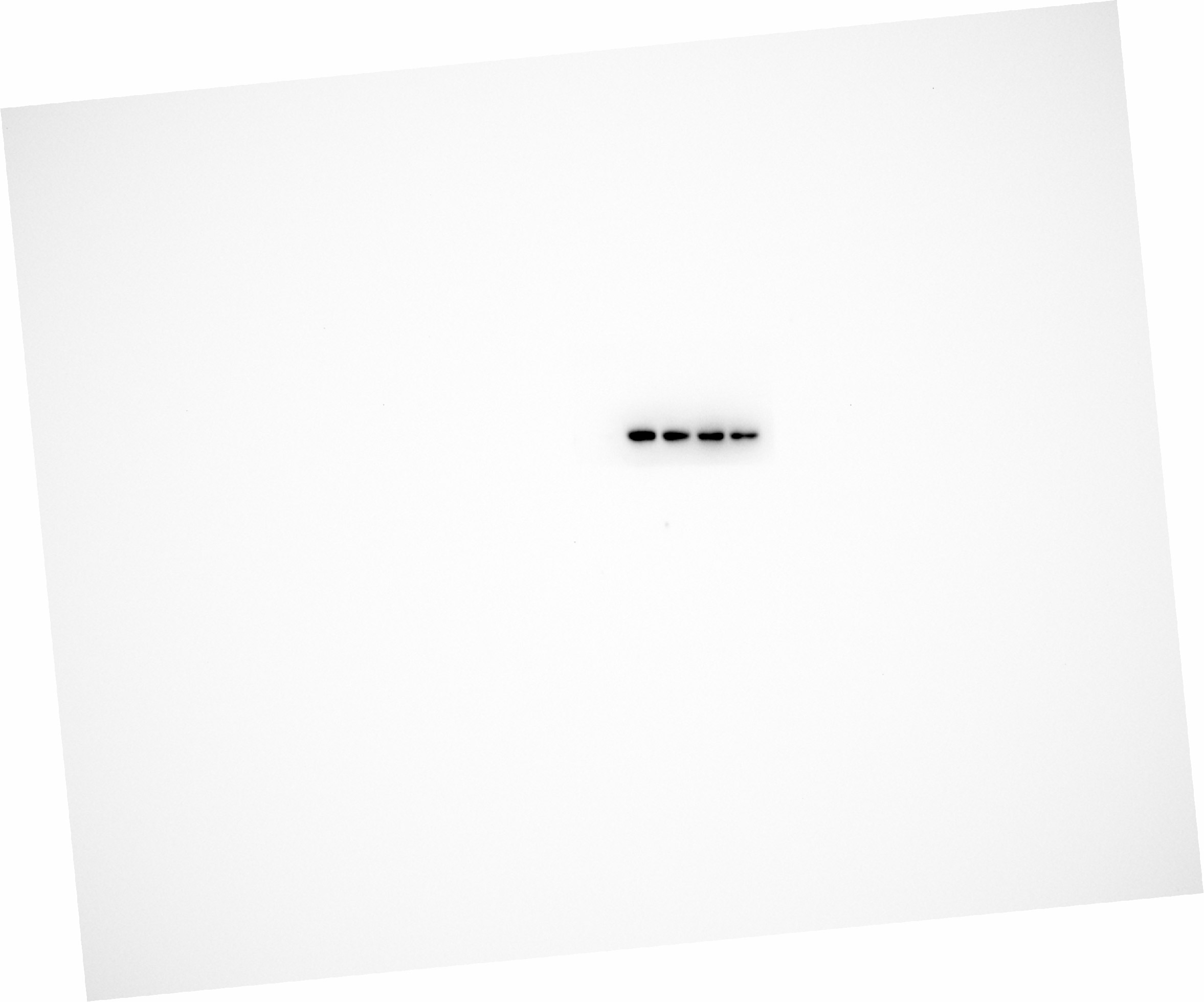

Supplement: Supplementary file 1 — Supplementary Material 1. [file 13046_2026_3724_MOESM1_ESM.zip › WB tiff/B-Tubulin-p21 SUDHL4 SUDHL6.jpg]

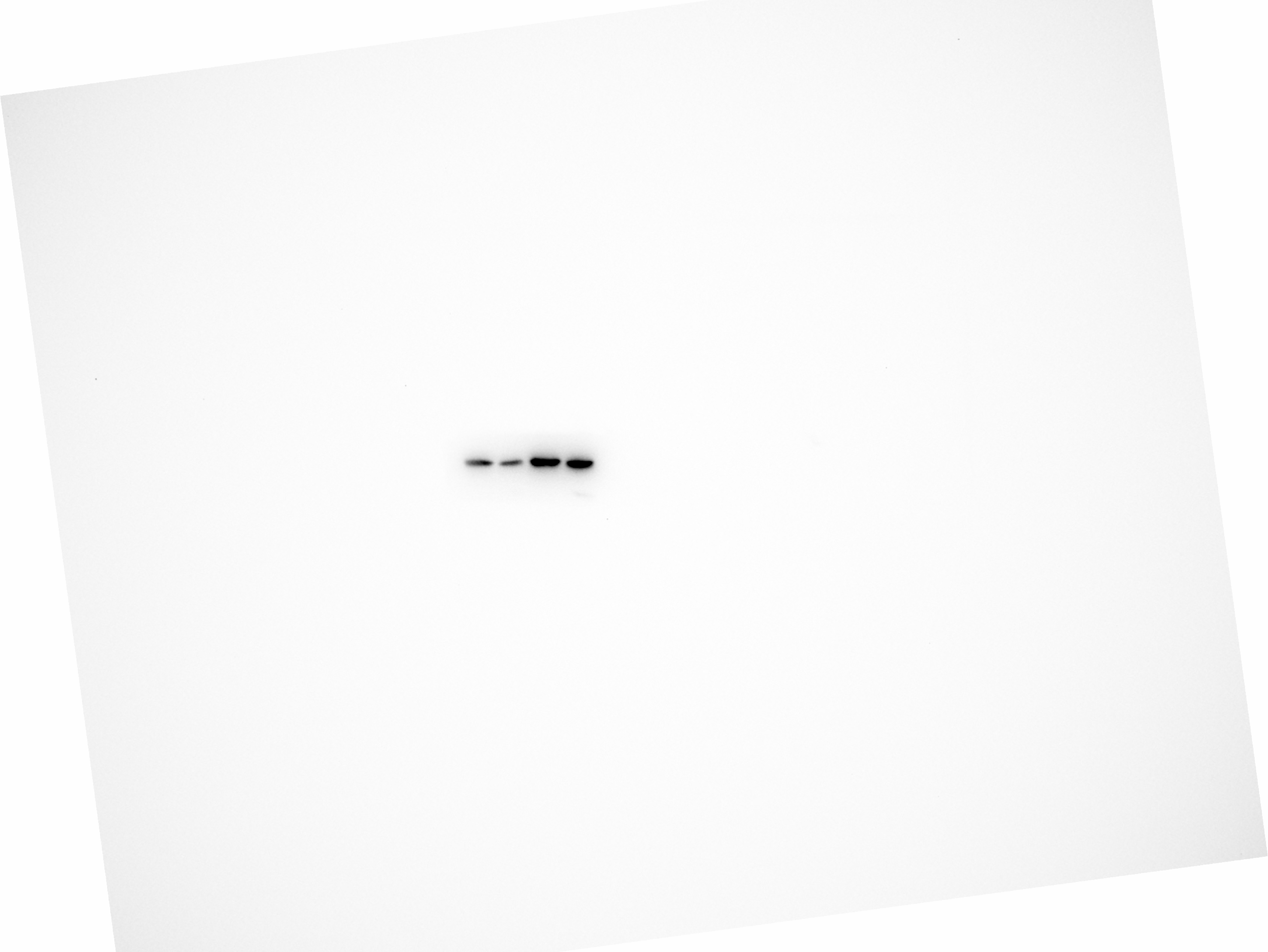

Supplement: Supplementary file 1 — Supplementary Material 1. [file 13046_2026_3724_MOESM1_ESM.zip › WB tiff/BCL2 SUDHL4 SUDHL6.jpg]

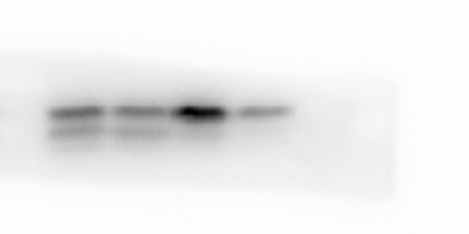

Supplement: Supplementary file 1 — Supplementary Material 1. [file 13046_2026_3724_MOESM1_ESM.zip › WB tiff/Beclin-1 SUDHL4 SUDHL6.jpg]

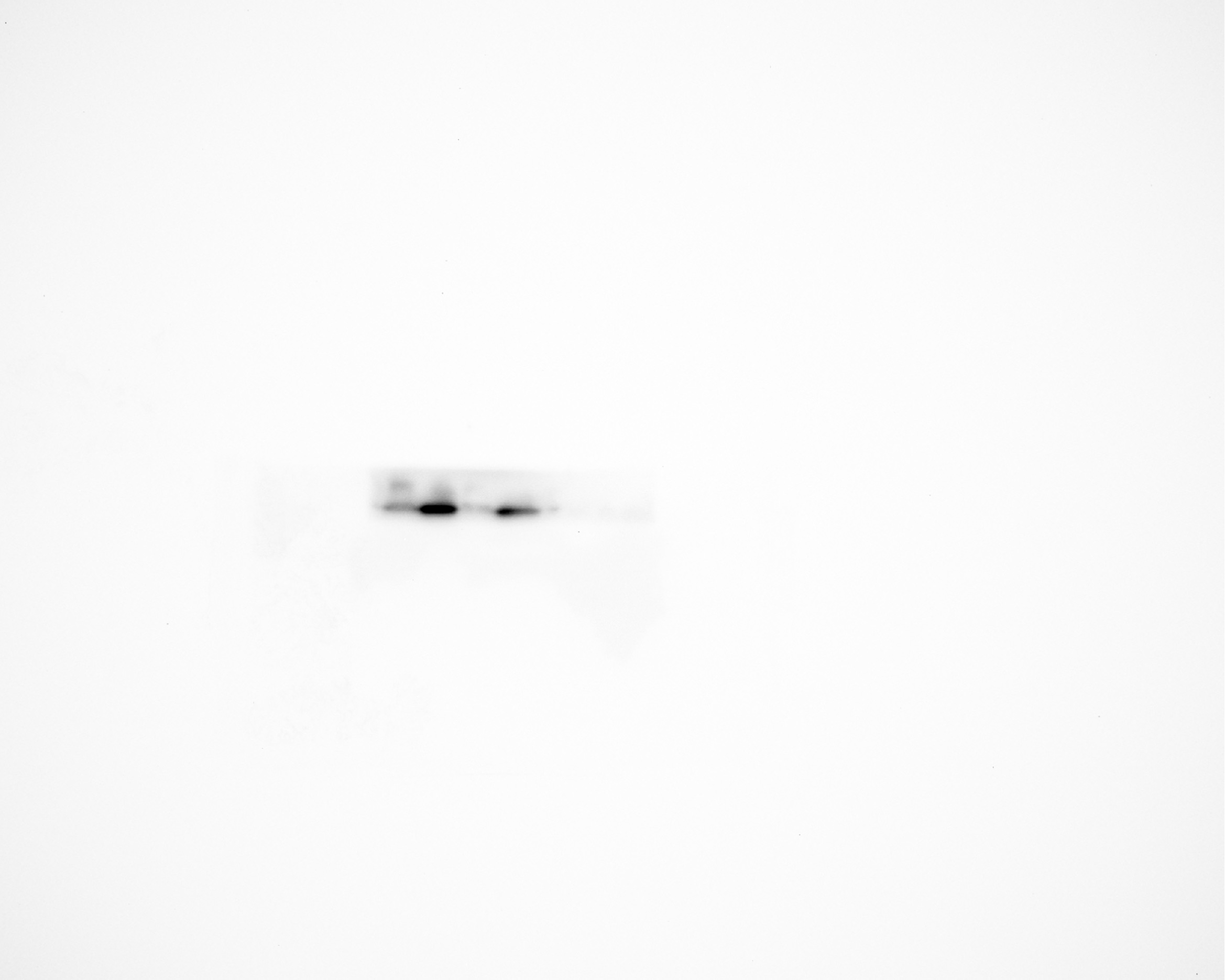

Supplement: Supplementary file 1 — Supplementary Material 1. [file 13046_2026_3724_MOESM1_ESM.zip › WB tiff/Cas3-Cleaved SUDHL4 SUDHL6.jpg]

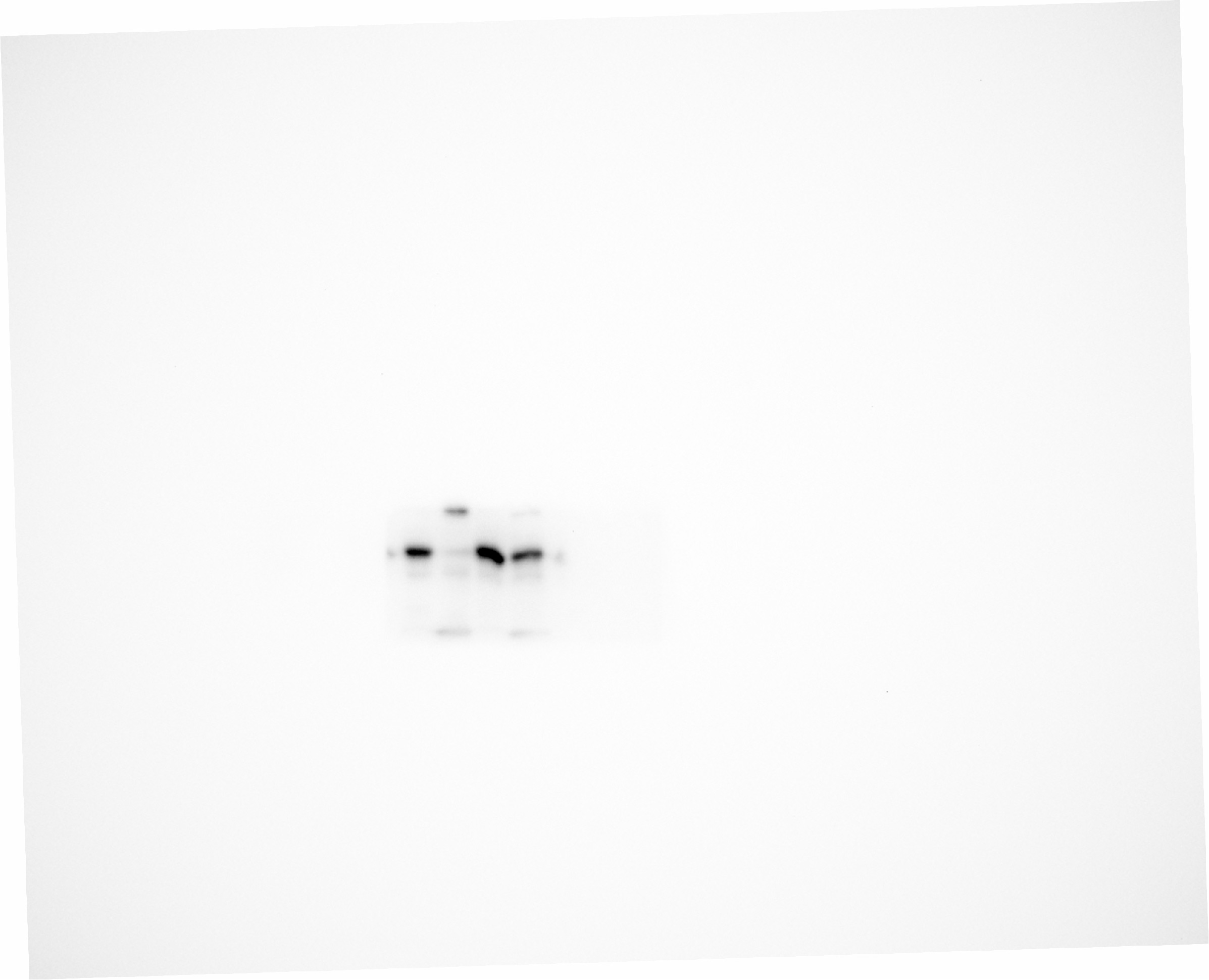

Supplement: Supplementary file 1 — Supplementary Material 1. [file 13046_2026_3724_MOESM1_ESM.zip › WB tiff/Cas3-Pro SUDHL4 SUDHL6.jpg]

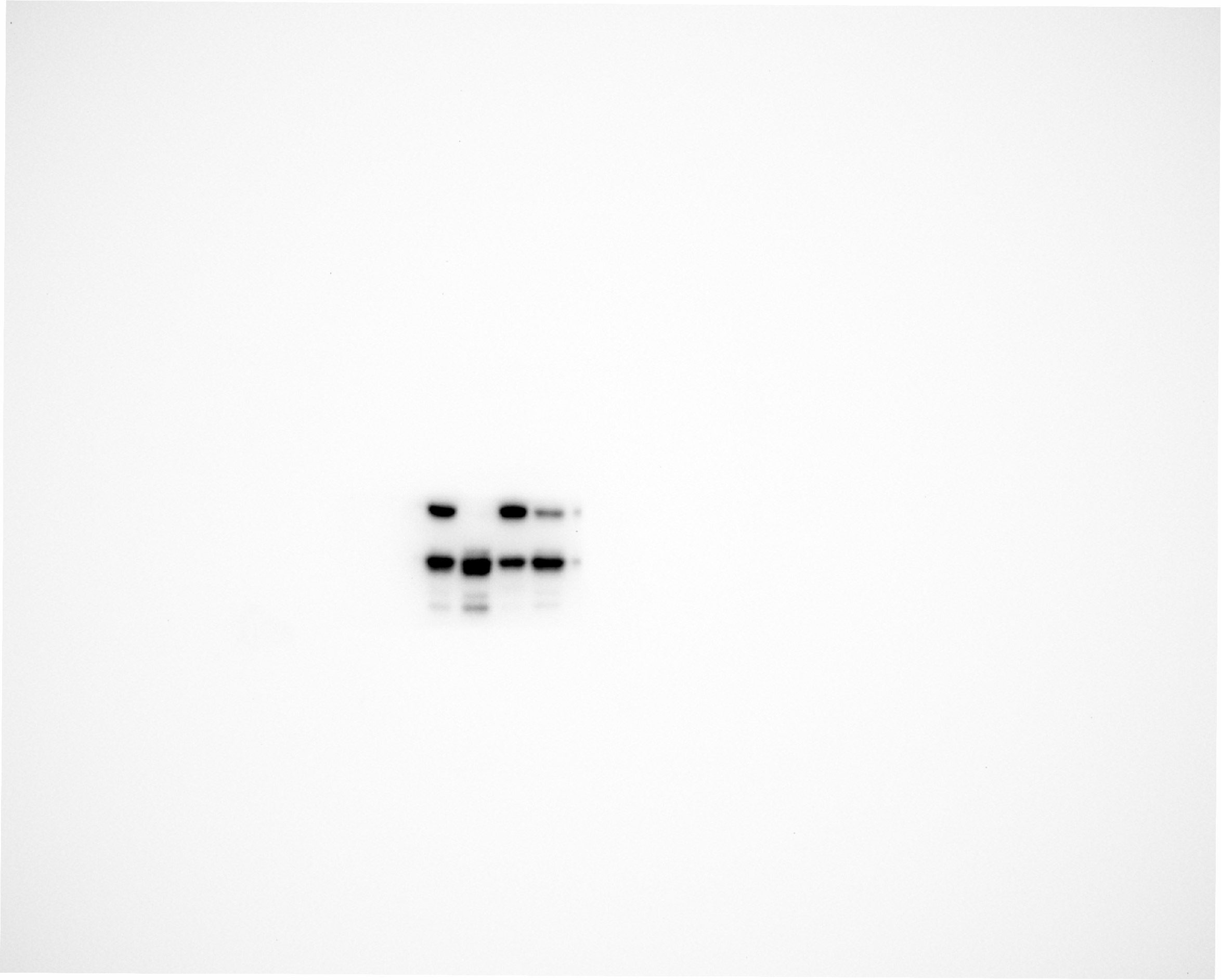

Supplement: Supplementary file 1 — Supplementary Material 1. [file 13046_2026_3724_MOESM1_ESM.zip › WB tiff/Cas9 SUDHL4 SUDHL6.jpg]

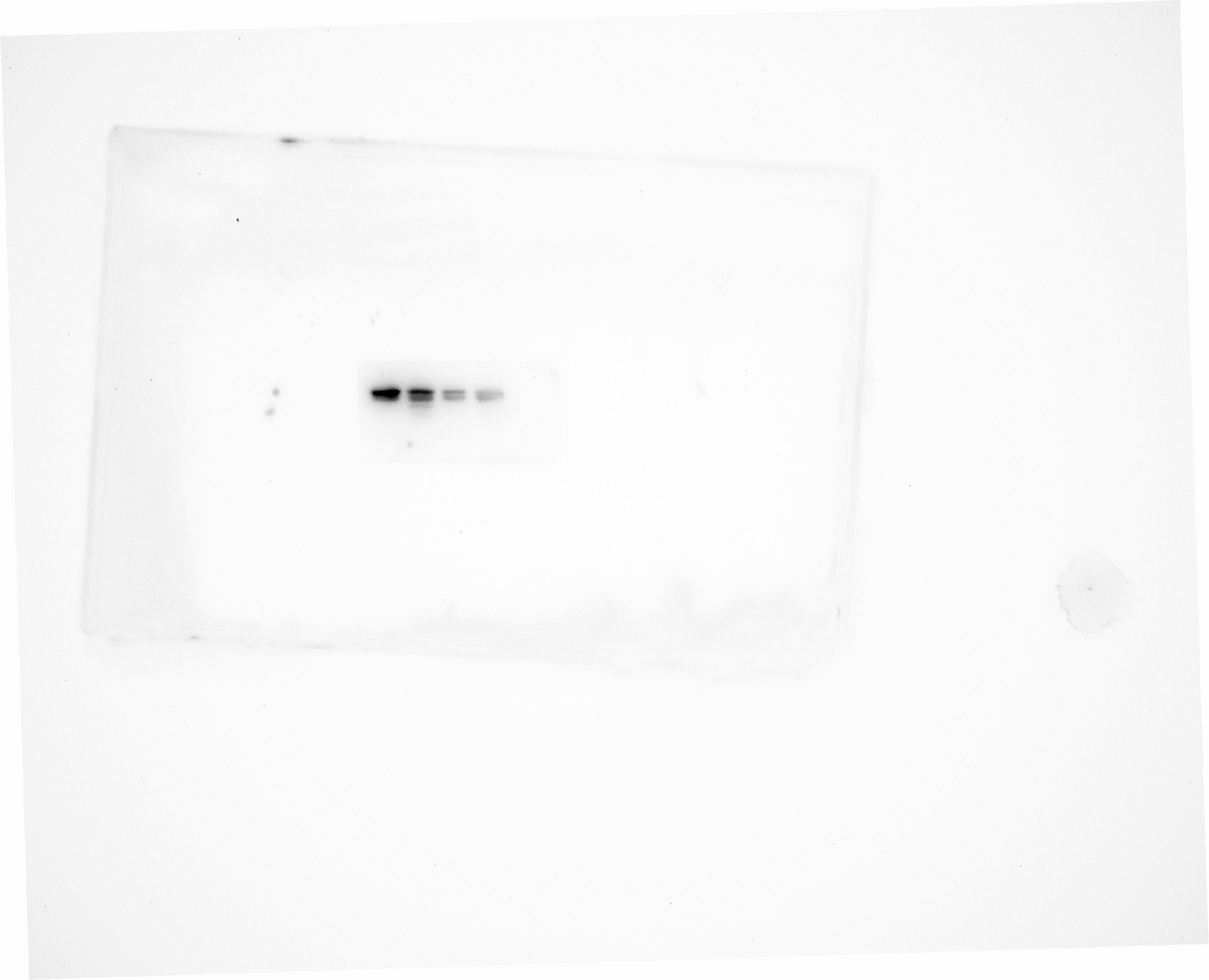

Supplement: Supplementary file 1 — Supplementary Material 1. [file 13046_2026_3724_MOESM1_ESM.zip › WB tiff/CDK6 SUDHL4 SUDHL6.jpg]

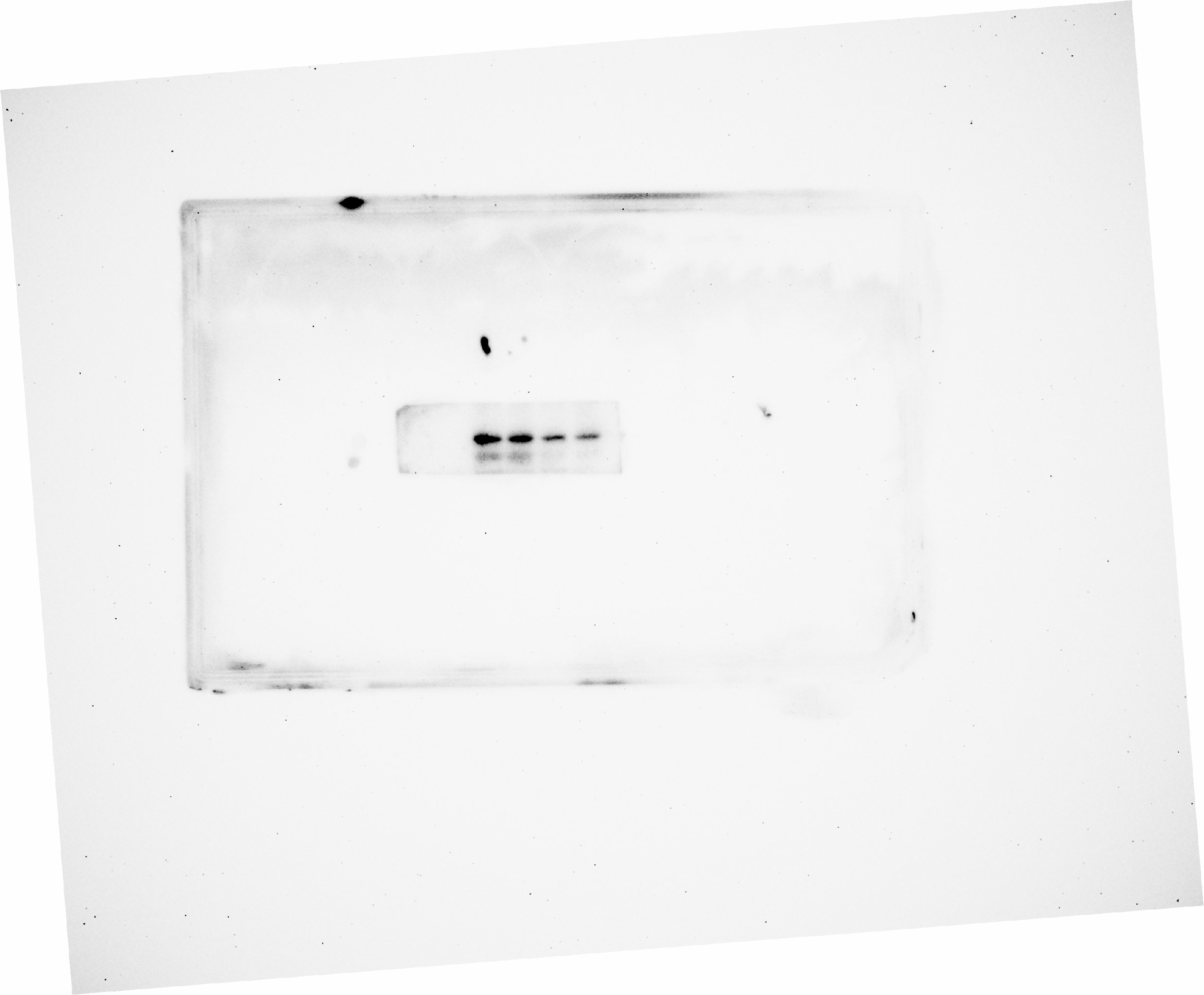

Supplement: Supplementary file 1 — Supplementary Material 1. [file 13046_2026_3724_MOESM1_ESM.zip › WB tiff/CyclinD1 SUDHL4 SUDHL6.jpg]

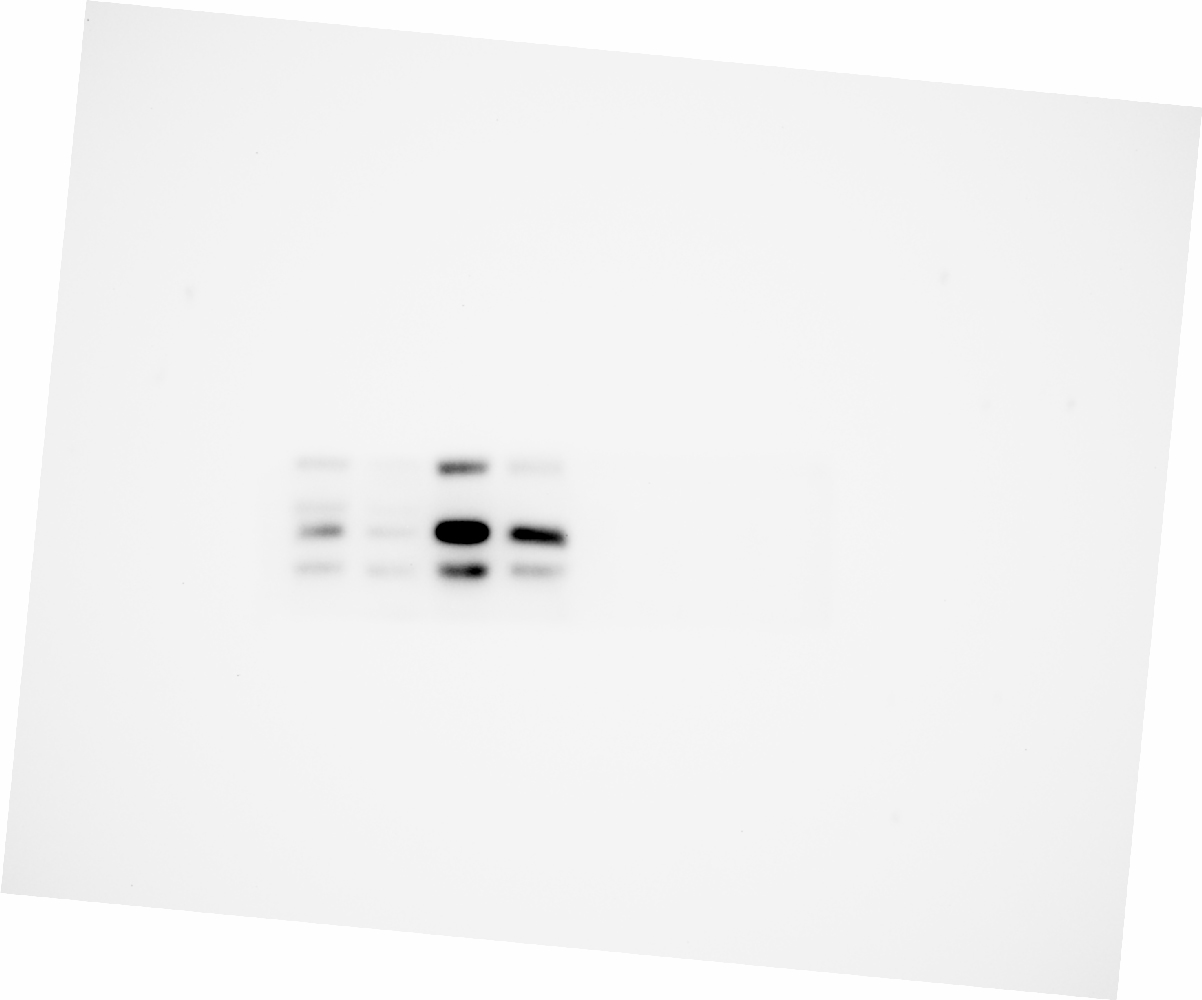

Supplement: Supplementary file 1 — Supplementary Material 1. [file 13046_2026_3724_MOESM1_ESM.zip › WB tiff/CyclinE1 SUDHL4 SUDHL6.jpg]

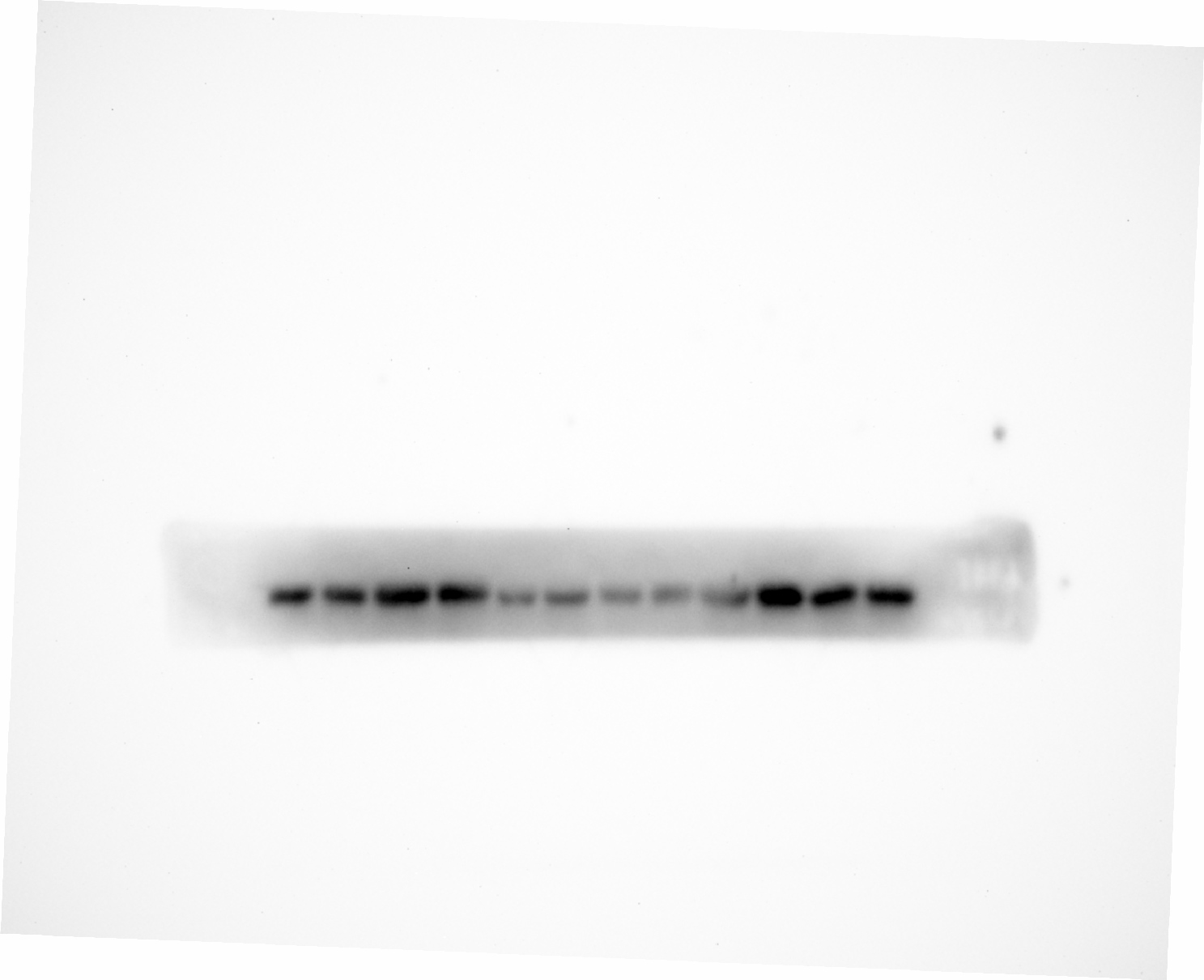

Supplement: Supplementary file 1 — Supplementary Material 1. [file 13046_2026_3724_MOESM1_ESM.zip › WB tiff/GA -IKBa-p65 SUDHL4 SUDHL6.jpg]

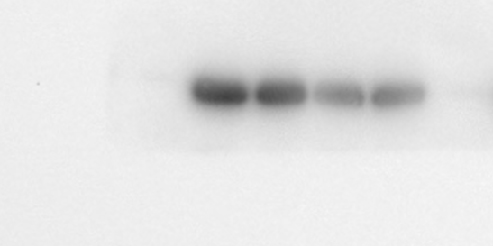

Supplement: Supplementary file 1 — Supplementary Material 1. [file 13046_2026_3724_MOESM1_ESM.zip › WB tiff/GA-ATG5 SUDHL4 SUDHL6.jpg]

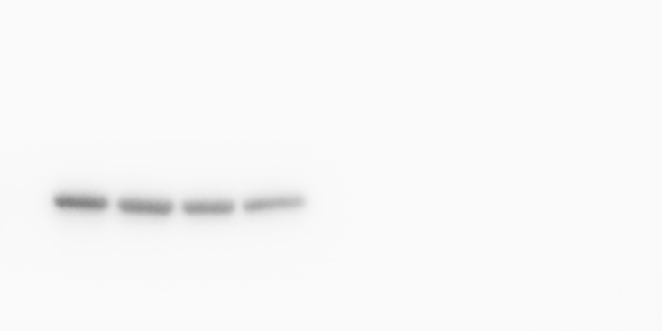

Supplement: Supplementary file 1 — Supplementary Material 1. [file 13046_2026_3724_MOESM1_ESM.zip › WB tiff/GA-Beclin1 SUDHL4 SUDHL6.jpg]

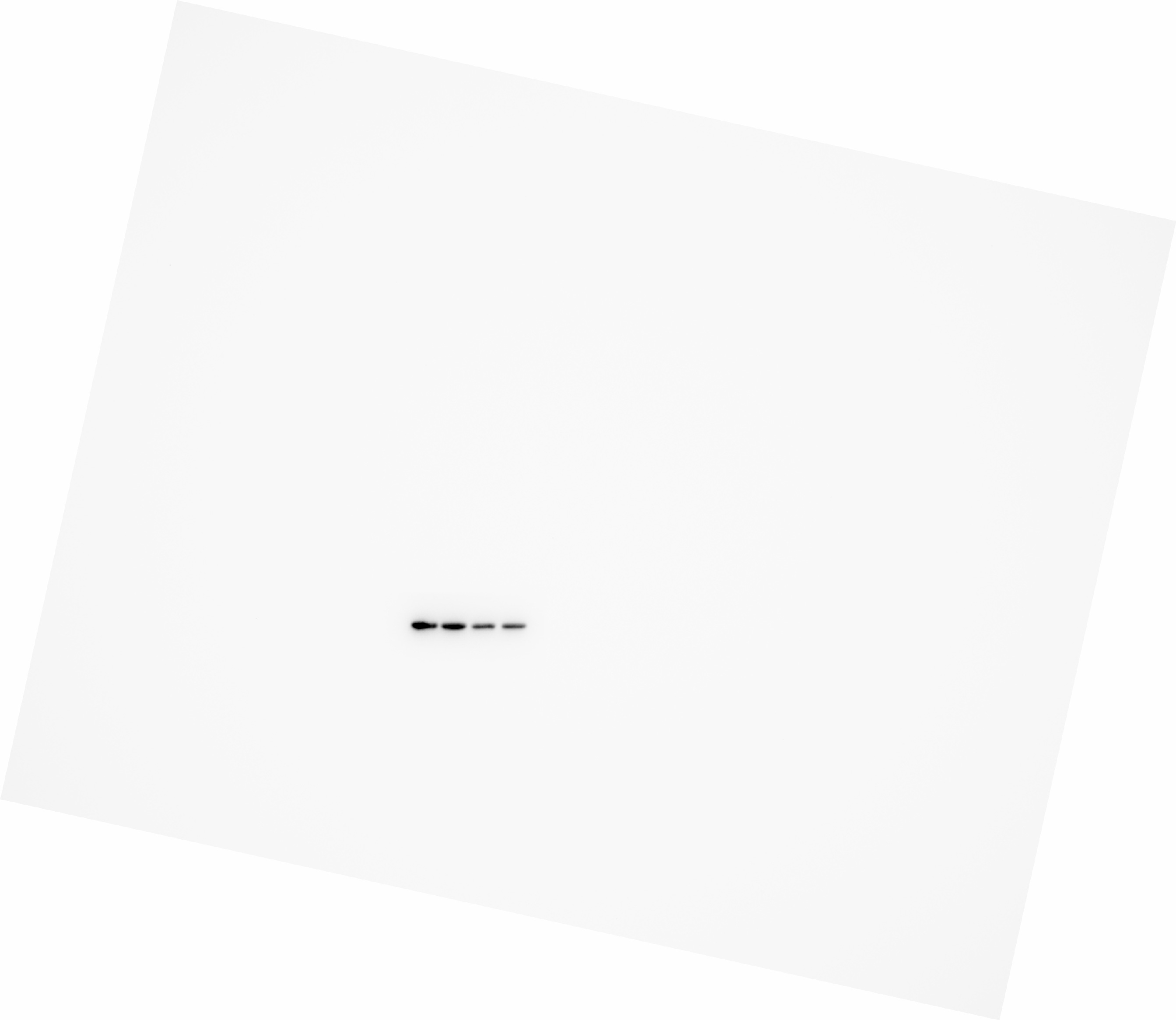

Supplement: Supplementary file 1 — Supplementary Material 1. [file 13046_2026_3724_MOESM1_ESM.zip › WB tiff/Ga-D1 SUDHL4 SUDHL6.jpg]

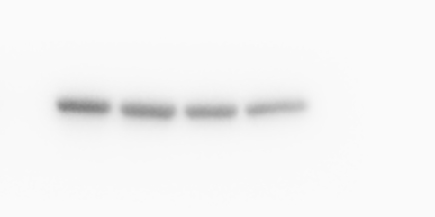

Supplement: Supplementary file 1 — Supplementary Material 1. [file 13046_2026_3724_MOESM1_ESM.zip › WB tiff/GA-GABARAPL1 SUDHL4 SUDHL6.jpg]

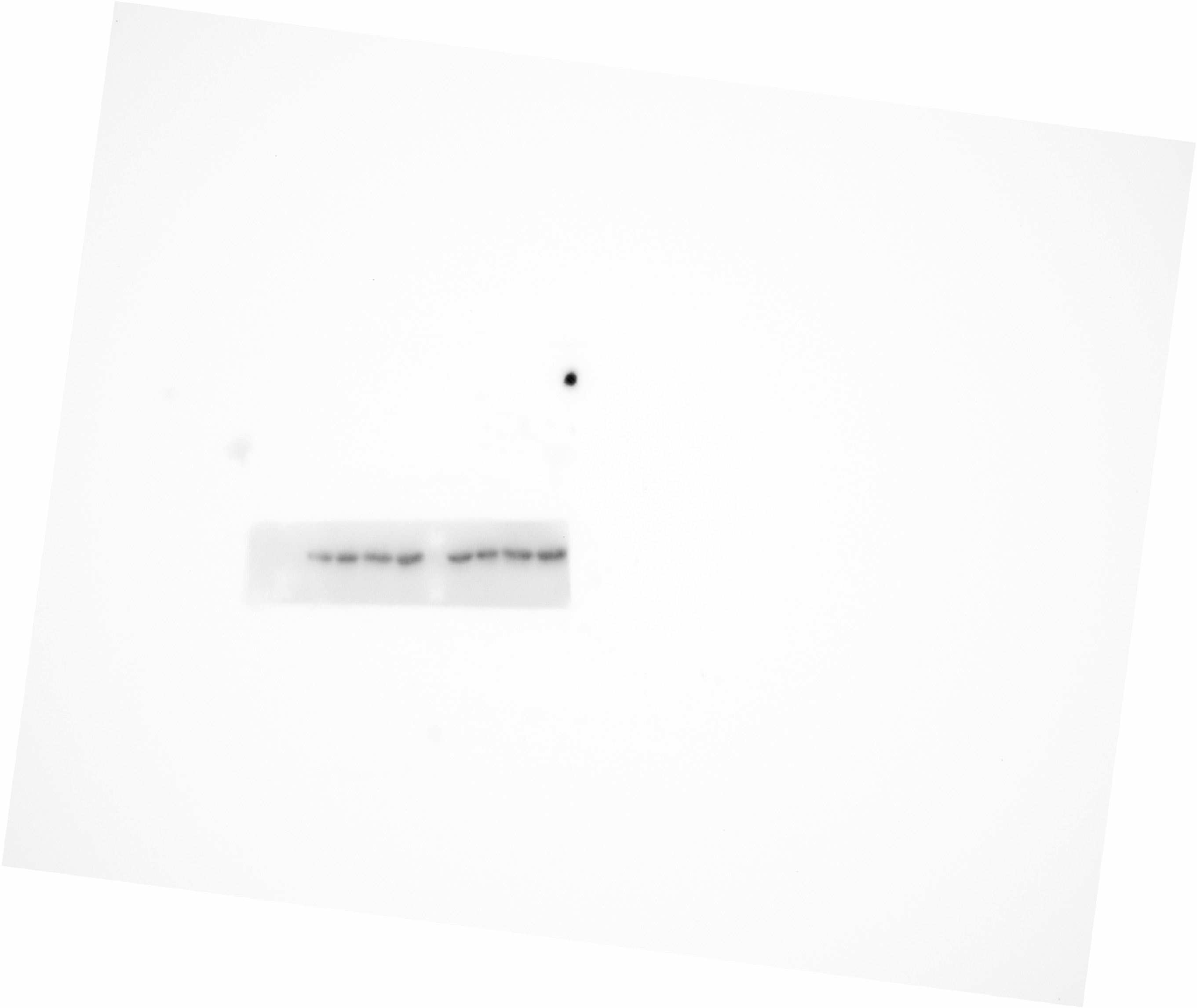

Supplement: Supplementary file 1 — Supplementary Material 1. [file 13046_2026_3724_MOESM1_ESM.zip › WB tiff/GA-IKKa SUDHL4 SUDHL6.jpg]

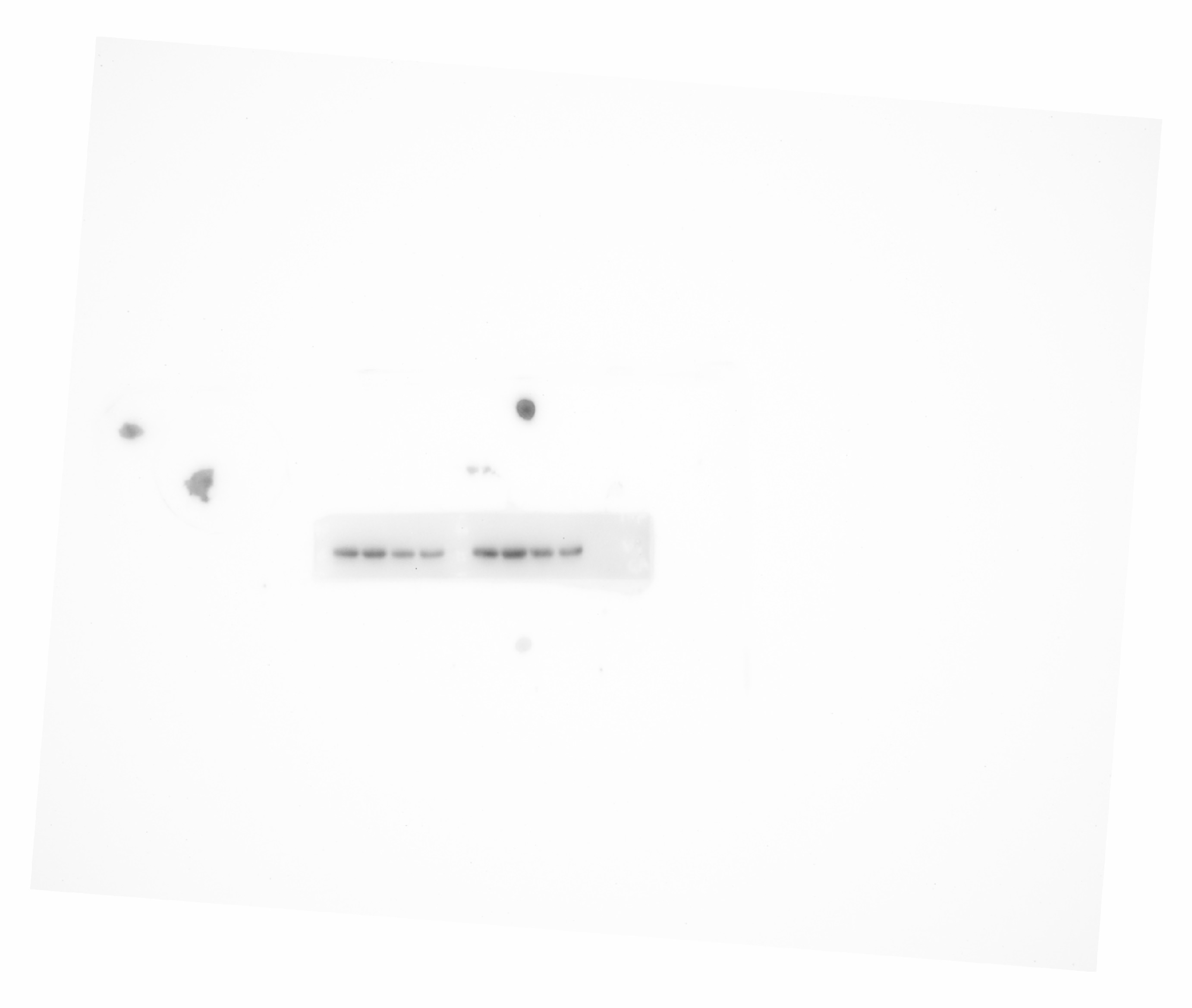

Supplement: Supplementary file 1 — Supplementary Material 1. [file 13046_2026_3724_MOESM1_ESM.zip › WB tiff/GA-IKKb SUDHL4 SUDHL6.jpg]

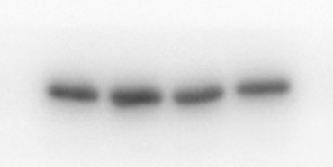

Supplement: Supplementary file 1 — Supplementary Material 1. [file 13046_2026_3724_MOESM1_ESM.zip › WB tiff/GA-LC3-p62 SUDHL4 SUDHL6.jpg]

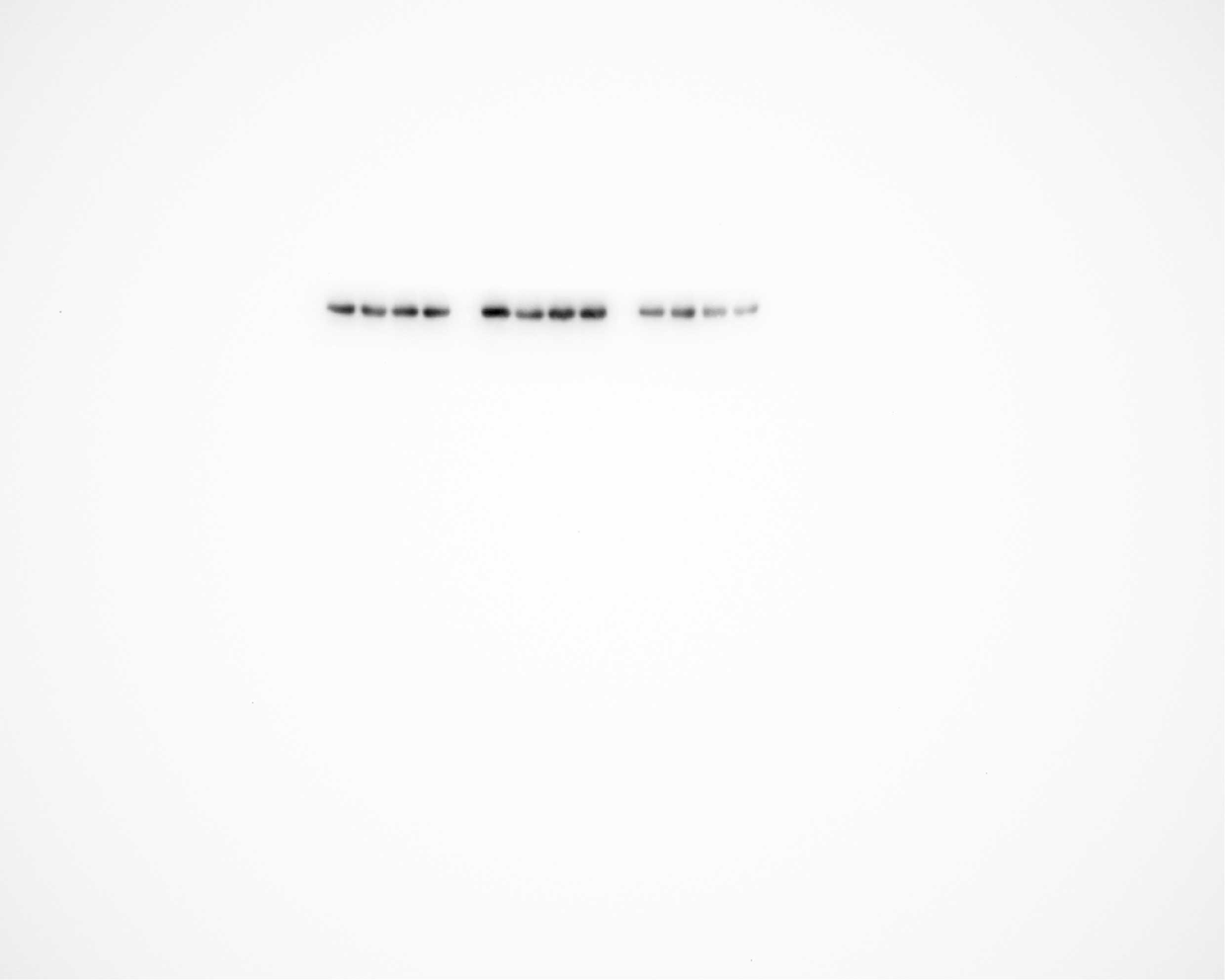

Supplement: Supplementary file 1 — Supplementary Material 1. [file 13046_2026_3724_MOESM1_ESM.zip › WB tiff/Ga-NAC-U2932.jpg]

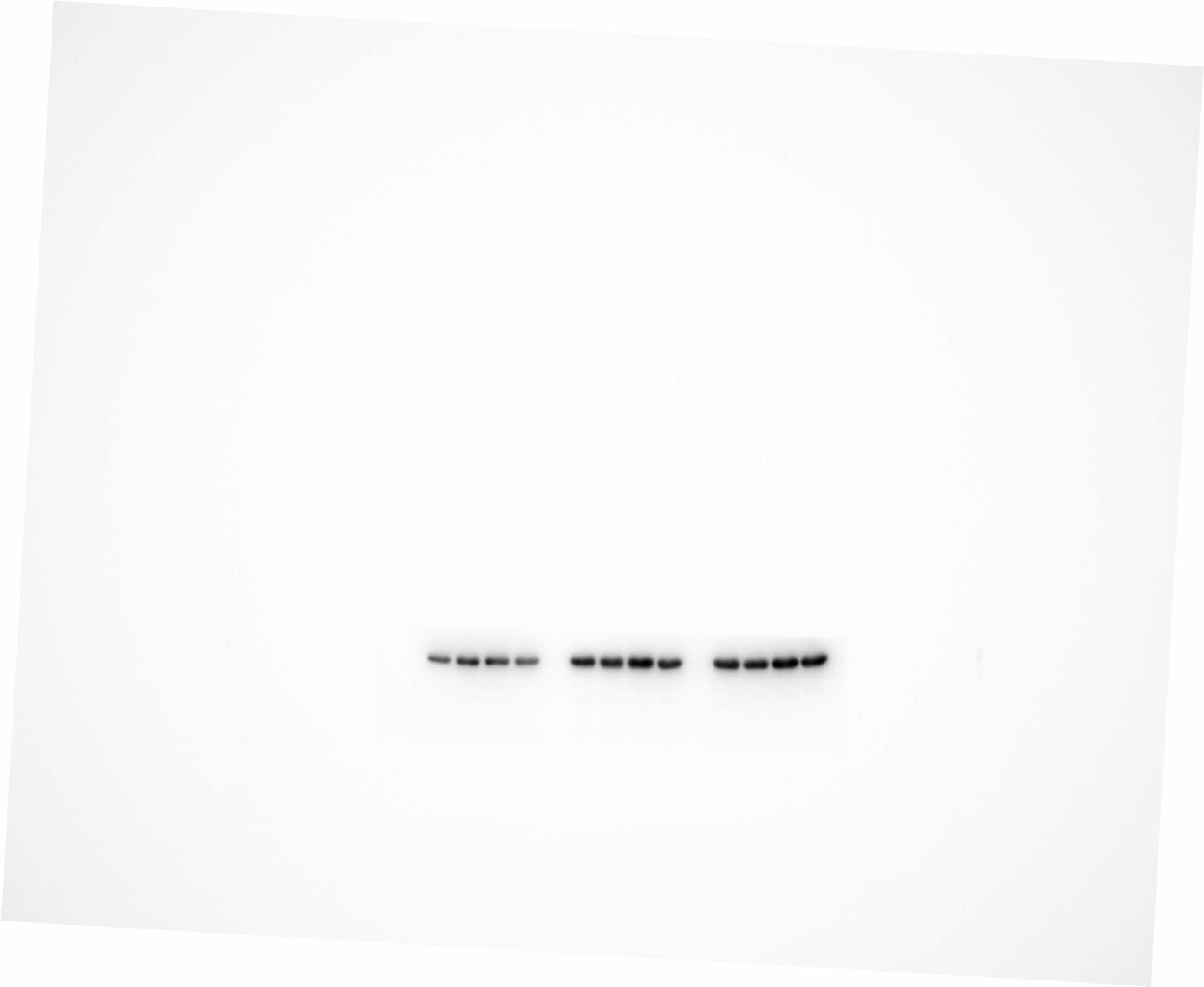

Supplement: Supplementary file 1 — Supplementary Material 1. [file 13046_2026_3724_MOESM1_ESM.zip › WB tiff/GA-NACP-65-IKBa-SU4.jpg]

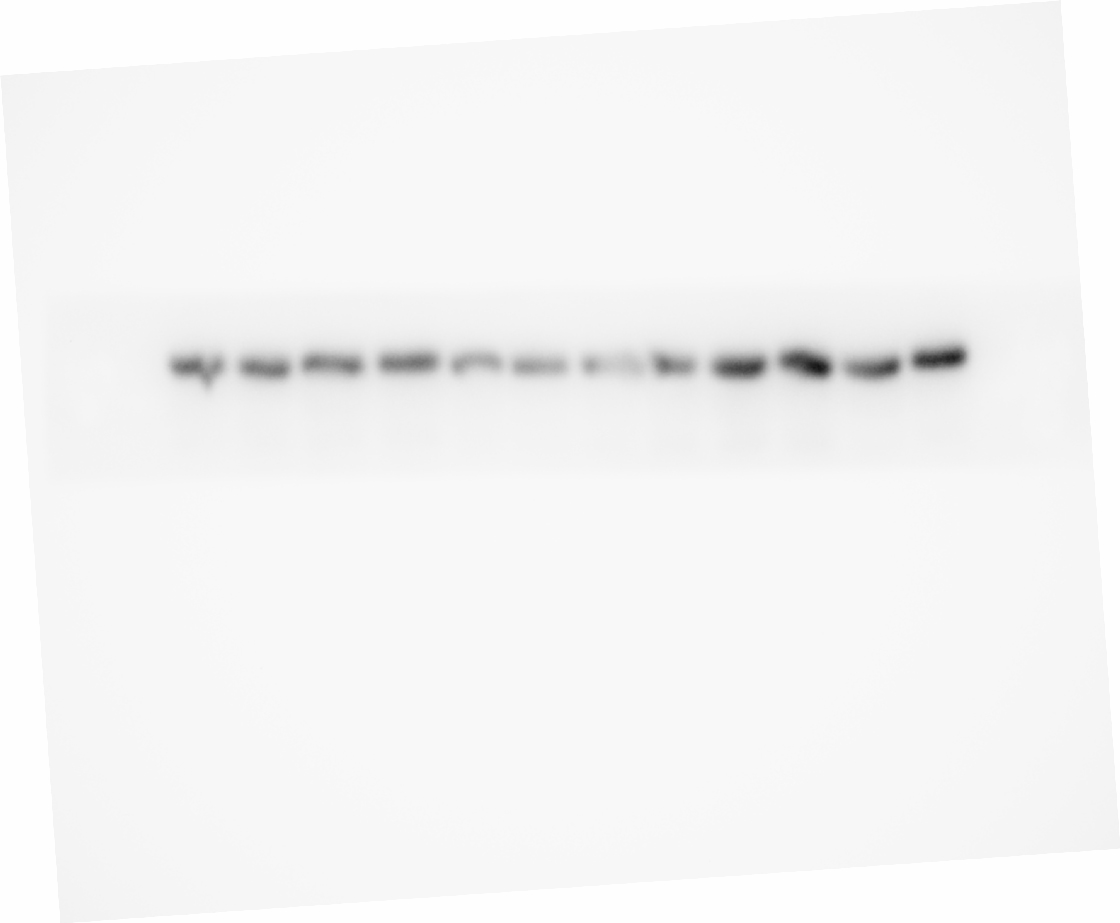

Supplement: Supplementary file 1 — Supplementary Material 1. [file 13046_2026_3724_MOESM1_ESM.zip › WB tiff/GA-p105p50 SUDHL4 SUDHL6.jpg]

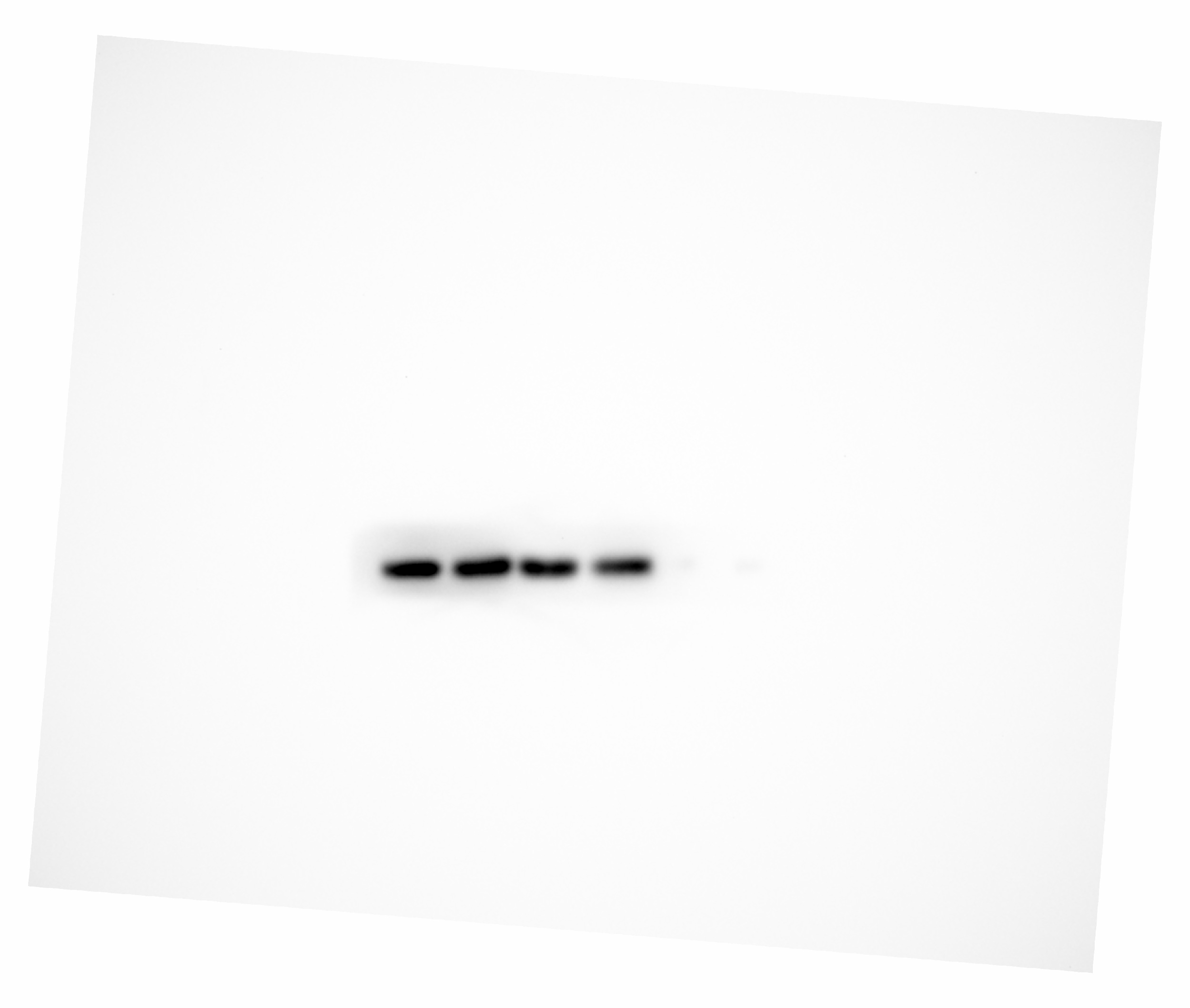

Supplement: Supplementary file 1 — Supplementary Material 1. [file 13046_2026_3724_MOESM1_ESM.zip › WB tiff/GA-p27-E1 SUDHL4 SUDHL6.jpg]

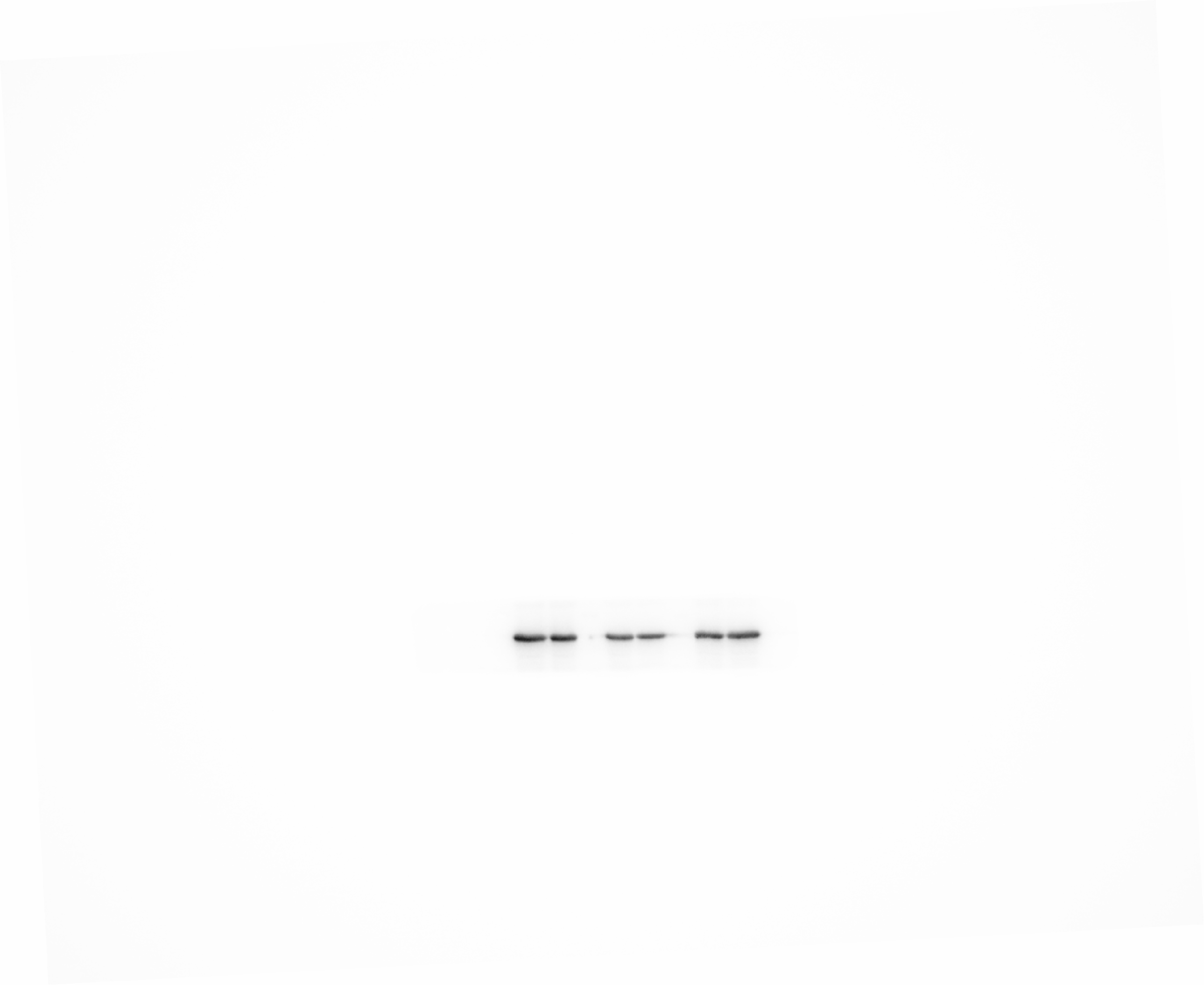

Supplement: Supplementary file 1 — Supplementary Material 1. [file 13046_2026_3724_MOESM1_ESM.zip › WB tiff/GA-p65OE(SU4).jpg]

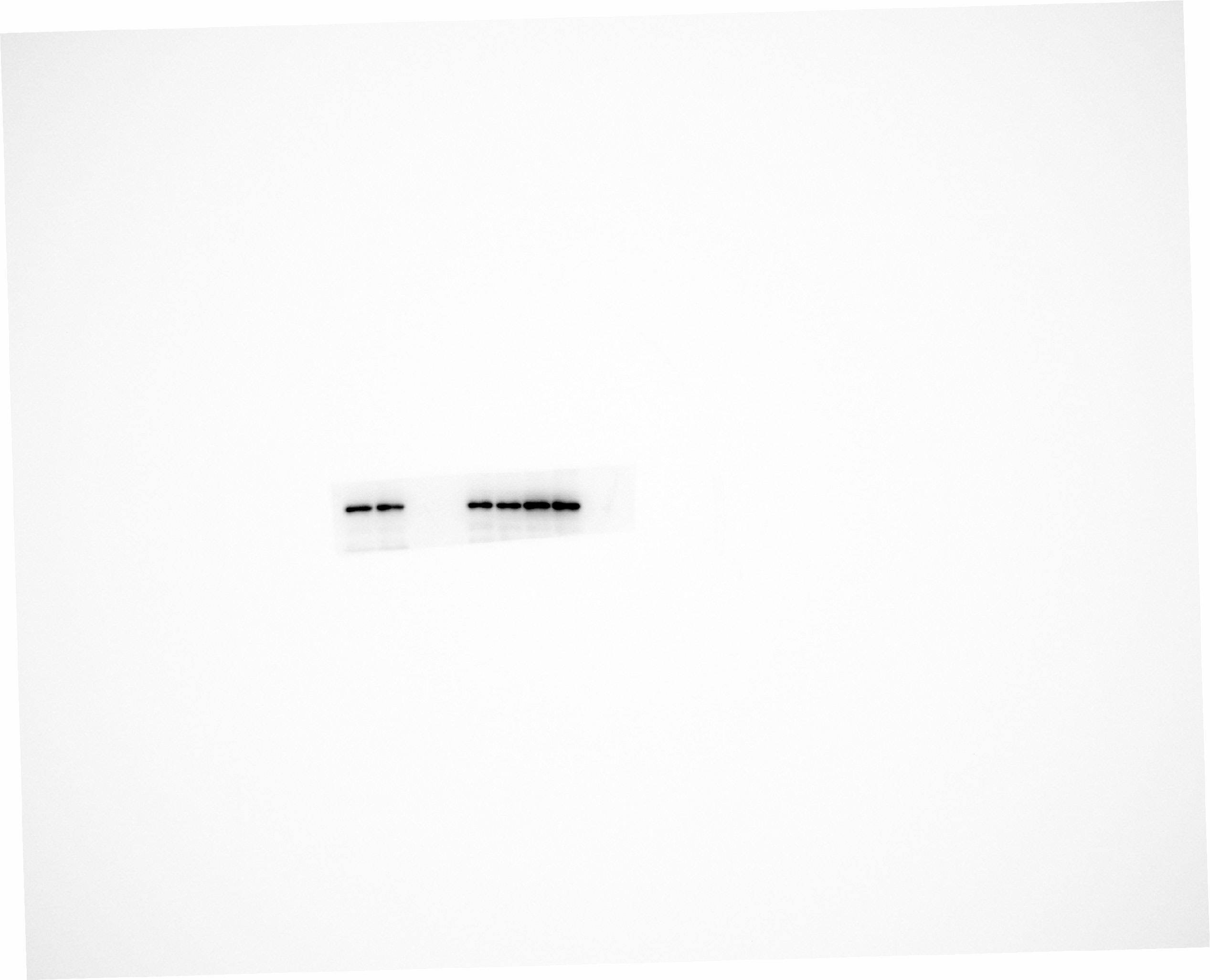

Supplement: Supplementary file 1 — Supplementary Material 1. [file 13046_2026_3724_MOESM1_ESM.zip › WB tiff/GA-p65OE(SU6).jpg]

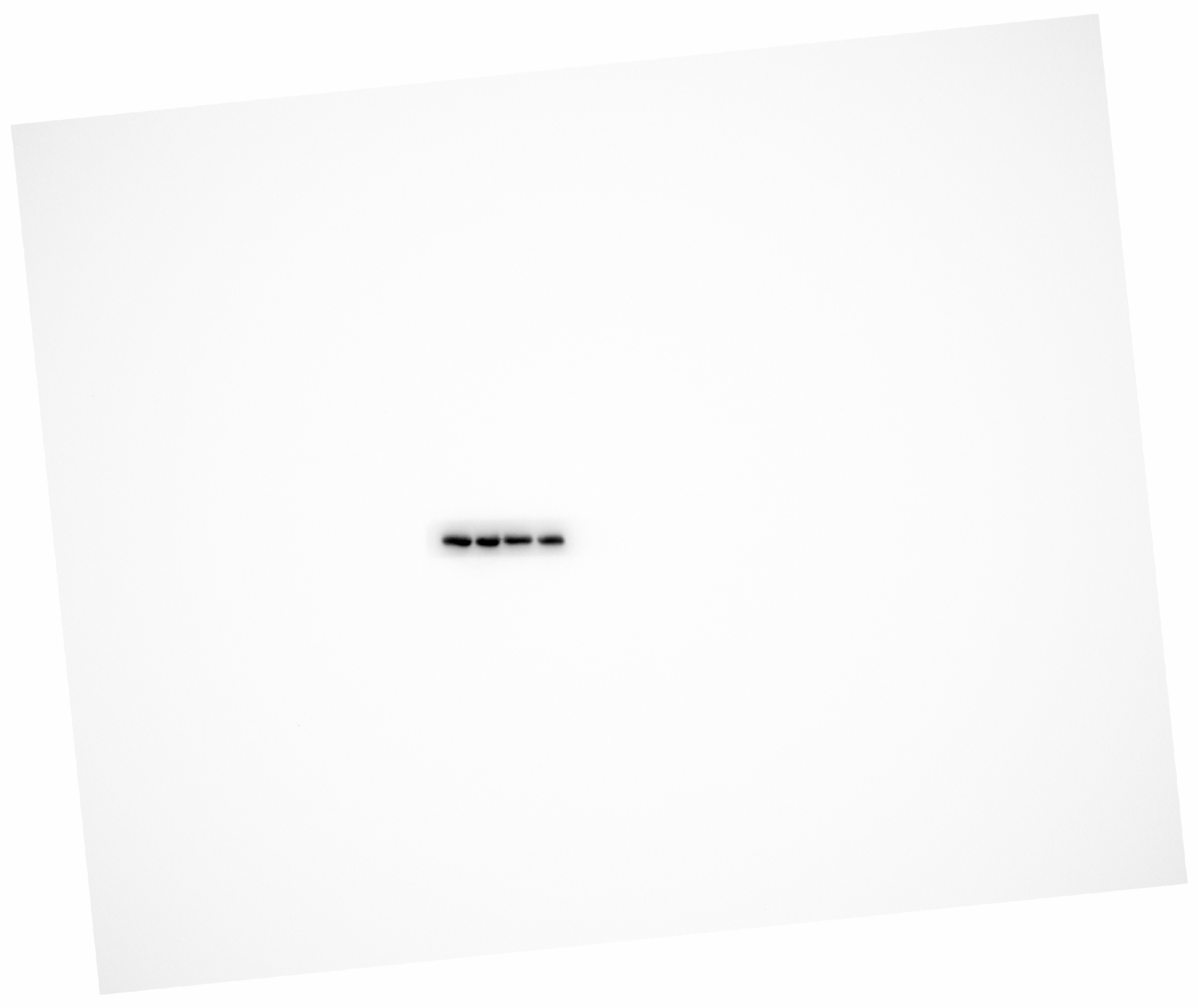

Supplement: Supplementary file 1 — Supplementary Material 1. [file 13046_2026_3724_MOESM1_ESM.zip › WB tiff/GA-parp-bcl2 SUDHL4 SUDHL6.jpg]

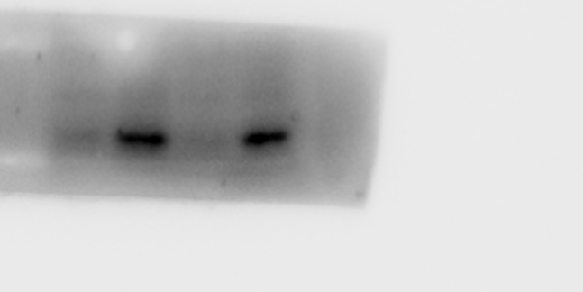

Supplement: Supplementary file 1 — Supplementary Material 1. [file 13046_2026_3724_MOESM1_ESM.zip › WB tiff/GABARAPL1 SUDHL4 SUDHL6.jpg]

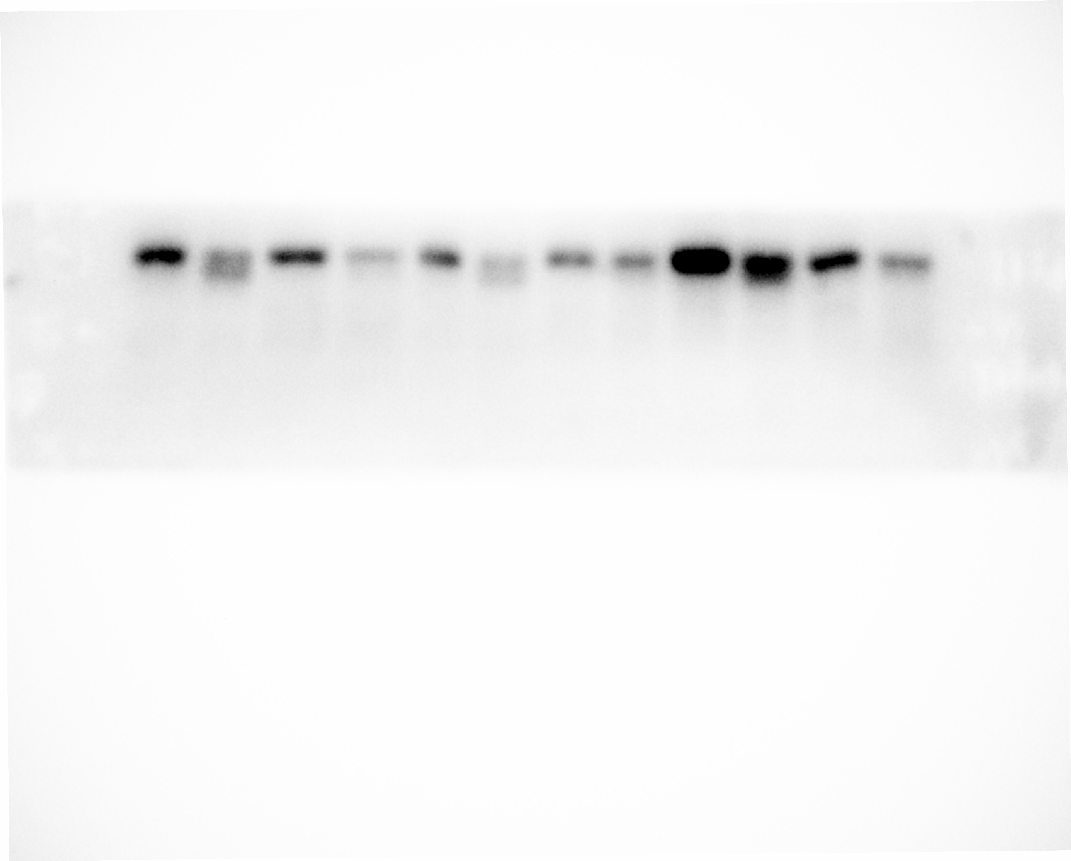

Supplement: Supplementary file 1 — Supplementary Material 1. [file 13046_2026_3724_MOESM1_ESM.zip › WB tiff/IKBa SUDHL4 SUDHL6.jpg]

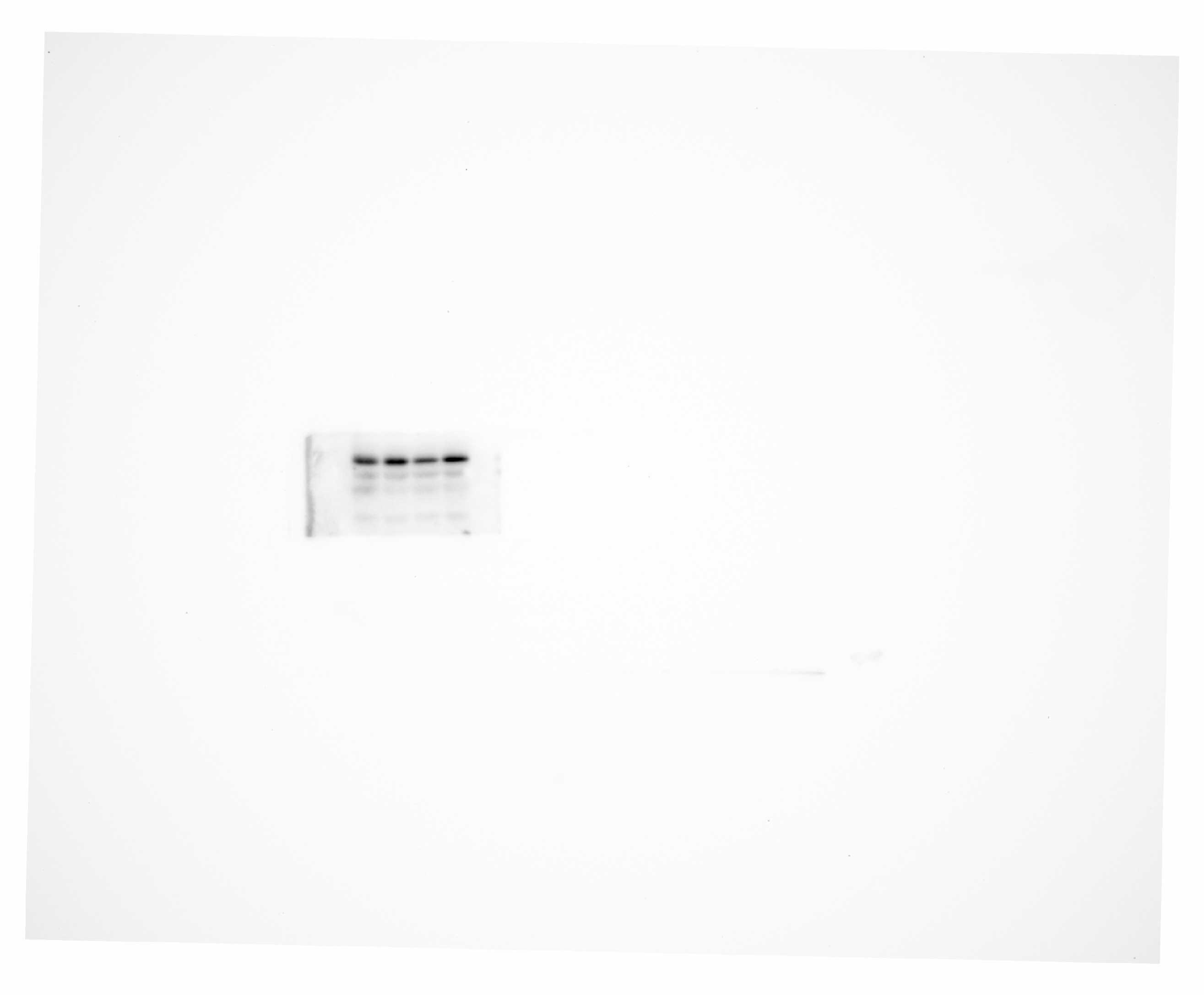

Supplement: Supplementary file 1 — Supplementary Material 1. [file 13046_2026_3724_MOESM1_ESM.zip › WB tiff/IKBa-NAC-SU4.jpg]

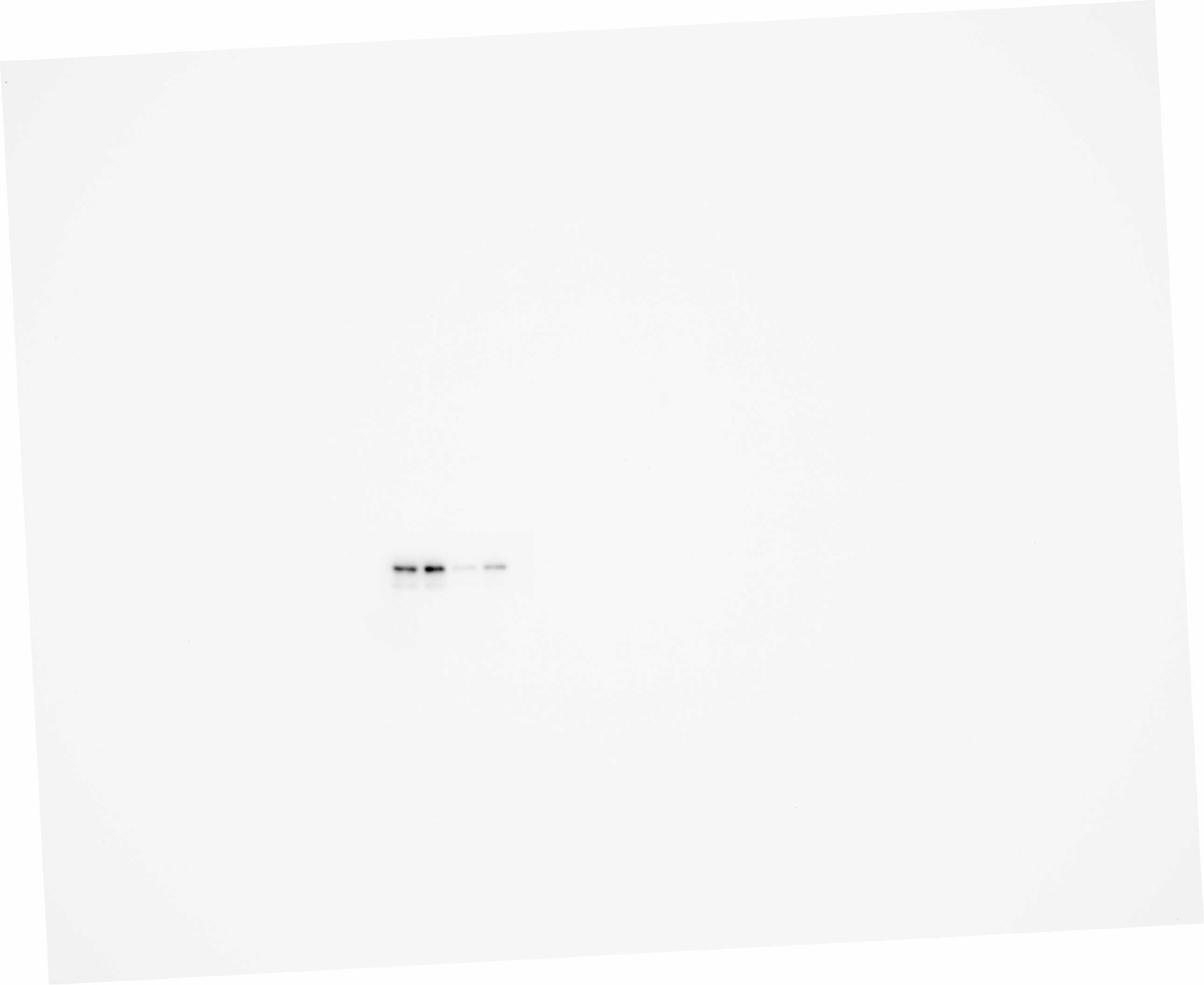

Supplement: Supplementary file 1 — Supplementary Material 1. [file 13046_2026_3724_MOESM1_ESM.zip › WB tiff/IKBa-NAC-U2932.jpg]

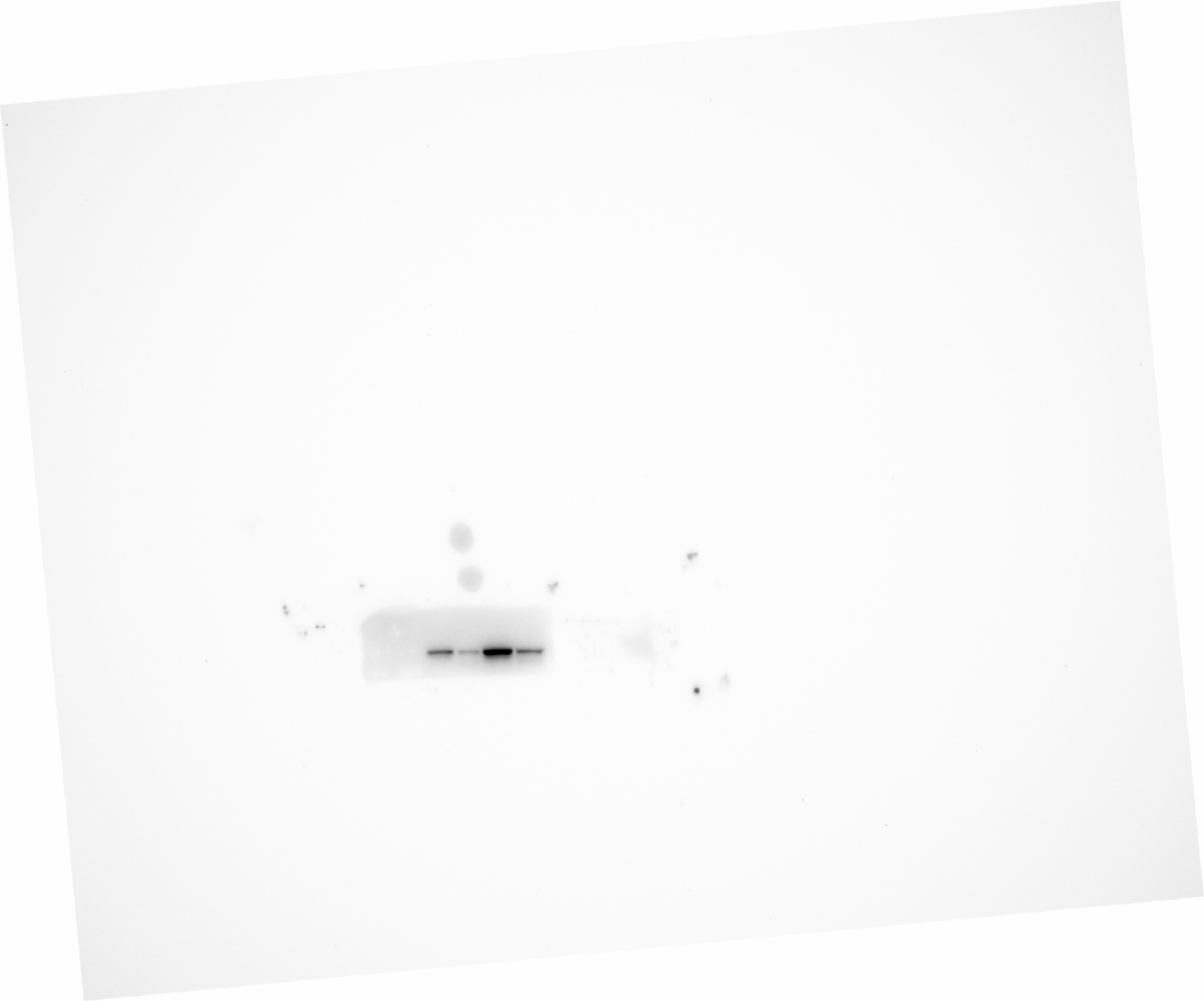

Supplement: Supplementary file 1 — Supplementary Material 1. [file 13046_2026_3724_MOESM1_ESM.zip › WB tiff/IKKa SUDHL4 SUDHL6.jpg]

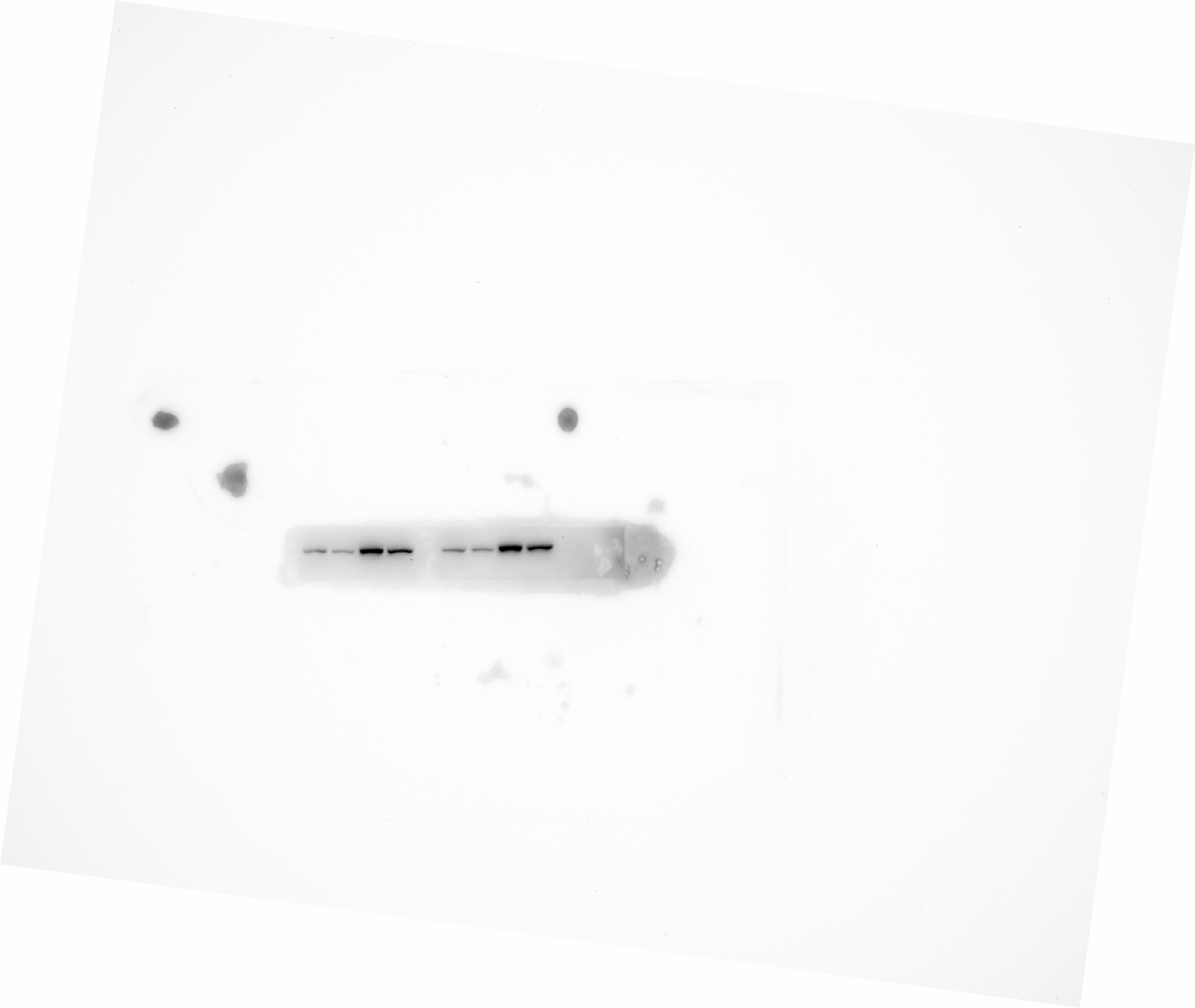

Supplement: Supplementary file 1 — Supplementary Material 1. [file 13046_2026_3724_MOESM1_ESM.zip › WB tiff/IKKb SUDHL4 SUDHL6.jpg]

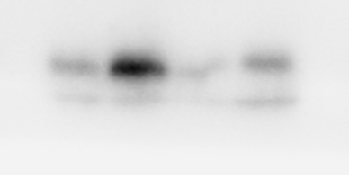

Supplement: Supplementary file 1 — Supplementary Material 1. [file 13046_2026_3724_MOESM1_ESM.zip › WB tiff/Lc3 SUDHL4 SUDHL6.jpg]

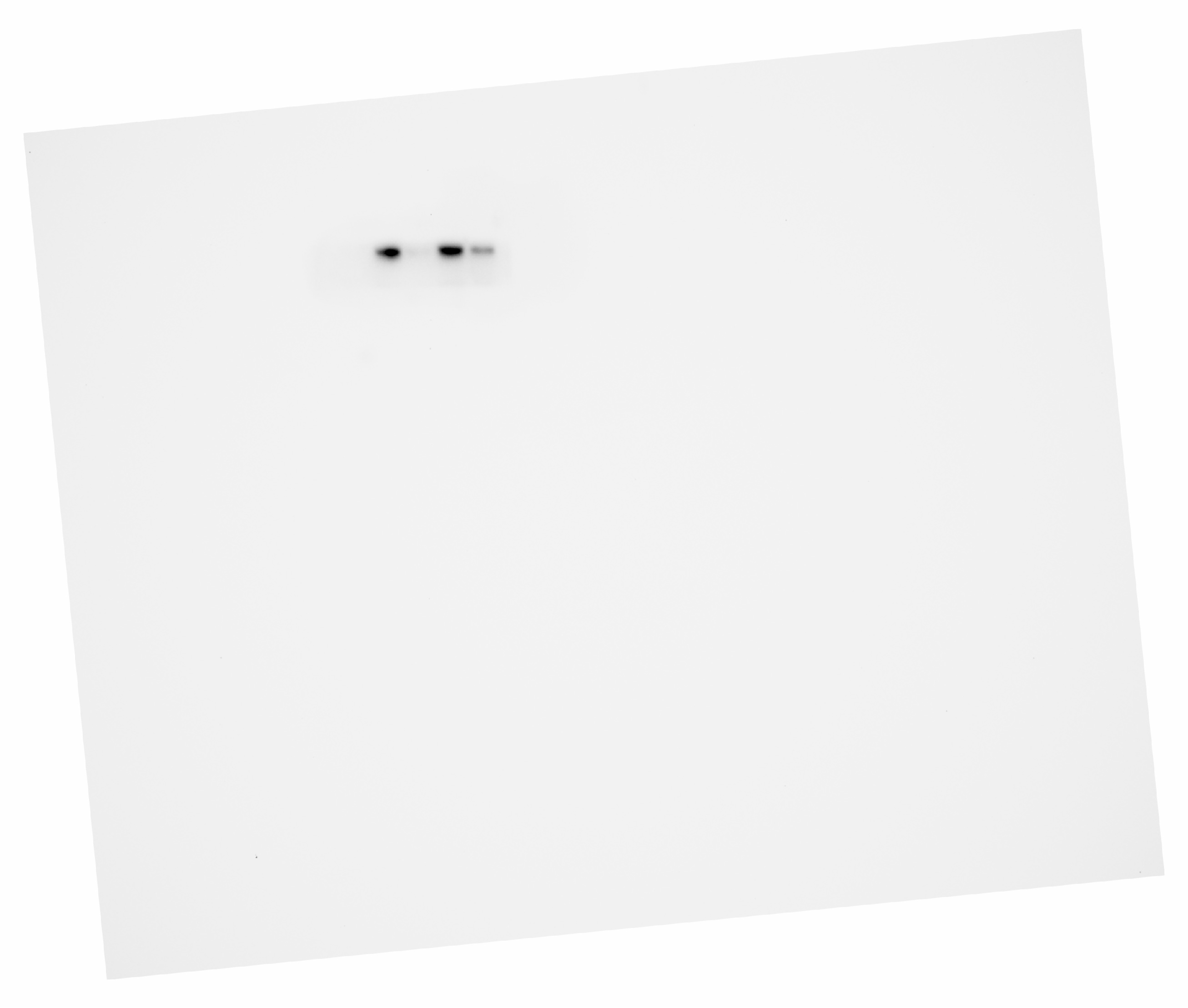

Supplement: Supplementary file 1 — Supplementary Material 1. [file 13046_2026_3724_MOESM1_ESM.zip › WB tiff/Mcl1 SUDHL4 SUDHL6.jpg]

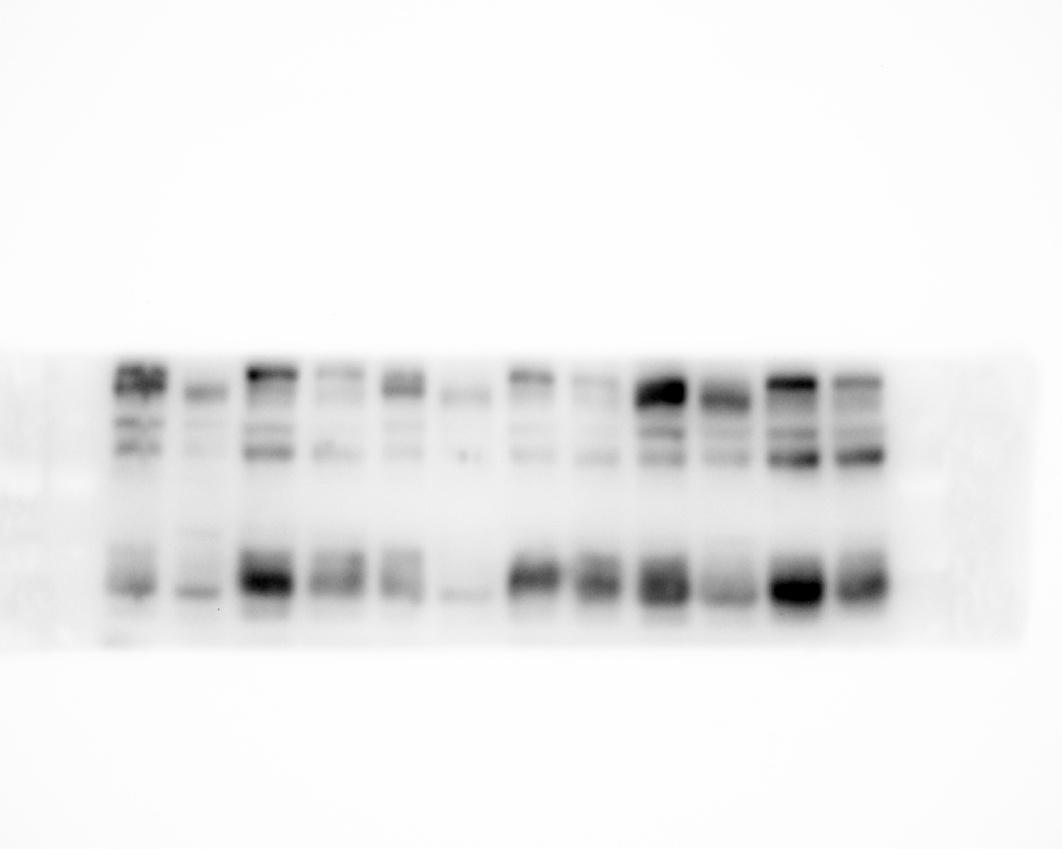

Supplement: Supplementary file 1 — Supplementary Material 1. [file 13046_2026_3724_MOESM1_ESM.zip › WB tiff/p105-p50 SUDHL4 SUDHL6.jpg]

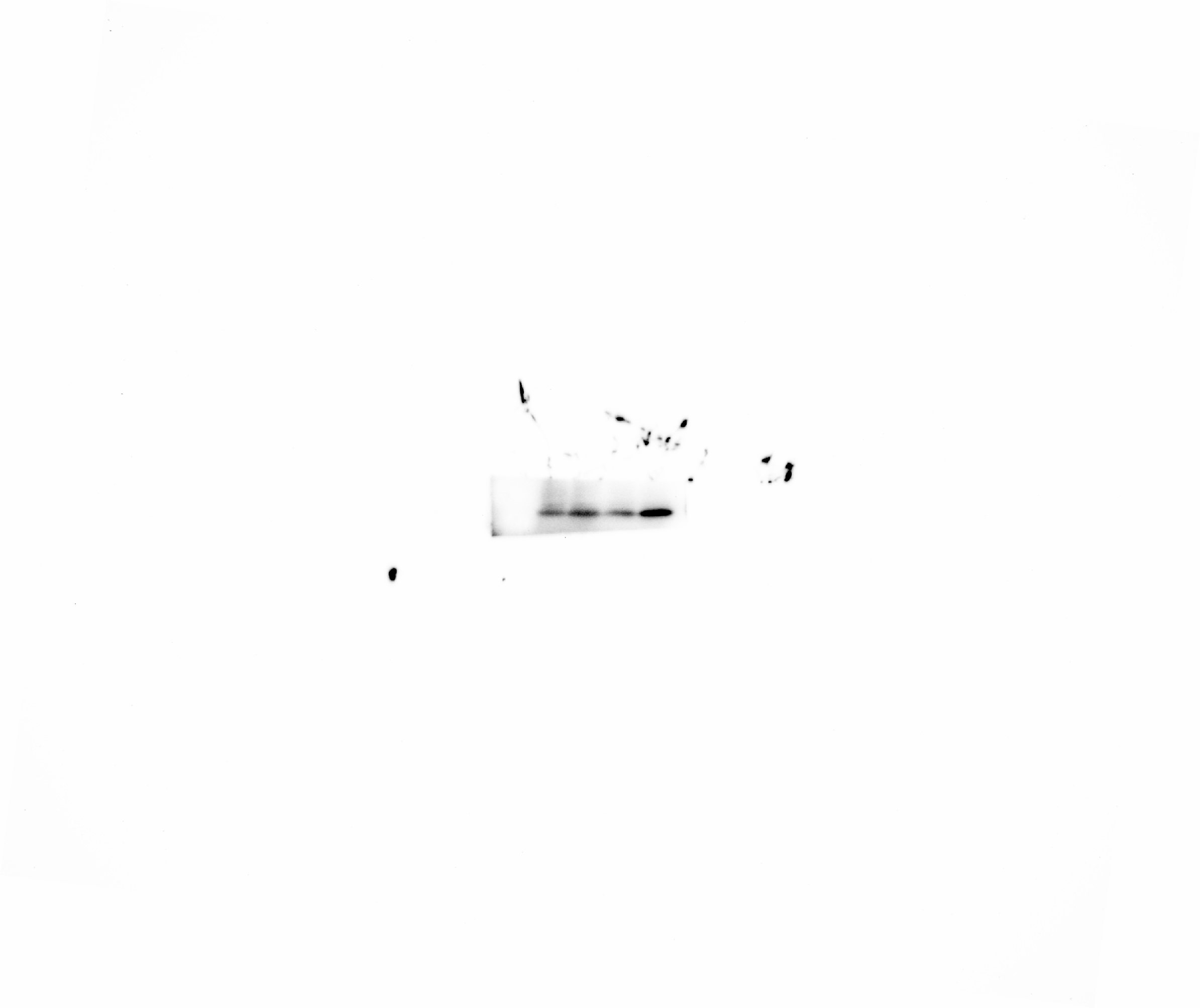

Supplement: Supplementary file 1 — Supplementary Material 1. [file 13046_2026_3724_MOESM1_ESM.zip › WB tiff/P21 SUDHL4 SUDHL6.jpg]

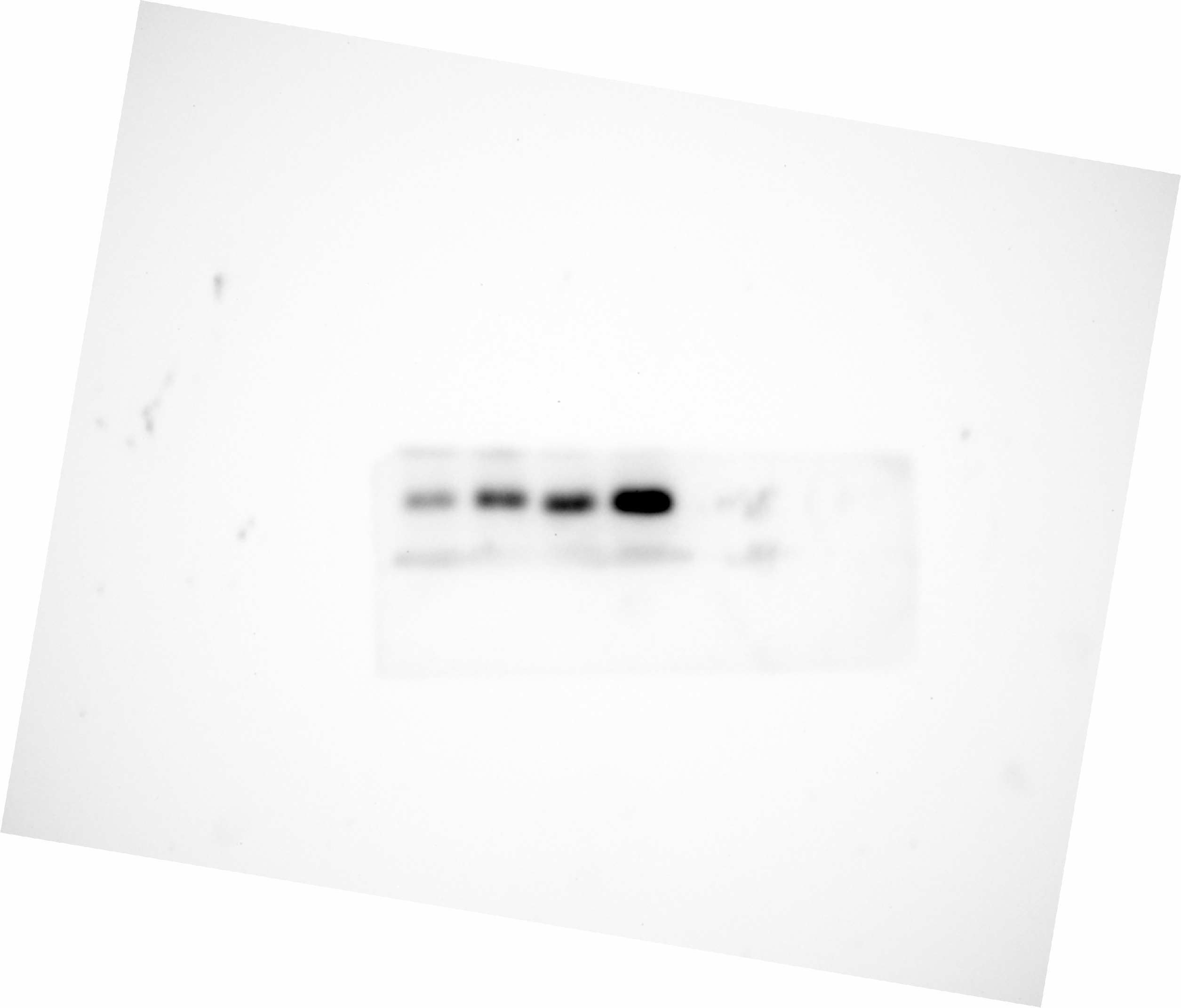

Supplement: Supplementary file 1 — Supplementary Material 1. [file 13046_2026_3724_MOESM1_ESM.zip › WB tiff/P27 SUDHL4 SUDHL6.jpg]

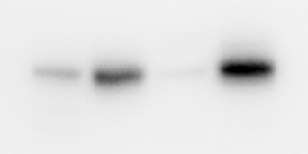

Supplement: Supplementary file 1 — Supplementary Material 1. [file 13046_2026_3724_MOESM1_ESM.zip › WB tiff/p62 SUDHL4 SUDHL6.jpg]

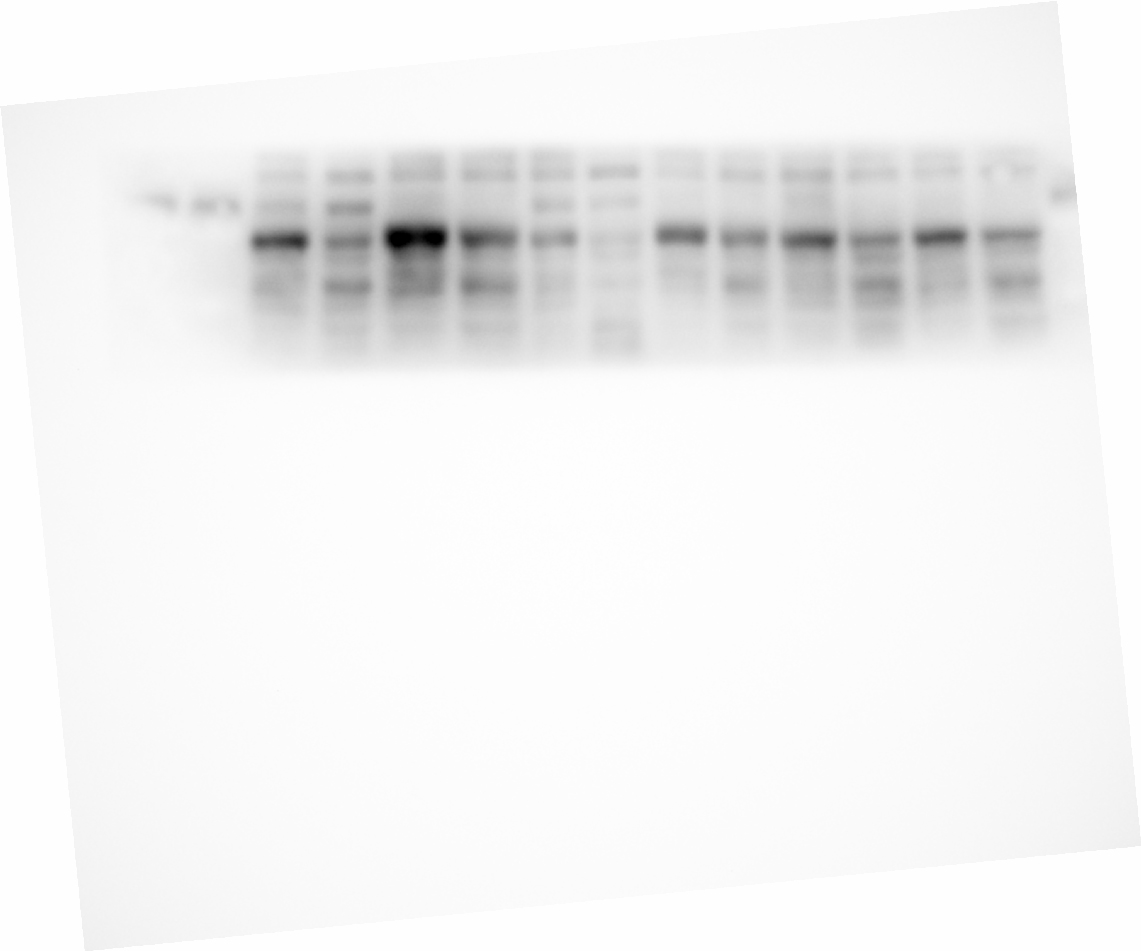

Supplement: Supplementary file 1 — Supplementary Material 1. [file 13046_2026_3724_MOESM1_ESM.zip › WB tiff/P65 SUDHL4 SUDHL6.jpg]

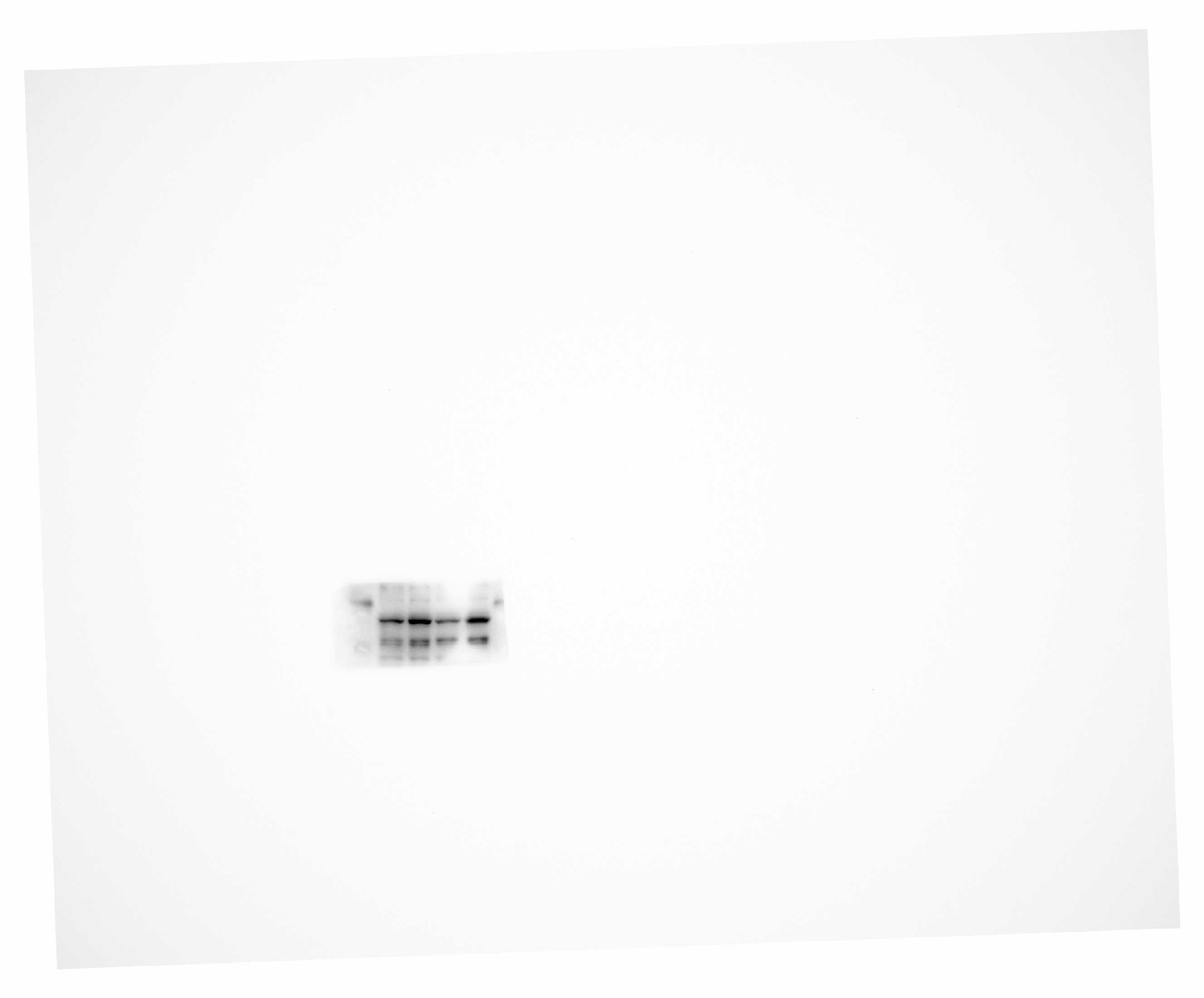

Supplement: Supplementary file 1 — Supplementary Material 1. [file 13046_2026_3724_MOESM1_ESM.zip › WB tiff/p65--NAC,U2932.jpg]

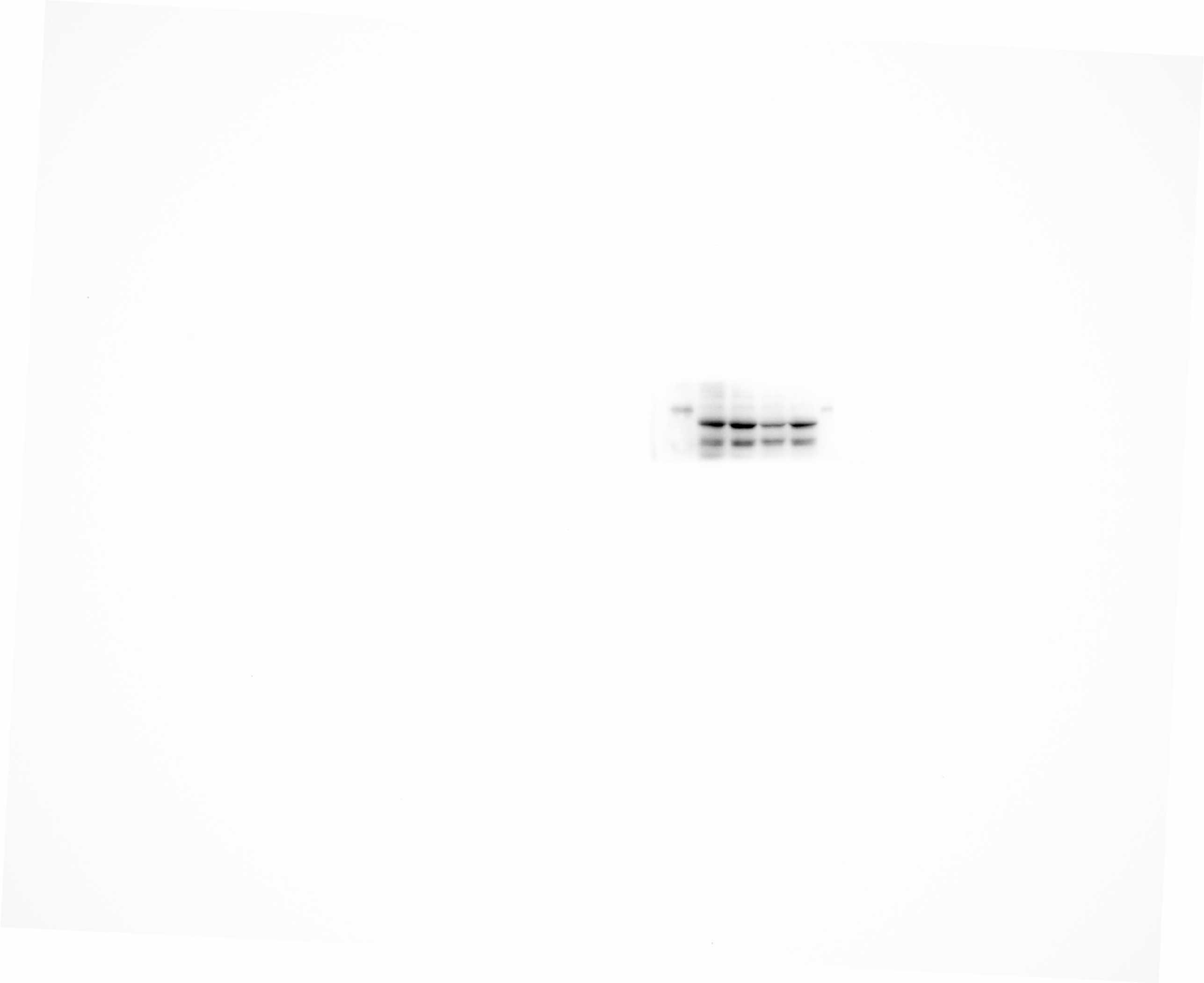

Supplement: Supplementary file 1 — Supplementary Material 1. [file 13046_2026_3724_MOESM1_ESM.zip › WB tiff/p65--NAC-SU4.jpg]

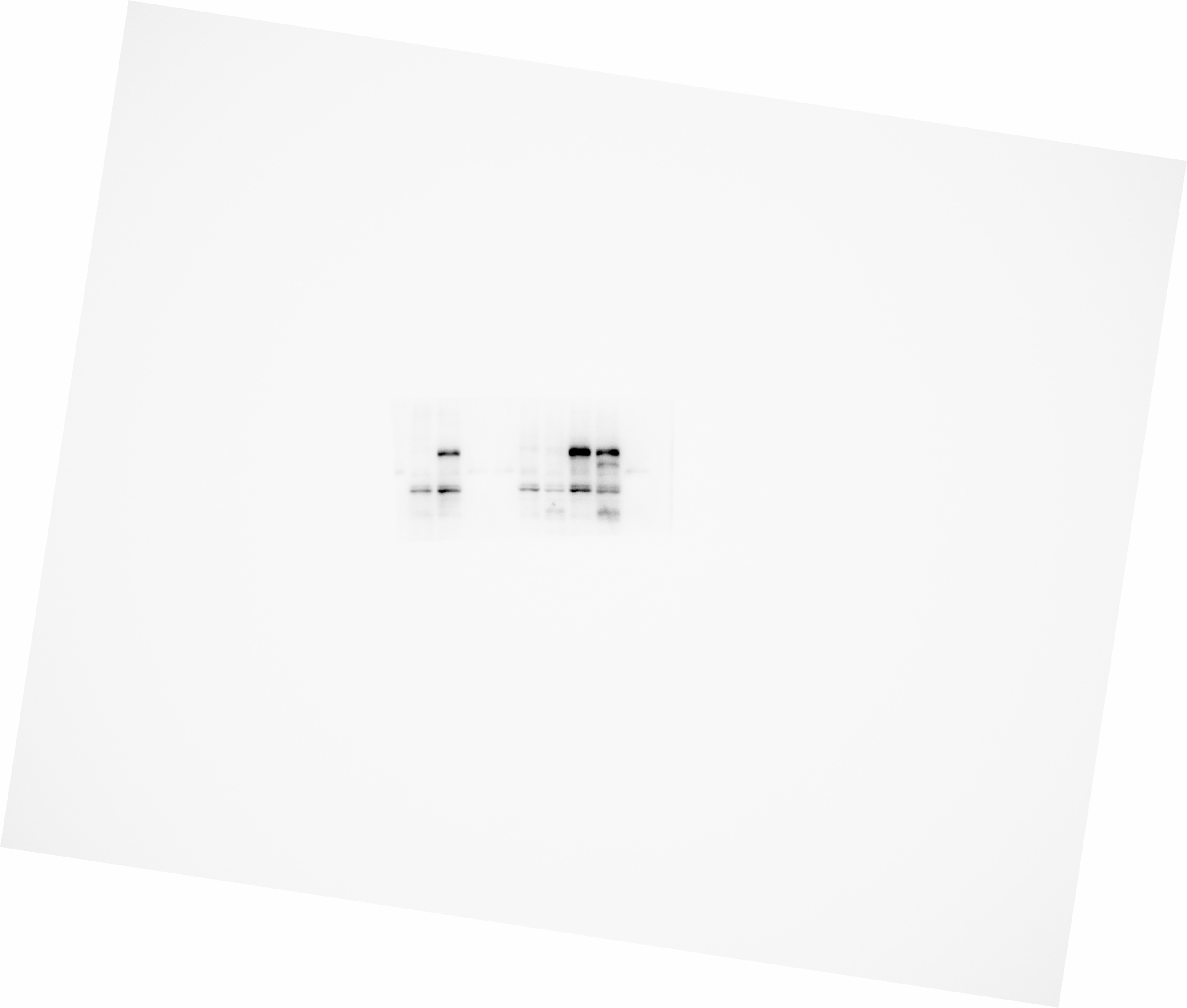

Supplement: Supplementary file 1 — Supplementary Material 1. [file 13046_2026_3724_MOESM1_ESM.zip › WB tiff/p65OE(SU6).jpg]

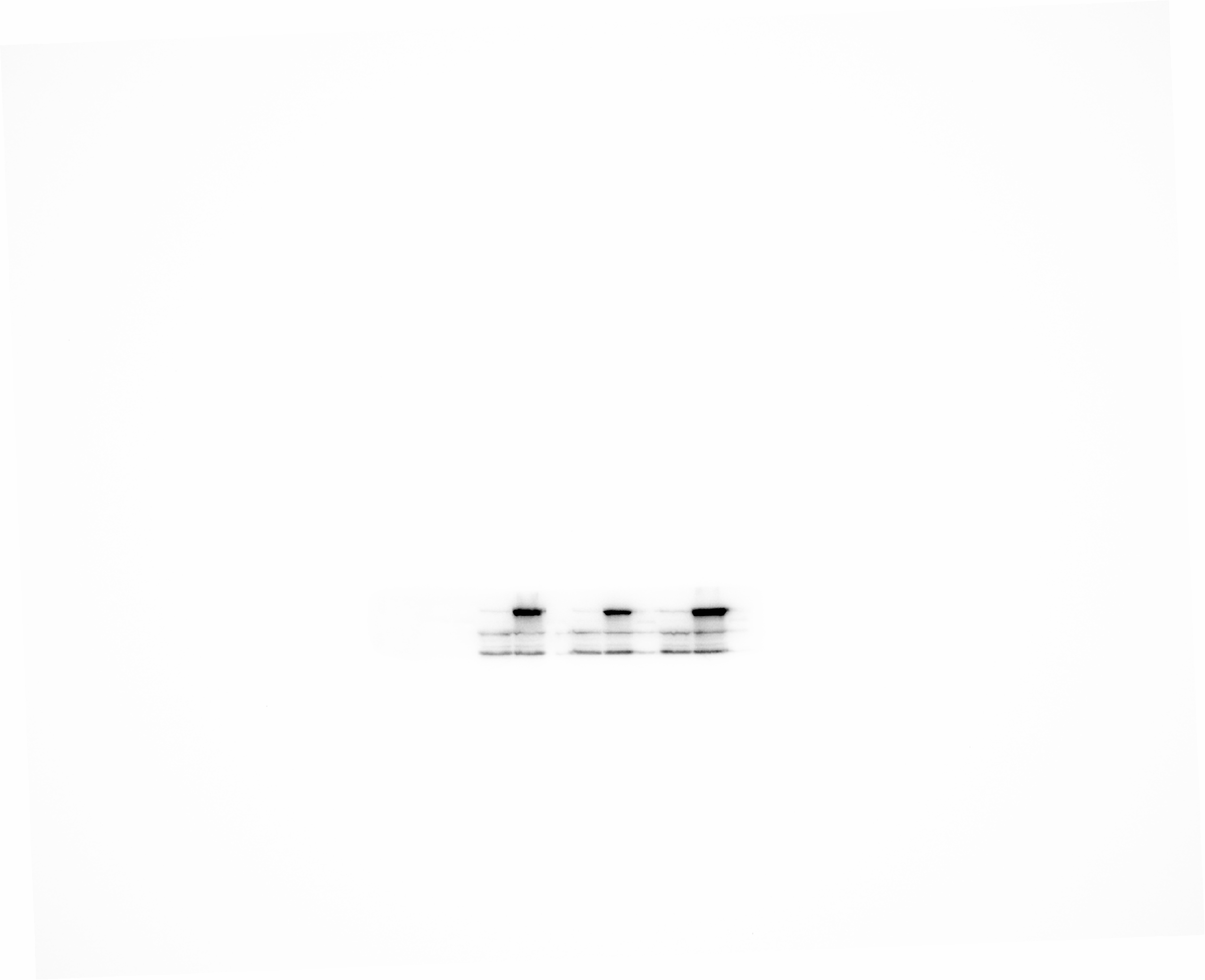

Supplement: Supplementary file 1 — Supplementary Material 1. [file 13046_2026_3724_MOESM1_ESM.zip › WB tiff/p65OE(SU4).jpg]

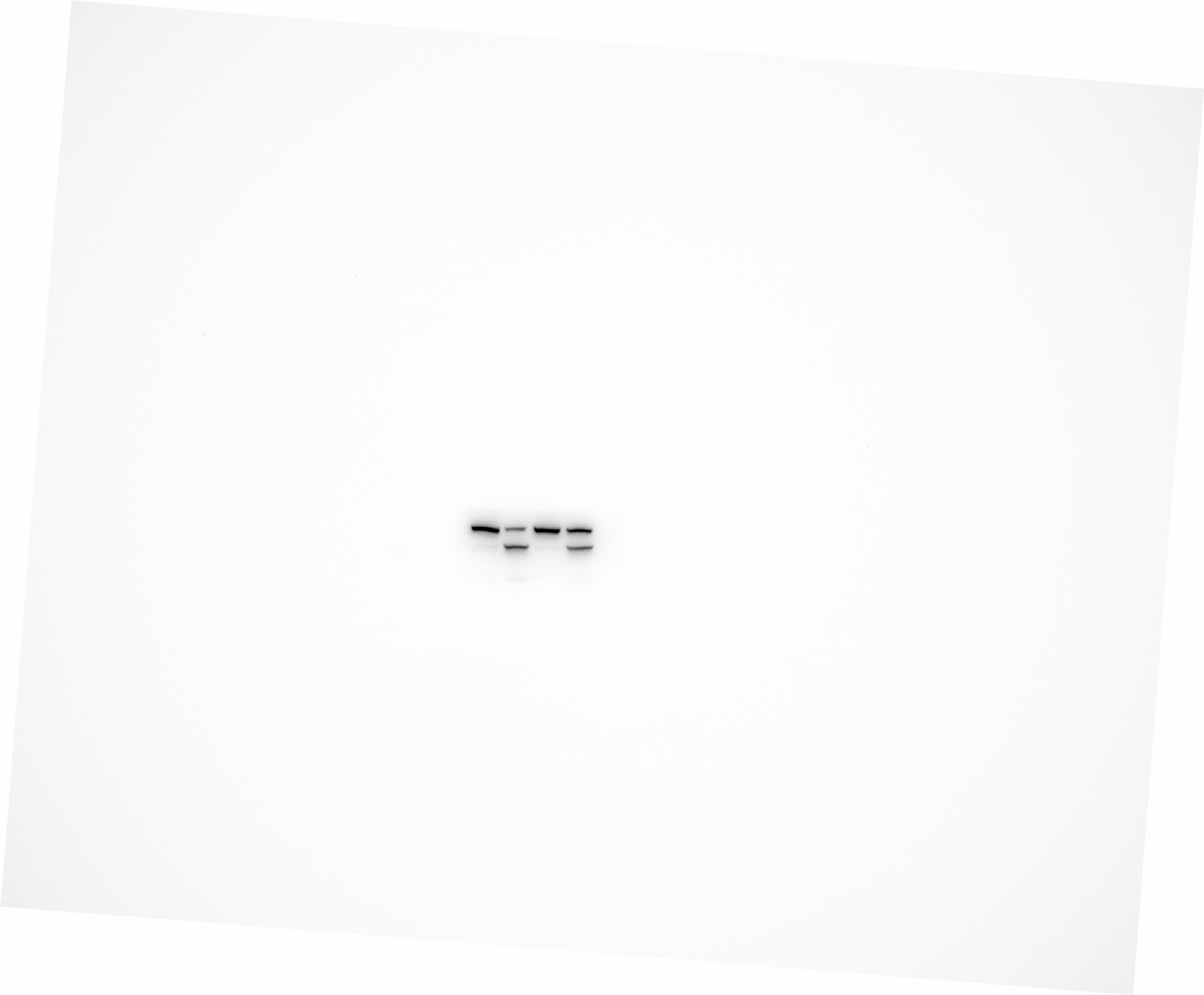

Supplement: Supplementary file 1 — Supplementary Material 1. [file 13046_2026_3724_MOESM1_ESM.zip › WB tiff/PARP SUDHL4 SUDHL6.jpg]

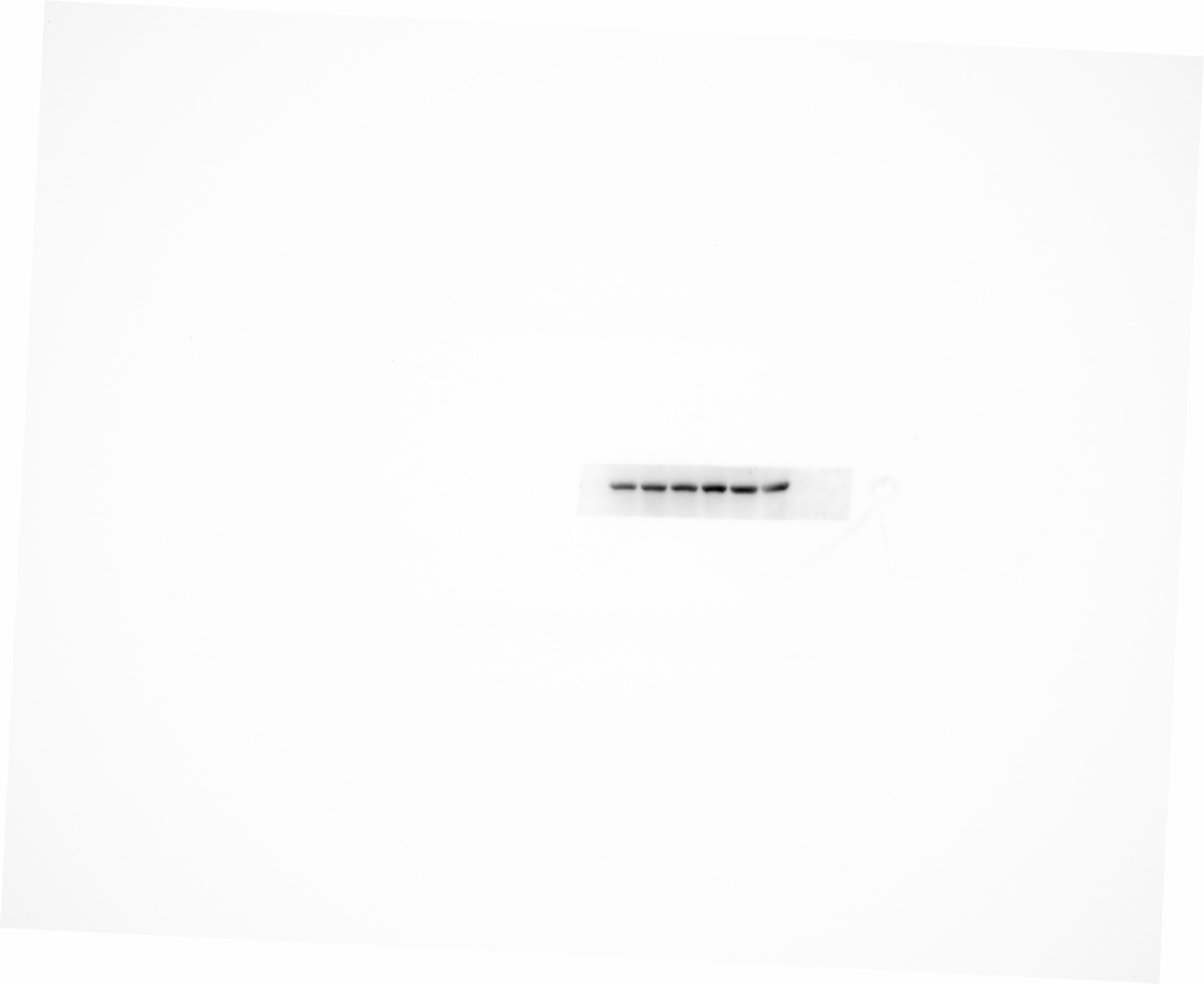

Supplement: Supplementary file 1 — Supplementary Material 1. [file 13046_2026_3724_MOESM1_ESM.zip › WB tiff/SI-P65-GAP-SU4-U2932.jpg]

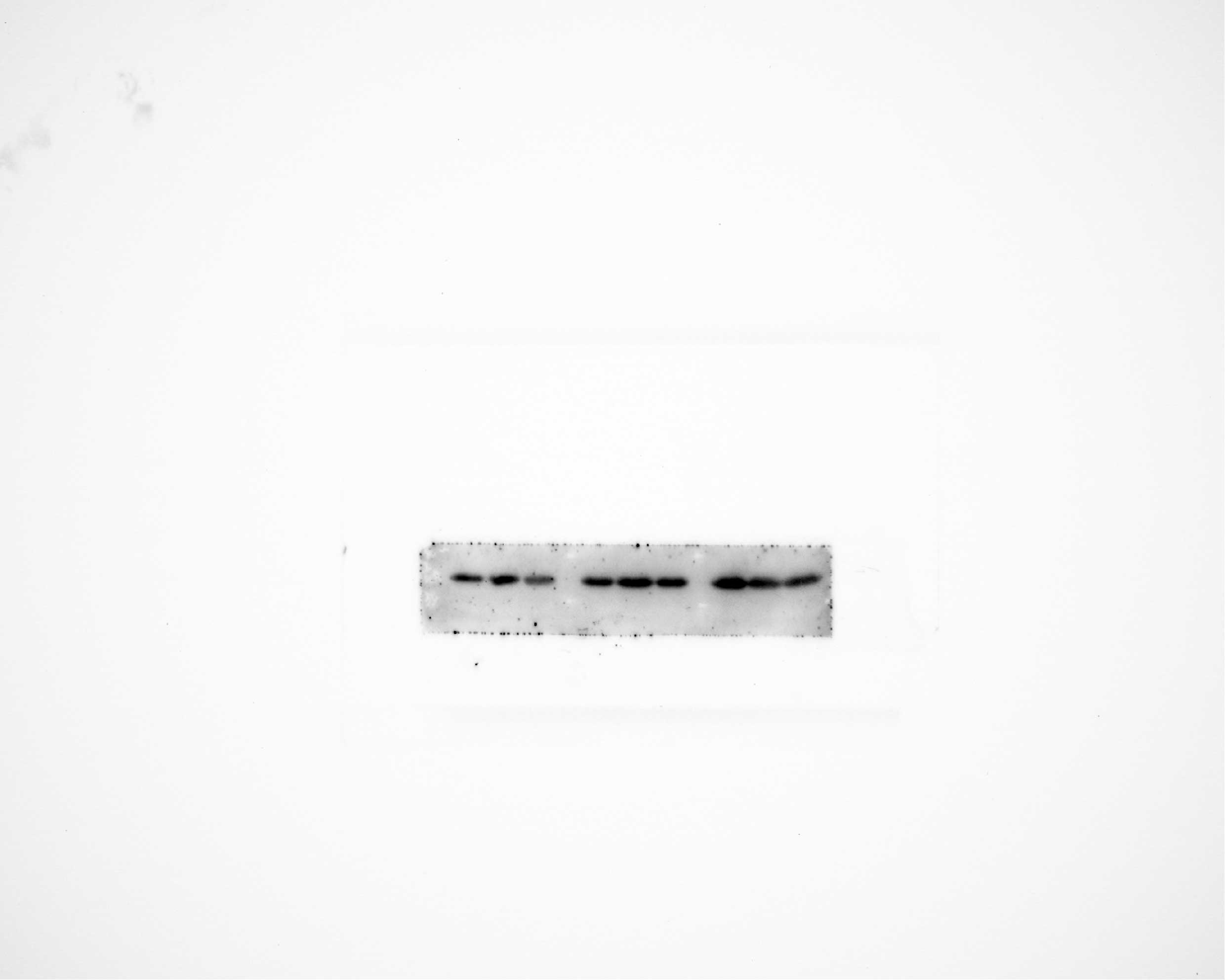

Supplement: Supplementary file 1 — Supplementary Material 1. [file 13046_2026_3724_MOESM1_ESM.zip › WB tiff/SI-P65-GAP-SU6 HBL1.jpg]

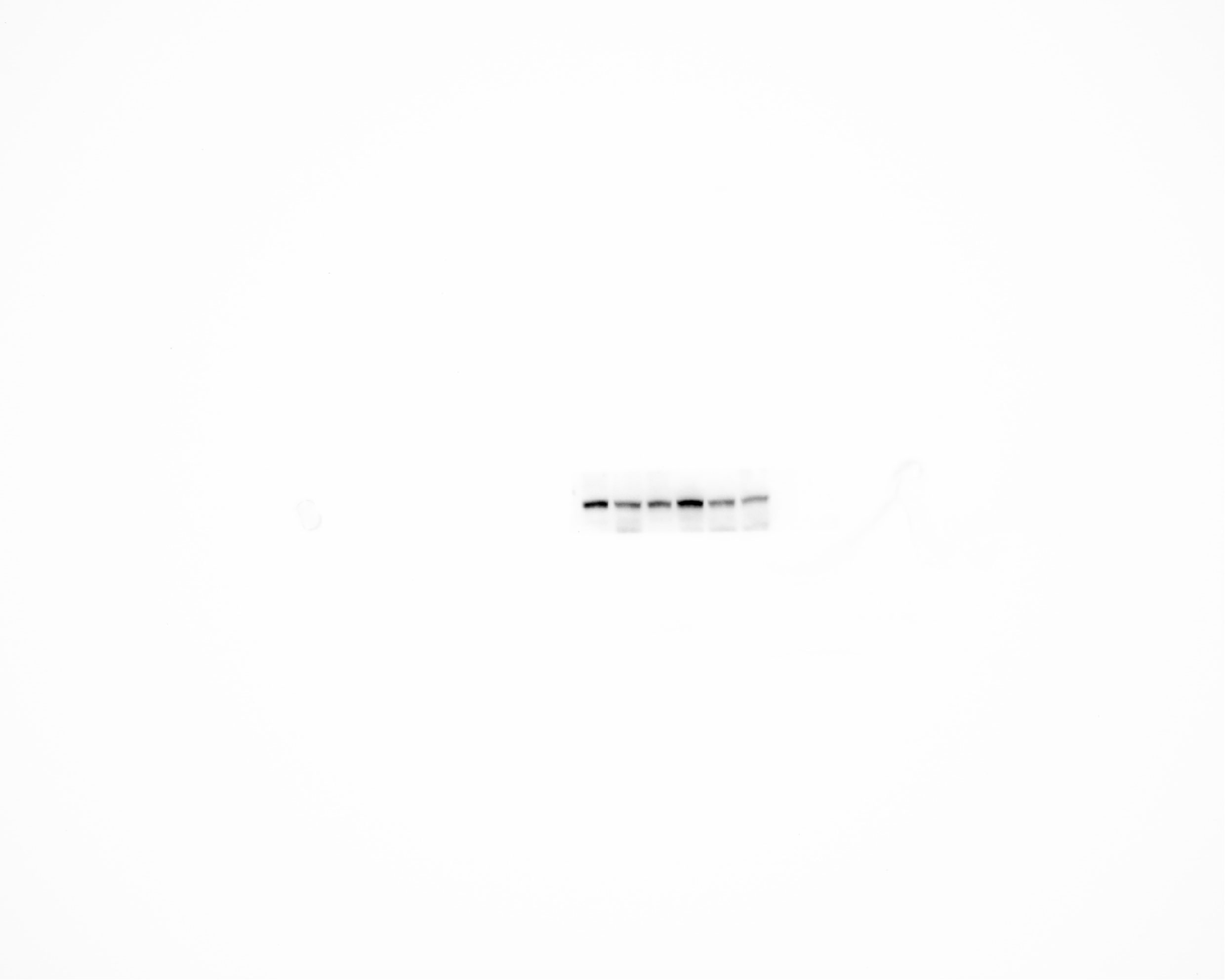

Supplement: Supplementary file 1 — Supplementary Material 1. [file 13046_2026_3724_MOESM1_ESM.zip › WB tiff/SI-P65-SU4U2932.jpg]

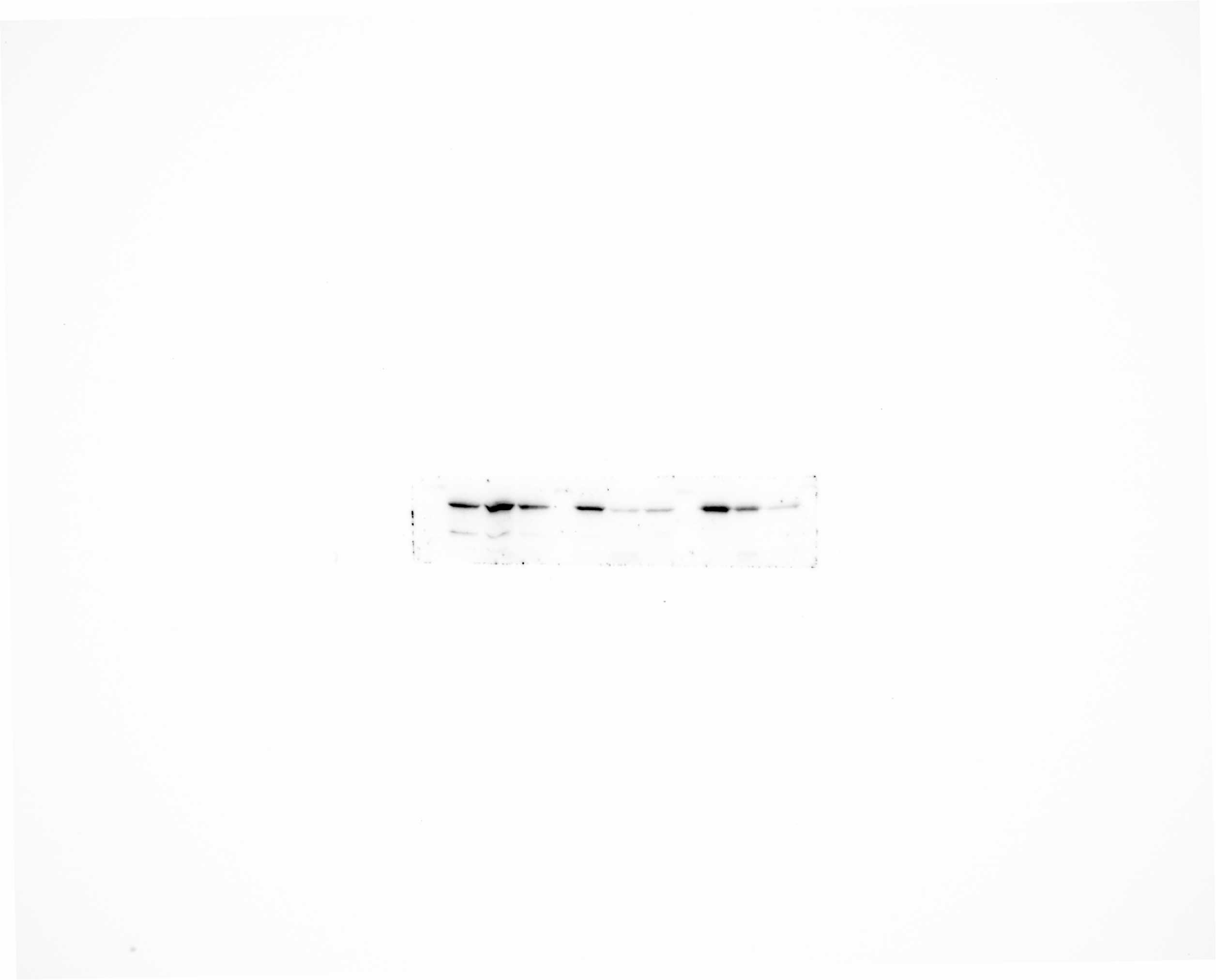

Supplement: Supplementary file 1 — Supplementary Material 1. [file 13046_2026_3724_MOESM1_ESM.zip › WB tiff/SI-P65-SU6 HBL1.jpg]

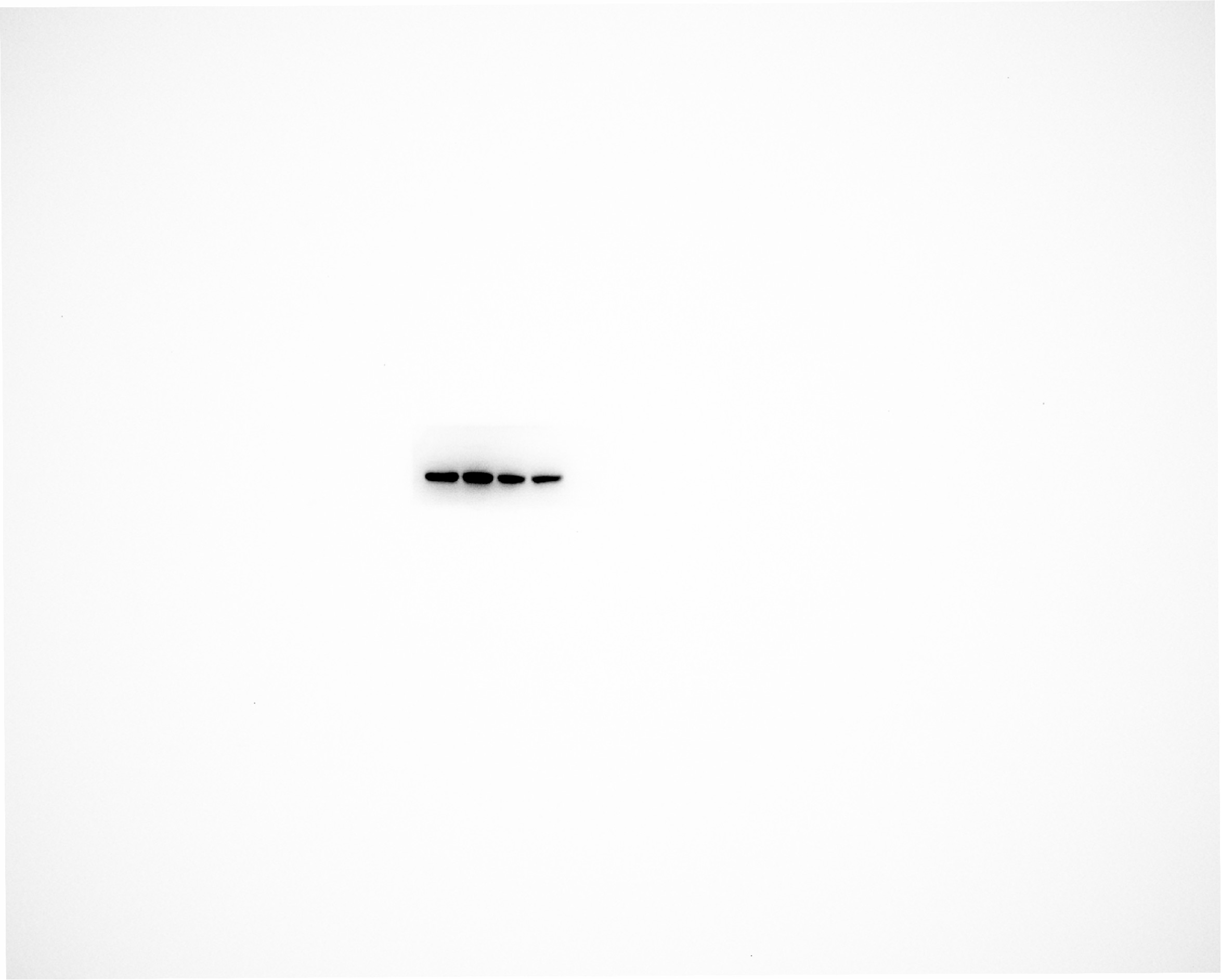

Supplement: Supplementary file 1 — Supplementary Material 1. [file 13046_2026_3724_MOESM1_ESM.zip › WB tiff/Tubulin-CDK6 SUDHL4 SUDHL6.jpg]

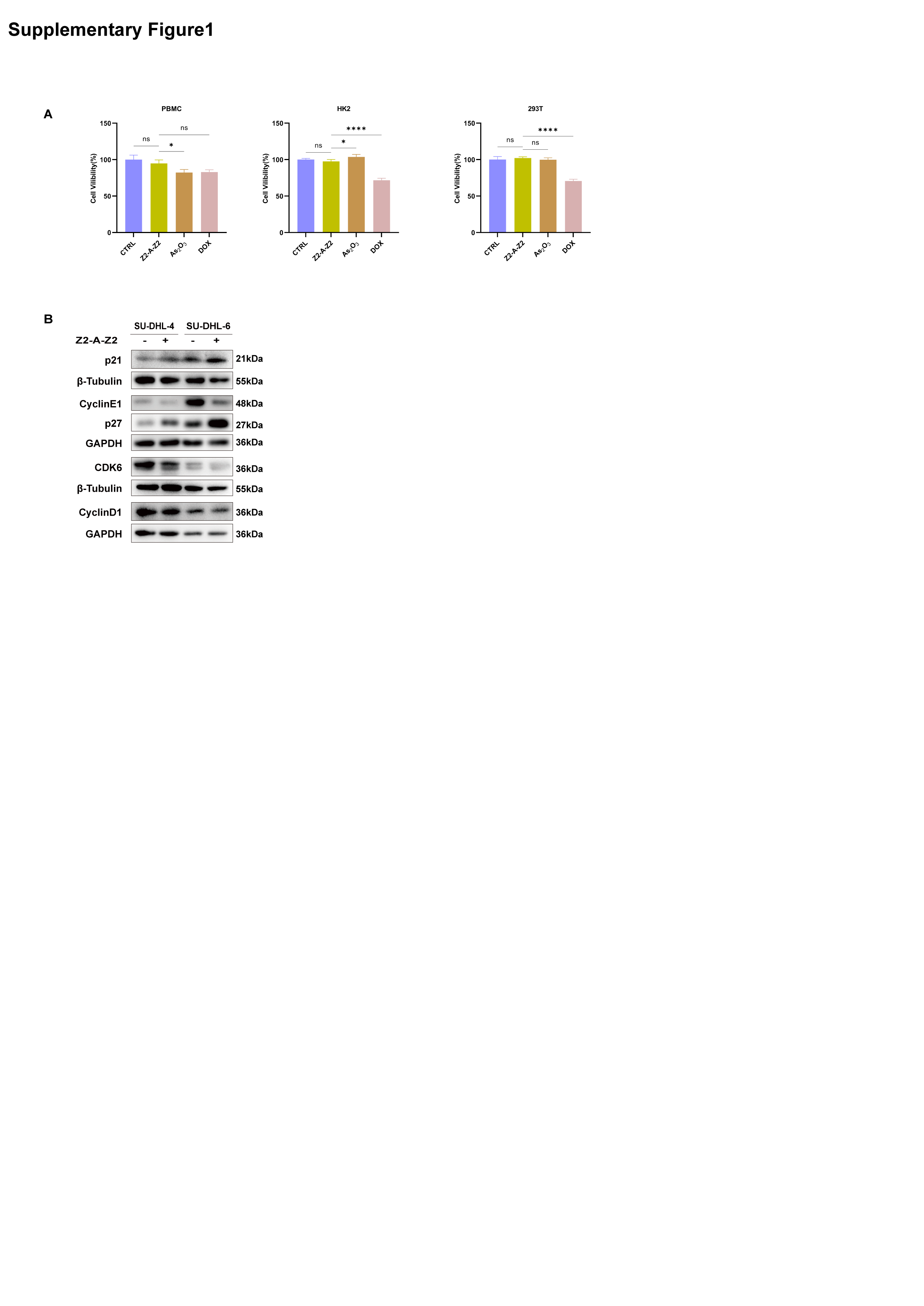

Supplement: Supplementary file 2 — Supplementary Material 2. [file 13046_2026_3724_MOESM2_ESM.zip › Supplementary Figure1.tif]

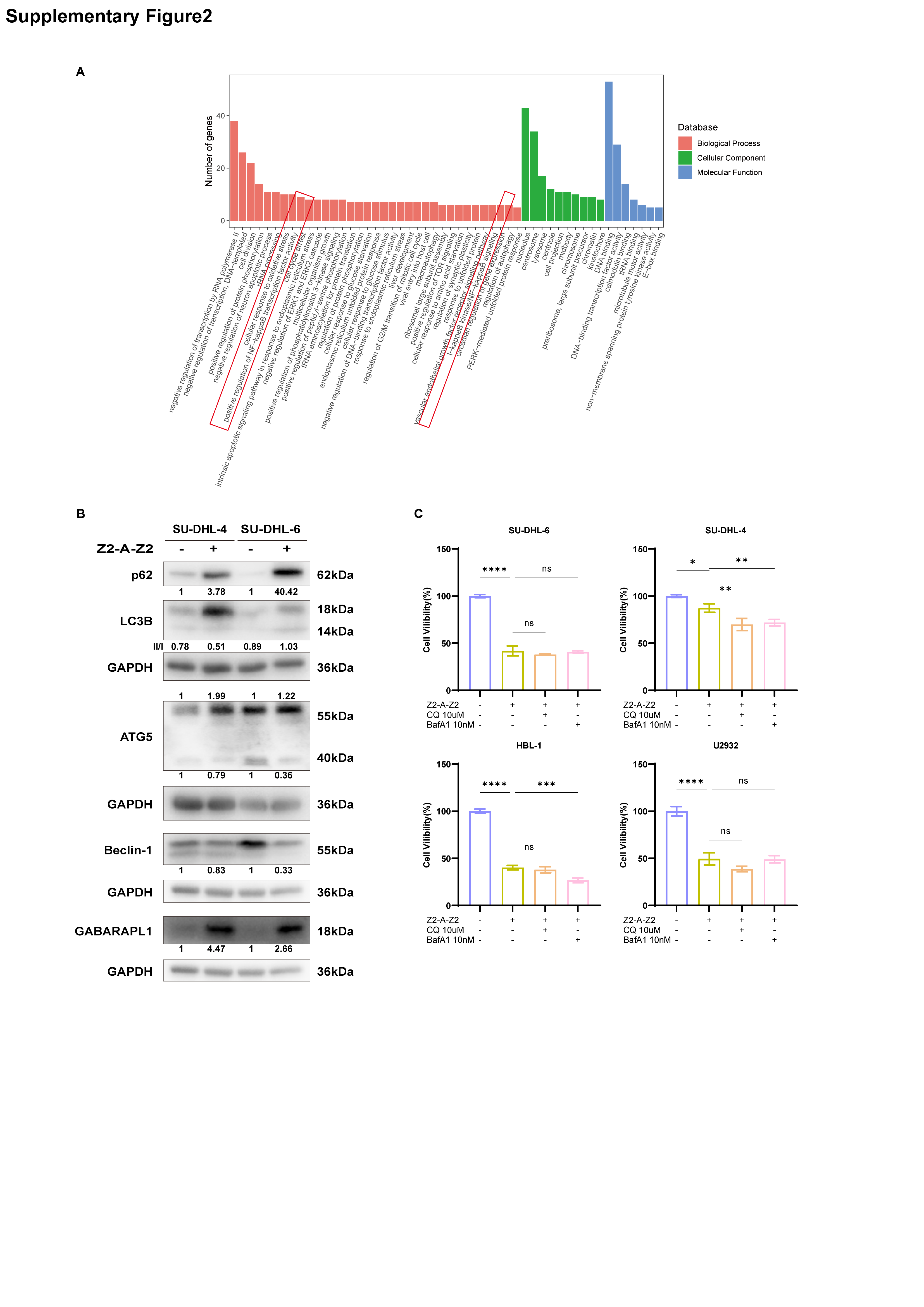

Supplement: Supplementary file 2 — Supplementary Material 2. [file 13046_2026_3724_MOESM2_ESM.zip › Supplementary Figure2.tif]

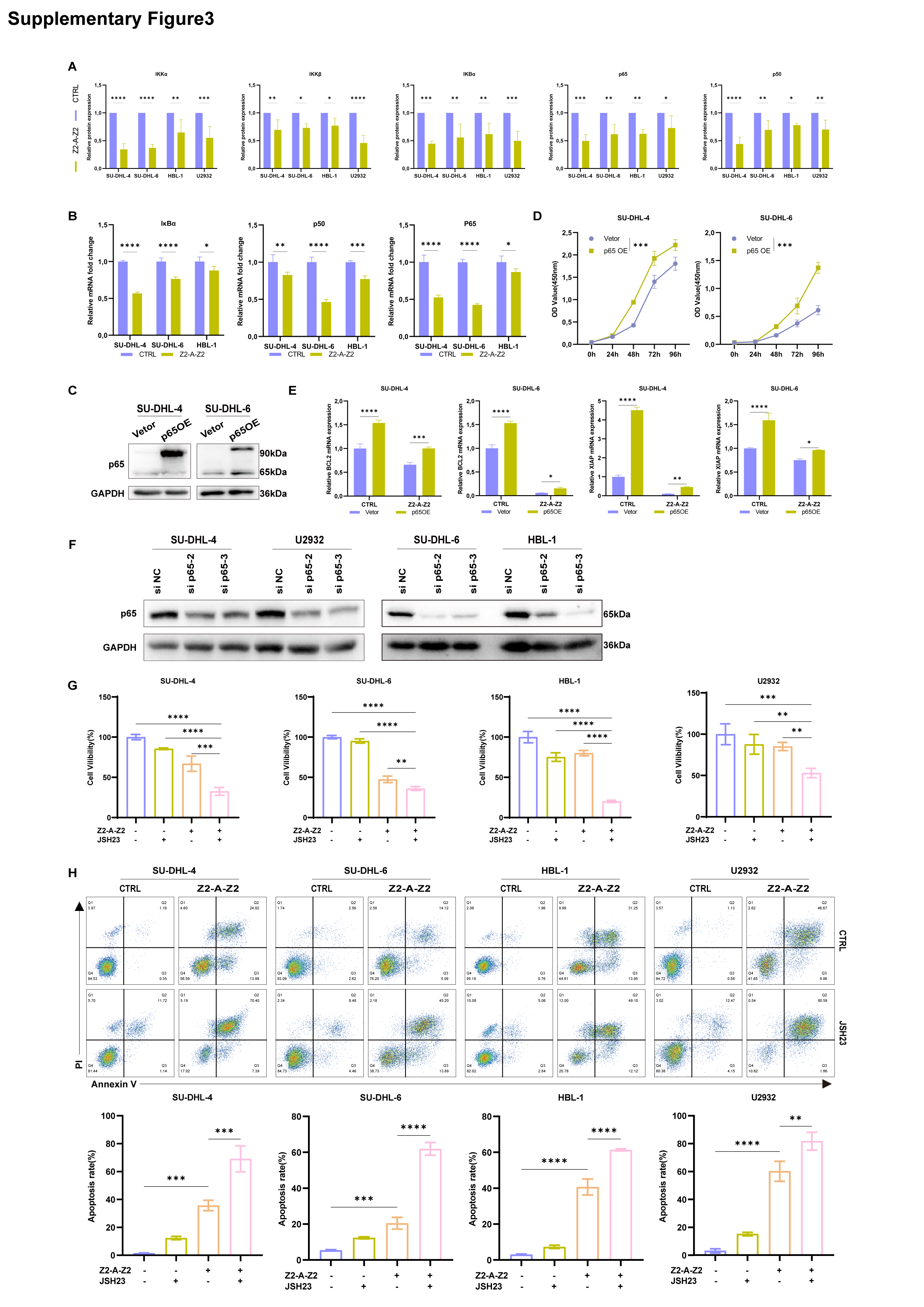

Supplement: Supplementary file 2 — Supplementary Material 2. [file 13046_2026_3724_MOESM2_ESM.zip › Supplementary Figure3.tif]

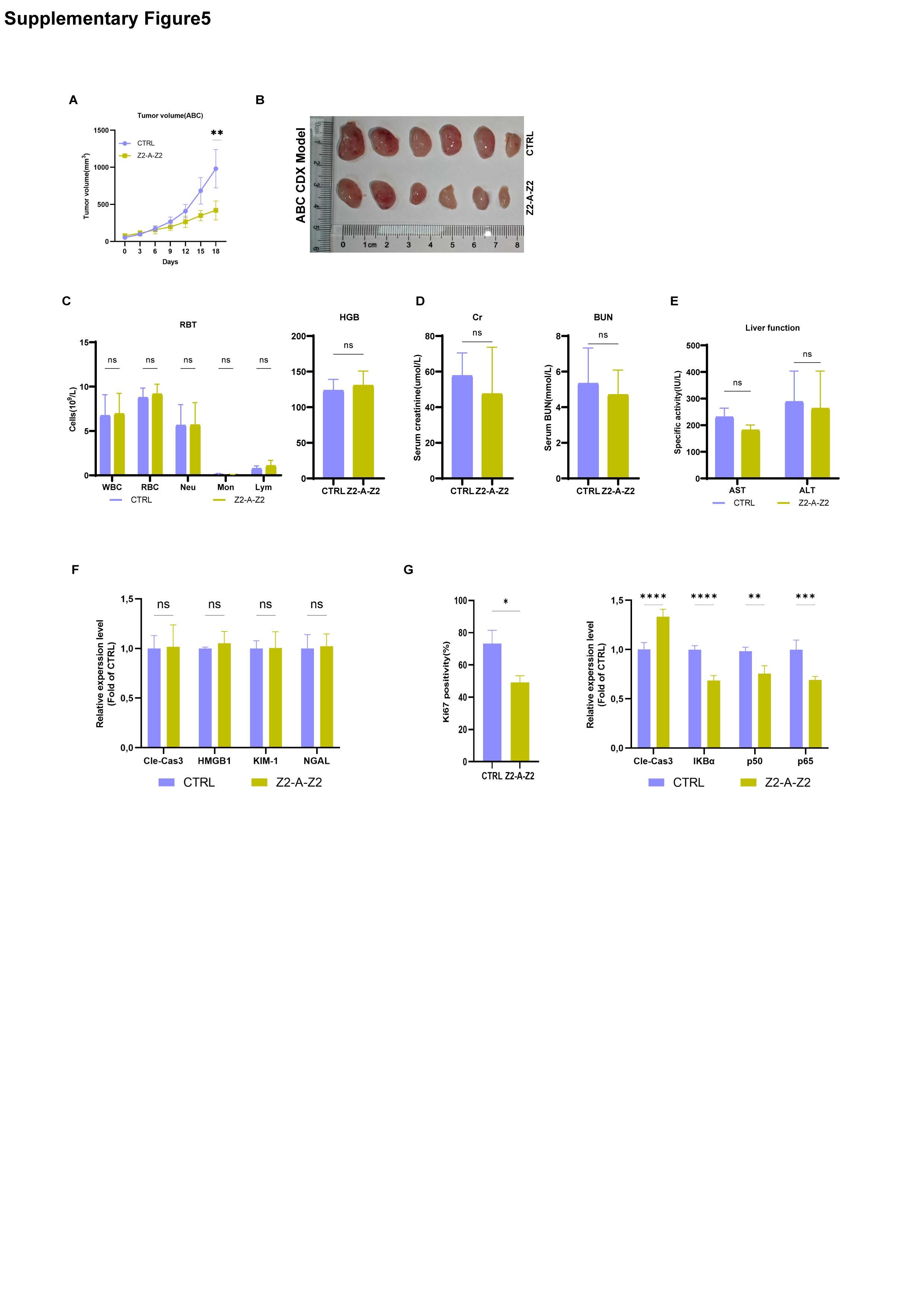

Supplement: Supplementary file 2 — Supplementary Material 2. [file 13046_2026_3724_MOESM2_ESM.zip › Supplementary Figure5.tif]
