# Supplementary material for: Superior anti-DLBCL efficacy of novel organic arsenical Z2-A-Z2 through ROS-mediated apoptosis and critical NF-κB/IκBα signaling pathway inhibition
Source: J Exp Clin Cancer Res. 2026 May 18;45:155. doi: 10.1186/s13046-026-03724-4 (PMC13352683; doi:10.1186/s13046-026-03724-4)
Supplement: Supplementary file 3 — Supplementary Material 3. [file 13046_2026_3724_MOESM3_ESM.docx]

**Supplementary Figure 1. Extended safety profile and molecular markers of cell cycle arrest induced by Z2-A-Z2.** **(A)** Evaluation of systemic and renal toxicity. Viability of primary human B cells, T cells, monocytes, and PBMCs, alongside human renal-derived cell lines (HK-2 and HEK293T), was assessed following 24-h exposure to 1 µM Z2-A-Z2 or clinical comparator drugs. Data demonstrate the superior selectivity of Z2-A-Z2 toward malignant cells over normal counterparts. **(B)** Western blot analysis of key cell cycle regulatory proteins in SU-DHL-6 and U2932 cells after 24 h treatment with 1 µM Z2-A-Z2. Results corroborate the induction of cell cycle arrest at the protein level, characterized by the modulation of CKD6, Cyclin D1, p21, and p27. Data are presented as mean ± SD from three independent experiments (n=3).

**Supplementary Figure 2. Functional enrichment analysis of differentially expressed genes and characterization of Z2-A-Z2-induced autophagy.** **(A)** Gene Ontology (GO) enrichment analysis of differentially expressed genes (DEGs) in DLBCL cells following Z2-A-Z2 treatment. Bar graphs illustrate the significantly enriched terms across Biological Process (BP), Cellular Component (CC), and Molecular Function (MF) categories, highlighting the modulation of [Insert most relevant term, e.g., redox homeostasis or apoptotic signaling]. **(B)** Western blot analysis of autophagy-related markers, including p62, ATG5, GABARAPL1, and Beclin-1. **(C)** Functional assessment of autophagy in Z2-A-Z2-mediated cytotoxicity. DLBCL cells were pretreated with the late-stage autophagy inhibitors Bafilomycin A1 (BafA1, 10 nM) or Chloroquine (CQ, 10 μM) for 2 h, followed by co-treatment with Z2-A-Z2 at the indicated concentrations for 24 h. Cell viability was determined by CCK-8 assay, demonstrating that pharmacological inhibition of autophagy does not significantly rescue Z2-A-Z2-induced cell death. Data are presented as mean ± SD from three independent experiments (n=3).

**Supplementary Figure 3. Genetic and pharmacological modulation of p65 underscores its role in Z2-A-Z2-mediated cytotoxicity.** **(A)** Densitometric quantification of Western blot data from **Fig. 5C**. Protein expression levels are normalized to GAPDH and expressed as fold change relative to the vehicle control group. **(B)** qRT-PCR analysis of key NF-κB pathway components in DLBCL cells following 24-h treatment with Z2-A-Z2, reflecting transcriptional suppression of the signaling axis. **(C)** Validation of p65 stable overexpression. Western blot analysis confirming the successful establishment of p65-overexpressing (p65 OE) stable cell lines in SU-DHL-6 and U2932 backgrounds. **(D)** Enhanced proliferative capacity in p65-overexpressing cells. Growth curves of empty vector control and p65 OE cells were monitored over five consecutive days using the CCK-8 assay. **(E)** qRT-PCR analysis of anti-apoptotic genes (*BCL2*, *XIAP*) in vector-control and p65 OE cells post-treatment with Z2-A-Z2. **(F)** Efficiency of siRNA-mediated knockdown. Western blot analysis of p65 expression in DLBCL cells 72 h post-transfection with non-targeting control siRNA (si-NC) or p65-targeting siRNA (si-p65). GAPDH was used as the loading control. **(G, H)** Synergistic effects of p65 inhibition and Z2-A-Z2. Cell viability **(G)** and apoptosis **(H)** were assessed in DLBCL cells treated with the p65 inhibitor JSH-23, Z2-A-Z2, or their combination, highlighting the potency of co-targeting NF-κB. Data are presented as mean ± SD from three independent experiments (n=3).

**Supplementary Figure 4. ROS acts as a critical upstream mediator of mitochondrial dysfunction and NF-κB suppression.** **(A–E)** Functional rescue of Z2-A-Z2-induced cellular damage by ROS scavenging. DLBCL cells were pretreated with or without the antioxidant N-acetylcysteine (NAC, 5 mM) for 1 h, followed by exposure to 1 µM Z2-A-Z2 for 24 h. **(A, B)** Flow cytometric quantification of mitochondrial superoxide (MitoSOX) **(A)** and total intracellular ROS (DCFH-DA) **(B)**, confirming the efficient neutralization of oxidative stress by NAC. **(C)** Assessment of mitochondrial membrane potential via JC-1 staining; NAC pretreatment significantly restored ΔΨm following drug exposure. **(D)** Quantification of the apoptotic cell population by Annexin V/PI dual staining and flow cytometry, showing that ROS scavenging abrogates Z2-A-Z2-mediated cell death. **(E)** Representative immunofluorescence images of phospho-p65 (p-p65), demonstrating that ROS inhibition rescues p65 phosphorylation**. (F)** Potentiation of oxidative stress by NF-κB activation. DLBCL cells were pretreated with or without the NF-κB activator lipopolysaccharide (LPS, 10 ng/µL) for 1 h prior to Z2-A-Z2 treatment. Quantification of mitochondrial superoxide levels reveals that NF-κB activation exacerbates the drug-induced oxidative burst. Data are presented as mean ± SD from three independent experiments (n = 3).

**Supplementary Figure 5. Systematic safety evaluation and quantitative analysis of tumor immunohistochemistry.** (**A, B**) Tumor volume kinetics (**A**) and visual morphology images (**B**) of ABC-subtype DLBCL murine models. **(C)** Comprehensive hematological profiling in the ABC-subtype DLBCL xenograft model. Comparison of routine blood parameters between the vehicle control and Z2-A-Z2-treated groups, including white blood cell (WBC) count, red blood cell (RBC) count, neutrophil count (Neu), monocyte count(mon), lymphocyte count (lym), and hemoglobin (HGB) levels. **(D, E)** Assessment of renal and hepatic function in the ABC-subtype CDX model. Serum levels of creatinine (Cr) and blood urea nitrogen (BUN) were measured to evaluate renal integrity **(D)**, while alanine aminotransferase (ALT) and aspartate aminotransferase (AST) levels were analyzed to assess hepatic function **(E)**. **(F)** Quantitative histological analysis of resected tissue. Bar graphs represent the statistical quantification of the immunohistochemical (IHC) staining from Fig. 7G-H, including the percentage of Ki-67 and cleaved caspase-3 positive areas, as well as the relative expression levels of Cle-caspase3, HMG1, KIM-1 and NGAL. Data are presented as mean ± SD from at least three representative fields per section. (**G**) Quantitative histological analysis of resected tumor tissues. Bar graphs represent the statistical quantification of the immunohistochemical (IHC) staining from **Fig. 7J**, including the percentage of Ki-67 and cleaved caspase-3 positive areas, as well as the relative expression levels of IκBα, p50, and p65. Data are presented as mean ± SD from at least three representative fields per section.
